# New Benzofuran–Pyrazole-Based Compounds as Promising Antimicrobial Agents: Design, Synthesis, DNA Gyrase B Inhibition, and In Silico Studies

**DOI:** 10.3390/ph17121664

**Published:** 2024-12-10

**Authors:** Somaia S. Abd El-Karim, Manal M. Anwar, Yasmin M. Syam, Hassan M. Awad, Asmaa Negm El-Dein, Mohamed K. El-Ashrey, Hamad M. Alkahtani, Sameh H. Abdelwahed

**Affiliations:** 1 Department of Therapeutic Chemistry, Pharmaceutical and Drug Industries Research Institute, National Research Centre, Dokki, Cairo 12622, Egypt; ssabdelkarim@gmail.com (S.S.A.E.-K.); manal.hasan52@live.com (M.M.A.); ym.syam@nrc.sci.eg (Y.M.S.); 2 Chemistry of Natural and Microbial Products Department, Pharmaceutical and Drug Industries Research Institute, National Research Centre, Dokki, Cairo 12622, Egypt; awadmmhassan@gmail.com (H.M.A.); asmaanegm_084@yahoo.com (A.N.E.-D.); 3 Pharmaceutical Chemistry Department, Faculty of Pharmacy, Cairo University, Kasr Elini St., Cairo 11562, Egypt; mohamed.elashrey@pharma.cu.edu.eg; 4 Medicinal Chemistry Department, Faculty of Pharmacy, King Salman International University (KSIU), South Sinai 46612, Egypt; 5 Department of Pharmaceutical Chemistry, College of Pharmacy, King Saud University, P.O. Box 2457, Riyadh 11451, Saudi Arabia; ahamad@ksu.edu.sa; 6 Department of Chemistry, Prairie View A&M University, Prairie View, TX 77446, USA; 7 Department of Chemistry, Texas A&M University, College Station, TX 77843, USA

**Keywords:** benzofuran–pyrazole scaffold, antimicrobial activity, antioxidant, in vitro anti-inflammatory DNA gyrase enzyme, computational studies

## Abstract

Background/Objectives: The alarming rise in antibiotic resistance necessitates the discovery of novel antimicrobial agents. This study aims to design, synthesize, and evaluate new benzofuran–pyrazole-based compounds for their antimicrobial, antioxidant, and anti-inflammatory properties. Methods: New benzofuran–pyrazole hybrid molecules were synthesized using the Vilsmeier–Haach reaction and other chemical processes. The structures of the synthesized compounds were confirmed through micro-analytical and spectral analyses. Their antimicrobial activities were assessed against various bacterial and fungal strains, while antioxidant and anti-inflammatory properties were evaluated using DPPH-free radical scavenging and HRBC membrane stabilization assays, respectively. The most promising compounds were further tested for DNA gyrase B inhibition. Results: Compounds **9**, **10**, and **11b**–**d** exhibited significant broad-spectrum antimicrobial activity with MIC values ranging from 2.50 to 20 µg/mL. Compounds **4, 6, 9, 11b,** and **11d** demonstrated high antioxidant activity, with DPPH scavenging percentages between 84.16% and 90.52%. Most compounds showed substantial anti-inflammatory effects, with HRBC membrane stabilization percentages ranging from 86.70% to 99.25%. Compound **9** notably inhibited E. coli DNA gyrase B with an IC50 of 9.80 µM, comparable to ciprofloxacin. Conclusions: The benzofuran–pyrazole-based compounds, particularly compound **9**, show great potential as new antimicrobial agents due to their broad-spectrum activity and potent DNA gyrase B inhibition. These findings support further development and optimization of these compounds for clinical applications.

## 1. Introduction

Bacterial diseases are considered a global threat due to the fact that many bacterial isolates are increasing resistance to many antibiotics. Approximately 700,000 individuals worldwide pass away from incurable illnesses each year [1,2].

Experts predict that if we do not solve the issue of multiple-antibiotic resistance by 2050, it will claim the lives of over 10 million people worldwide. Different pathogenic microbes, such as viruses, yeasts, bacteria, and fungi, are frequently the cause of severe infections, which significantly exacerbate suffering in those with weakened immune systems and cause severe morbidity and mortality [3,4,5]. Antibiotics are effective tools in the fight against pathogenic bacteria by killing or suppressing their growth, but their misuse contributes to the emergence of antibiotic resistance, which has detrimental effects on human health [6]. The emergence and widespread distribution of drug-resistant bacteria, such as multi-drug-resistant (MDR) and pan-drug-resistant pathogens, underscores the urgent need to develop new drugs that are effective against both drug-sensitive and drug-resistant Gram-positive and Gram-negative pathogens [7,8,9].

Heterocyclic ring systems are powerful backbones with many biological characteristics due to their similarities to various natural bioactive molecules [10]. Benzofuran is a fundamental structural unit in a variety of biologically active natural and synthesized products [11], covering a wide range of categories such as antiviral, antibacterial, antifungal, antiparasitic, anti-TB, anticancer, anti-Alzheimer’s disease, analgesic, anti-inflammatory, and antihyperglycemic activities [12,13,14,15,16,17,18,19].

Recently, numerous derivatives bearing benzofuran nuclei hybridized with different heterocyclic cores have been designed, synthesized, and tested for their anti-microbial activities, and some of them showed promising potency against a panel of Gram-positive, Gram-negative, and fungal strains [20,21,22,23,24,25,26,27].

In medicinal chemistry, the pyrazole ring structure is extensively spotted as a pharmacophore, and the fortification of the modern organic synthesis toolbox demands synthetic tactics to generate its derivatives. Considering the wide range of biological activities of pyrazoles and their presence in naturally occurring compounds, pyrazole has been the topic of research for thousands of researchers all over the world [28,29,30,31]. Many studies have focused on the antibacterial action of various pyrazole-based derivatives against drug-resistant bacteria and fungi owing to their interactions with DNA, enzymes, and receptors [32,33].

**Figure 1:**
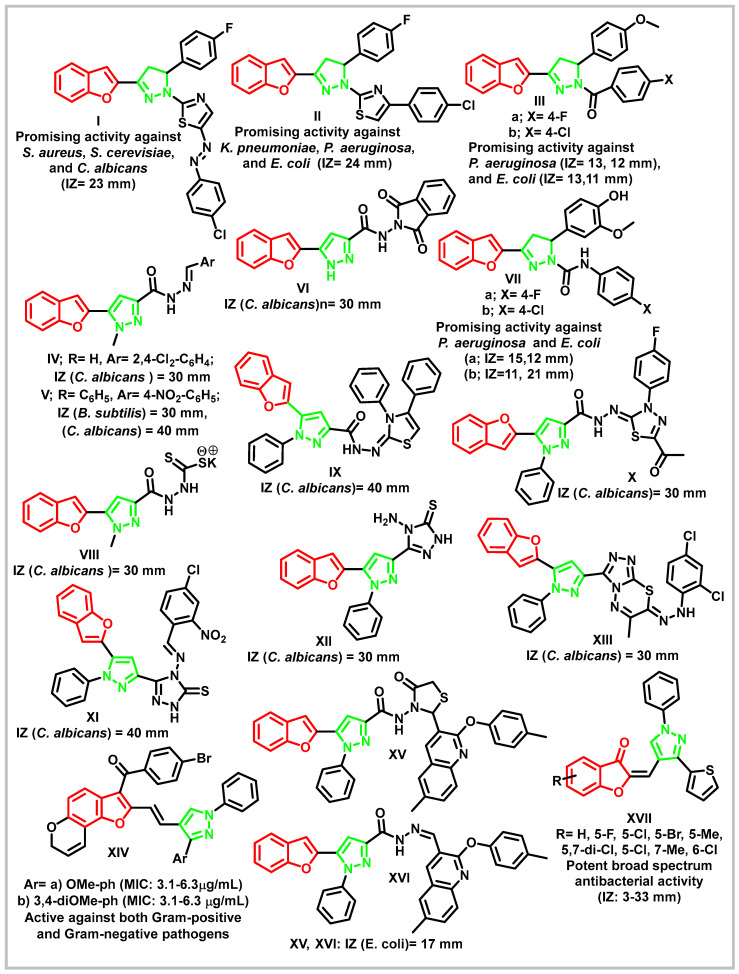
Examples of different molecules bearing benzofuran–pyrazole/pyrazoline scaffolds conjugated with heterocyclic rings possessing significant antimicrobial activities.

Recent studies have identified the hybridization of the benzofuran ring with pyrazole nucleus as a medically promising agent, serving as a crucial scaffold in the design and synthesis of promising antimicrobial, antioxidant, and anti-inflammatory candidates. Numerous benzofuran–pyrazole-based derivatives possess excellent efficacy as antimicrobial agents. Figure 1 shows different compounds derived from benzofuran–pyrazole scaffolds that have strong antimicrobial, antioxidant, and anti-inflammatory properties [34,35,36,37,38,39,40].

The topoisomerase enzyme is divided into three types in prokaryotic cells: type I, type II, and type III; in eukaryotes, type III is absent [41]. Type II has been divided into DNA gyrase (Gyrase A (GyrA) and B (GyrB), the two subunits of heterotetrameric DNA gyrase) and Topoisomerase IV [42]. Multiple studies have focused on type II subunits due to their specificity in the operation of bacterial reproduction. The tyrosine residue in the GyrA subunit is considered the active site amino acid, which is important for releasing the twist in the DNA (negative supercoiling), as well as reunion (positive supercoiling), whereas the GyrB subunit has an active site that is responsible for ATPase potency, providing energy for DNA supercoiling.

DNA gyrase plays a unique function since it depends on negative supercoiling, a process that keeps balance in bacterial DNA replication and prevents positive supercoiling from over-twisting DNA and breaking DNA strands. Accordingly, DNA gyrase has a bright future for the survival of bacteria. The significant amounts of DNA gyrase in prokaryotes like bacteria and their absence in human cells make them a selective target for newly developed molecules preserving both antibacterial and anti-MDR efficacy [43]. On the other hand, different mutations in gyrase lead to bacterial resistance, which results in traditional drug candidates not working properly. Based on these aspects, DNA gyrase inhibitors are driving the interest of researchers towards designing and synthesizing further potent candidates with higher DNA gyrase inhibitory activity and improving their toxicity profiles.

A crucial strategy in drug development is molecular hybridization, which blends many bioactive pharmacophores into a single molecular entity to create compounds with enhanced bioactivity. Additionally, it has been recognized that nitrogen- and oxygen-containing heterocycles play an important function as structural motifs in the creation of new antibacterial medicines due to their ability to interact with biological macromolecules like enzymes, proteins, and DNA [44,45].

**Figure 2:**
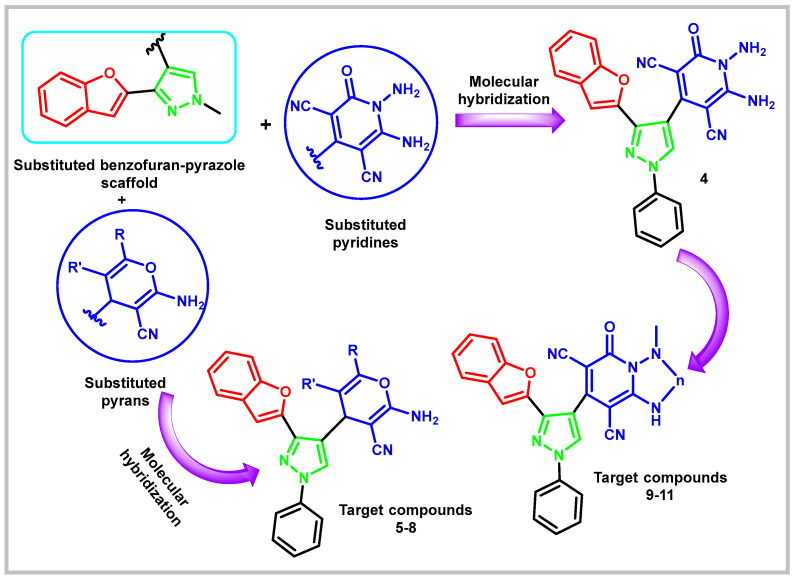
Development of new benzofuran–pyrazole hybrids of potential antimicrobial activity.

Therefore, this study focuses on the design and synthesis of new hybrid molecules that combine a benzofuran–pyrazole framework with various heterocycles, including pyridine, pyran, chromene, pyrano-pyrazole, pyrido-triazine, and triazolo-pyridine nuclei (Figure 2). The molecular structures of the new hybrids have been fully characterized. The antimicrobial characteristics of these compounds were examined against various fungal isolates and Gram-positive and Gram-negative bacteria. The new hybrids were also evaluated as in vitro antioxidants and anti-inflammatory agents. The most promising compounds, **9** and **10**, were selected as representative examples to study their suppression effects against the *E. coli* DNA gyrase B enzyme. Molecular docking and ADMET studies were also performed for the most active, compound **9**, using *E. coli* DNA gyrase B as the target enzyme.

## 2. Results and Discussion

### 2.1. Chemistry

**Scheme 1:**
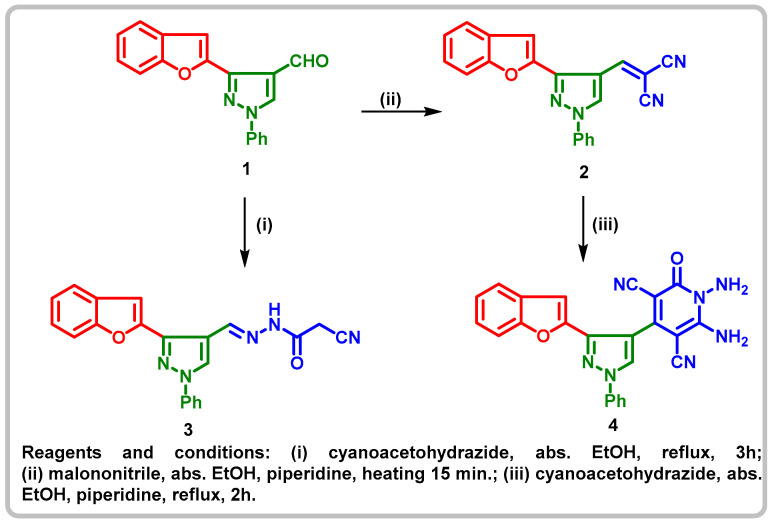
Synthesis of benzofuran-1*H*-pyrazol-2-oxopyridine-3,5-dicarbonitrile derivative **4**.

**Scheme 2:**
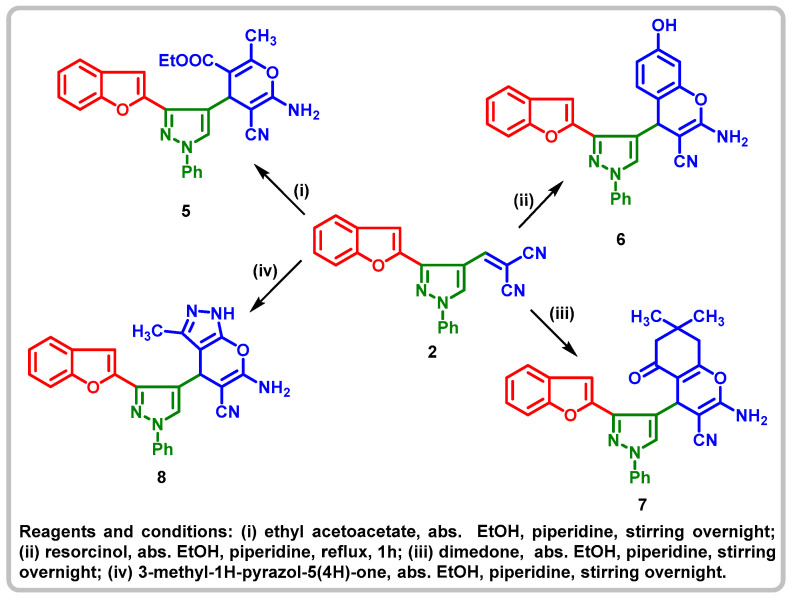
Synthesis of pyrano, chromene, tetrahydrochromene, pyrano[2,3-*c*]pyrazole derivatives.

**Scheme 3:**
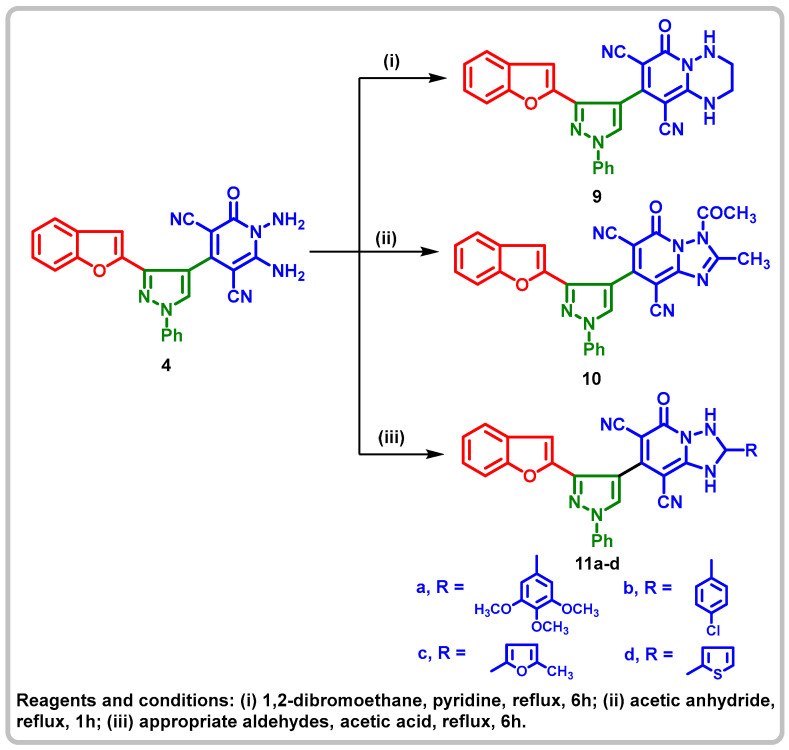
Synthesis of pyrido[1,2-*b*][1,2,4]triazine and [1,2,4]triazolo[1,5-*a*]pyridine derivatives.

Scheme 1, Scheme 2 and Scheme 3 depict the synthetic pathways adopted for the preparation of the new benzofuranpyrazole hybrid derivatives in this study. Using the Vilsmeier–Haach reaction, the key starting material 3-(benzofuran-2-yl)-1-phenyl-1H-pyrazole-4-carbaldehyde (**1**) was prepared according to the reported method [17]. Compound **1** was condensed with malonitrile to form 2-((3-(benzofuran-2-yl)-1-phenyl-1H-pyrazol-4-yl)methylene) malonitrile (**2**) [17]. The compound 1,6-diamino-4-(3-(benzofuran-2-yl)-1-phenyl-1H-pyrazol-4-yl)-1,2-dihydro-2-oxopyridine-3,5-dicarbonitrile (**4**) was prepared by refluxing the arylidine analogue **2** with cyanoacetohydrazide in absolute ethanol containing a catalytic amount of piperidine. On the other hand, direct condensation of the key starting aldehyde **1** with cyanoacetohydrazide afforded the cyanoacetohydrazide derivative **3.** The elemental analyses and spectral data confirmed the molecular structures of the synthesized derivatives **3** and **4**. The IR spectrum of compound **3** showed characteristic absorption bands at 3261, 2266, and 1678 cm^−1^ that are attributed to NH, CN, and C=O groups, respectively. Its ^1^H NMR spectrum also showed four singlet signals at δ_H_ 3.86, 4.23, 8.50, 8.58, 9.04, 9.05, and 11.86 ppm. These were for the methylene group, the azomethine CH=N (E and Z isomers), pyrazole-H5, and NH protons, in that order. There were also multiplet signals for aromatic protons at δ_H_ 7.34–8.02 ppm. Also, the IR spectrum of compound **4** displayed strong absorption bands at 3300, 3241, 3196, 3131 (2NH_2_), 2218 (2CN), and 1664 cm^−1^ (lactamic C=O). The ^1^H NMR spectrum of the same compound showed two D_2_O exchangeable singlet signals at δ 5.72 and 8.58 ppm due to -C-NH_2_ and -N-NH_2_ protons, respectively. This result confirms the difference in the nucleophilicity between the two amino groups. Thus, it is expected that the hydrazide β-nitrogen (-N-NH_2_) is more nucleophilic and will react more rapidly with the electron-deficient carbon than the second amino group (-C-NH_2_). The aromatic protons appeared as a multiplet signal in the range δ 7.17–8.01 ppm, while the pyrazole-H5 appeared as a singlet at δ 9.11 ppm. The mass spectrum further confirmed compound **4**, agreeing well with the assigned structure and displaying the correct molecular ion peak at *m*/*z* 433 (Scheme 1).

Moreover, condensation of the malononitrile derivative **2** with ethyl acetoacetate in the presence of a few drops of piperidine as a catalyst yielded the corresponding ethyl 6-amino-5-cyano-2-methyl-4*H*-pyran-3-carboxylate **5**. Furthermore, the treatment of the arylidine malononitrile derivative **2** with resorcinol and/or dimedone in absolute ethanol containing a catalytic amount of piperidine yielded the chromene derivatives **6**, **7**. Moreover, in order to obtain the fused pyrano [2,3-*c*]pyrazole compound **8**, the key intermediate **2** was treated with 3-methyl-1*H*-pyrazol-5(4*H*)-one derivative in absolute ethanol containing a few drops of piperidine (Scheme 2).

The structure of the new pyrano derivative **5** was elucidated on the basis of elemental and spectral data. For example, ^1^H NMR spectrum of compound **5** showed the characteristic triplet/quartet signals of the carboxylate group at *δ* 0.90 and 3.91 ppm, respectively. Additionally, the ^1^H NMR spectrum of compound **5** revealed four singlets at approximately *δ* 2.28, 4.45, 5.04, and 7.71 ppm, which correspond to the protons of CH_3_, NH_2_, pyran-H4, and pyrazole-H5, respectively, in addition to the multiplet signals of the aromatic protons, which appeared in the range of approximately *δ* 7.17–7.70 ppm. Its ^13^C NMR spectrum displayed three signals at *δ* 13.87, 18.58, and 61.79 ppm related to the methyl and ethyl carbons, respectively, besides the other signals attributed to the expected carbons of the molecule. The chemical structures of the obtained chromene and tetrahydrochromene derivatives **6** and **7** were confirmed by elemental and spectral analyses. For example, the IR spectrum of compound **6** represented absorption bands at 3325, 3204, 3135, and 2197 cm^−1^ that contributed to OH, NH_2_, and CN groups, respectively. The ^1^H NMR spectrum of the same analogue displayed three singlets at *δ* 5.21, 7.19, and 8.61 ppm attributed to the pyran-H4, OH, and pyrazole-H5, respectively. While the ^1^H NMR spectrum of compound **7** exhibited two singlet signals at *δ* 0.64 and 0.93 ppm referring to two methyl groups, there were also three singlet signals at *δ* 4.69, 6.99, and 8.53 ppm, indicating the protons chromene-H4, the NH_2_ group, and pyrazole-H5, respectively. In addition, the ^13^C NMR spectrum of **7** represented, besides the expected cyano and aromatic signals, characteristic signals at *δ* 26.59, 26.97, 28.86, 32.00, 50.52, 57.96, and 196.34 ppm due to the carbons of 2-CH_3_, CH_2_, chromene-C7, chromene-C6, chromene-C3, and C=O, respectively. The elemental and spectral data confirmed the chemical structure of compound **8**. The IR spectrum of the latter compound **8** showed characteristic absorption bands in the range 3393–3176 cm^−1^ due to NH and NH_2_, and at 2186 cm^−1^ due to the CN group. The ^1^H NMR spectrum exhibited a singlet signal at *δ* 1.83 ppm attributed to CH_3_, besides the other signals that appeared in their expected regions. The ^13^C NMR spectrum represented a signal at *δ* 10.25 ppm assignable to the CH_3_ group, in addition to 24 other signals representing the cyano and aromatic carbons of the molecule (Scheme 2).

*o*-Diamines are ready-made nucleophilic centers for the synthesis of fused nitrogen heterocyclic rings. Thus, diaminopyridone **4** was a useful building block for the synthesis of nitrogen bridge-head pyrido-triazine and/or pyrido-triazole derivatives **9**, **10**, **11a**–**d**, respectively. The treatment of diaminopyridone **4** with 1,2-dibromoethane in pyridine produced the corresponding tetrahydro-1*H*-pyrido[1,2-*b*][1,2,4]triazine derivative **9**. Furthermore, the 1,2,4-triazolo[1,5-*a*]pyridine and tetrahydro-[1,2,4]-triazolo[1,5-*a*]pyridine derivatives **10**, **11a**–**d** were obtained by boiling of the diaminopyridone derivative **4** in acetic anhydride and/or its refluxing with different aldehydes in acetic acid, respectively (Scheme 3).

Elemental analysis and spectral data (IR, MS, ^1^H and ^13^C NMR spectra) confirmed the reaction product. The IR spectrum of compound **9** showed absorption bands at 3189, 3127, and 2213 cm^−1^ due to 2NH and 2CN groups, respectively. In addition, its ^1^H NMR spectrum displayed the methylene protons of 2CH_2_ as two multiplets at *δ* 2.07 and 2.43 ppm, in addition to the aromatic proton pyrazole-H5 and the 2NH protons that appeared at their expected regions. Furthermore, the ^13^C NMR spectrum of compound **9** exhibited 25 carbon signals, and the most important signals appeared at *δ* 31.12, 71.45, and 161.64 ppm, characteristic of the two methylene and carbonyl carbons, respectively. The IR spectrum of compound **10** showed the disappearance of the NH_2_ bands of the parent diaminopyridone **4** and the presence of two absorption bands at 2223 and 1671 cm^−1^ referring to 2CN and 2CO groups, respectively. Also, the ^1^H NMR spectrum of compound **10** revealed, in addition to the aromatic protons and pyrazole-H5, two characteristic singlet signals at *δ* 1.92 and 2.09 ppm attributed to the methyl and acetyl protons, respectively, while its ^13^C NMR spectrum displayed 24 carbon signals, representing the methyl and acetyl carbons at *δ* 21.56 and 31.19 ppm, respectively. At the same time, the elemental analyses and spectral data were consistent with the proposed tetrahydro-[1,2,4]-triazolo[1,5-*a*]pyridine derivatives **11a**–**d**. For example, the ^1^H NMR spectrum of compound **11a** revealed two singlets at δ 3.79 and 3.89 ppm assignable to 3 (OCH_3_) and three other singlet signals at *δ* 8.56, 8.90, and 9.13 ppm attributable to the respective protons of 2NH, triazole-H3, and pyrazole-H5, respectively. Furthermore, the ^1^H NMR spectrum of **11c** exhibited, besides the expected signals of the aromatic protons, four singlets at *δ* 2.40, 8.70, 9.07, and 9.12 ppm due to the protons of CH_3_, 2NH, and pyrazole-H5, respectively. In addition, the furan-H3 and -H4 appeared as two doublets at *δ* 6.51 and 6.96 ppm, respectively. Mass spectra of **11a**–**d** showed the molecular ion peaks, which were in agreement with their molecular formulae (Scheme 3).

### 2.2. Biological Evaluation

#### 2.2.1. Antimicrobial Activity Determination

The in vitro antimicrobial activity of the new target compounds **2**–**11a**–**d** was evaluated against two fungal isolates, *F. solani* and *C. albicans* ATCC-10231, and four bacterial isolates, *S. aureus* ATCC 6538 and *B. cereus* ATCC 11778 as Gram-positive bacteria and *E. coli* 25922 and *P. aeruginosa* ATCC 27853 as Gram-negative bacteria. These specific strains were chosen because of their ability to form biofilms and their major impact on human health. Using the agar-well diffusion method [46], the average diameter of the inhibition zones in millimeters was assessed for each tested compound (10 µg/mL) against each type of microbial growth surrounding the discs (Table 1).

Also, the double-sequence dilution method [47,48,49] was used to find the minimum inhibitory concentration (MIC) values (given in µM) for the promising compounds with an IZ of 15 mm or more (**4**, **7**, **8**, **9**, **10**, **11b**–**d**) against all the tested isolates. Novobiocin (30 µg) and clotrimazole (50 µg) were utilized as the standard antibiotic and antifungal drugs, respectively, and the results are recorded in Table 2.

Based on the MIC results, it was noticed that all of the tested strains were susceptible to the antibacterial characteristics of the examined compounds. It was detected that the nitrogen bridge-head pyrido-triazine and/or pyrido-triazole derivatives **9**, **10**, and **11b**–**d** were the most promising candidates as broad-spectrum antimicrobial members. While it showed an equivalent activity to clotrimazole against the examined fungal strains (MIC = 20, 16 µg/mL, MIC_Clotrimazole_ = 20, 14 µg/mL), the six-membered pyrido-triazine compound **9** produced more significant potency than novobiocin against the tested positive and negative bacterial isolates (MIC = 2.50–17.60 µg/mL, MIC_novobiocin_ = 3.49-18.6 µg/mL). On the other hand, the 2-methyl-triazolo[1,5-*a*]pyridine derivative **10** has emerged as a potent antifungal candidate. It showed more significant activity against the fungal strain *F. solani* than clotrimazole, with an MIC value of 16 µg/mL, and equivalent antifungal activity to the reference drug against the tested *C. albicans* isolates, with an MIC value of 14 µg/mL. The antibacterial efficacy of the latter derivative appears to be slightly lower than that of the reference novobiocin, with MIC values ranging from 3.49 to 20 μg/mL.

Interestingly, changing the 2-methyl group in compound **10** to different phenyl groups in compounds **11b**,**c** increased their antifungal activity more than clotrimazole, with MIC values ranging from 16 to 19 μg/mL. However, they decreased their antibacterial activity against the tested bacterial isolates, with MIC values ranging from 5.11 to 20 μg/mL, which was lower than novobiocin. Conversely, the rest of the target compounds, **4**, **7**, and **8,** exhibited moderate antimicrobial activity, with MIC values ranging from 14 to 30 μM. Taken together, it could be concluded that the pyrido-triazine and/or pyrido-triazole ring system attached to the pyrazol–benzofuran scaffold is a highly recommended feature for gaining significant broad-spectrum antimicrobial efficacy.

#### 2.2.2. Antioxidant Activity

Reactive oxygen and nitrogen species (ROS/RNS) are naturally produced through cellular metabolism. They act as “redox messengers” in signaling pathways and are helpful against infectious pathogens, but they can also cause detrimental oxidative stress [50]. Numerous neurological diseases, cancer, diabetes, high-blood pressure issues, infectious diseases, and cardiovascular diseases are believed to be associated with free radical damage [51,52,53]. Antioxidants are compounds that reduce free radical concentrations and thus act as a protective barrier against the damage they cause to the body, which is why they are crucial in the prevention of various diseases.

**Figure 3:**
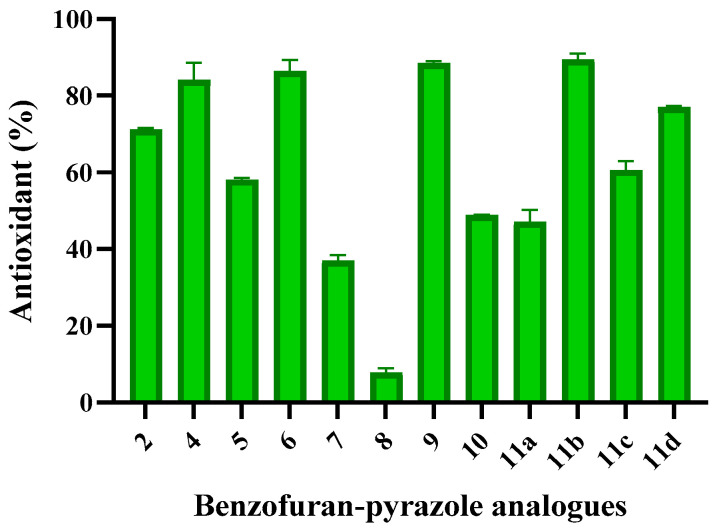
Antioxidant activities of the new target benzofuran–pyrazole-based derivatives.

Many processes have been described to evaluate the antioxidant activity of specific compounds, but the most widely documented relates to the 2,2-diphenyl-1-picrylhydrazyl (DPPH) radical [53] using the DPPH-free radical scavenging assay utilizing ascorbic acid as a standard drug, and the results are presented in Table 3. The obtained antioxidant activity of the tested compounds varied from moderate to potent activity in comparison with ascorbic acid. The most potent scavenging activity was produced by the compounds **4**, **6**, **9**, **11b**, and **11d** (%DPPH scavenging activity = 84.16, 86.42, 85.87, 90.52, 88.56, and 89.42%, respectively). On the other hand, the rest of the derivatives appeared to be moderate to weak antioxidant candidates (Figure 3). Fortunately, the most potent antimicrobial pyrido-triazine derivative **9** produced an effective radical scavenging capacity (88.56 ± 0.43%).

#### 2.2.3. Anti-Inflammatory Assay

The new target compounds were assessed for their in vitro anti-inflammatory potential via the Human Red Blood Cell (HRBC) membrane stabilization process. The anti-inflammatory effectiveness of the compounds was determined by regulating the suppression of hypotonicity-induced HRBC membrane lysis, which is an inflammatory response.

The HRBC membrane stability test relies on the discovery that non-steroidal anti-inflammatory drugs prevent erythrocyte lysis, most likely by maintaining the cell membrane’s stability. Inflammatory symptoms may be alleviated by compounds that stop the lysis of the HRBC membrane brought on by the release of hydrolytic enzymes from lysosomes [54,55,56,57].

The mechanism of action for a compound’s membrane protection may involve its binding to HRBC membranes and altering the charges on the cell surface. Accordingly, it was of interest to predict the in vitro anti-inflammatory activity of the new analogues according to the reported method [54,55,56,57].

The percentage of HRBC membrane stabilization tests for the newly synthesized derivatives and the positive controls aspirin and diclofenac sodium were carried out, and the results are provided in Table 3.

**Figure 4:**
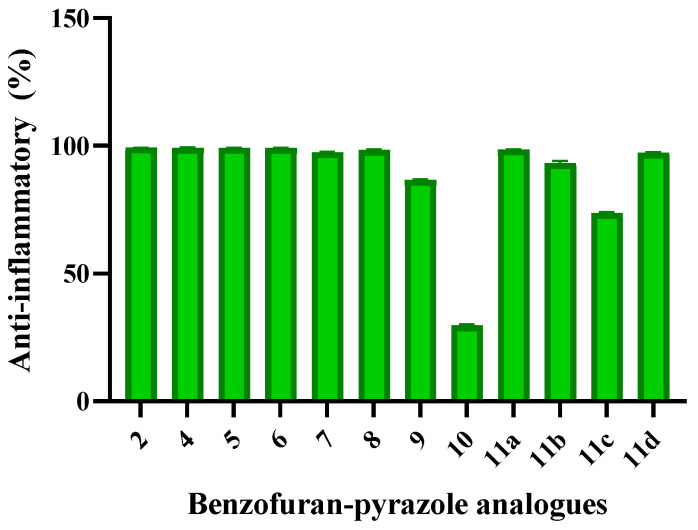
Anti-inflammatory activity of the new target benzofuran–pyrazole-based derivatives.

Most of the examined derivatives showed significant HRBC membrane stabilization and protection percentages ranging from 86.70 ± 0.259 to 99.25 ± 0.108%. Unfortunately, a lower protection percentage and weak activity were determined by compounds **11c** and **10** (73.67 ± 0.388 and 29.67 ± 0.496%, respectively). Interestingly, the most active antimicrobial analogue **9** showed a promising protection percentage (86.70 ± 0.259%) (Figure 4).

#### 2.2.4. *E. coli* DNA Gyrase B Suppression Effect

The compounds that exhibited the most promising antimicrobial, antioxidant, and anti-inflammatory activities, 9 and 10, were evaluated for in vitro suppression effects against *E. coli* DNA gyrase B as a trial to find out their modes of action as antimicrobial candidates [58,59], where ciprofloxacin served as a standard drug (Table 4).

The results demonstrated that the pyrido-triazine compound 9 produced a more potent suppression impact against *E. coli* DNA gyrase B than 10, but it was slightly less potent than ciprofloxacin, with IC_50_s of 9.80 ± 0.21, 32.20 ± 0.10, and 8.03 ± 0.03, respectively. The increase in the ring size attached to the benzofuran–pyrazole mother scaffold led to a greater suppression effect, which could be explained by the creation of an additional hydrophobic interaction with *E. coli* DNA gyrase B, resulting in an increase in the fitting of the compound in the active sites of the tested enzyme.

#### 2.2.5. In Vitro Cytotoxicity Assay

In continuation of this study, the safety profiles of the analogues 9 and 10 were assessed utilizing the calorimetric cell proliferation MTT [59] to evaluate the cytotoxic effects against the human diploid cell line WI-38, which is derived from lung tissues. Table 4 shows that the compounds’ safety profiles look good and are much better than those of the standard drug ciprofloxacin (IC_50_s = 163.3 ± 0.17, 170 ± 0.40, and 86.2 ± 0.03 µM, respectively).

### 2.3. In Silico Studies

#### 2.3.1. Molecular Docking

**Figure 5:**
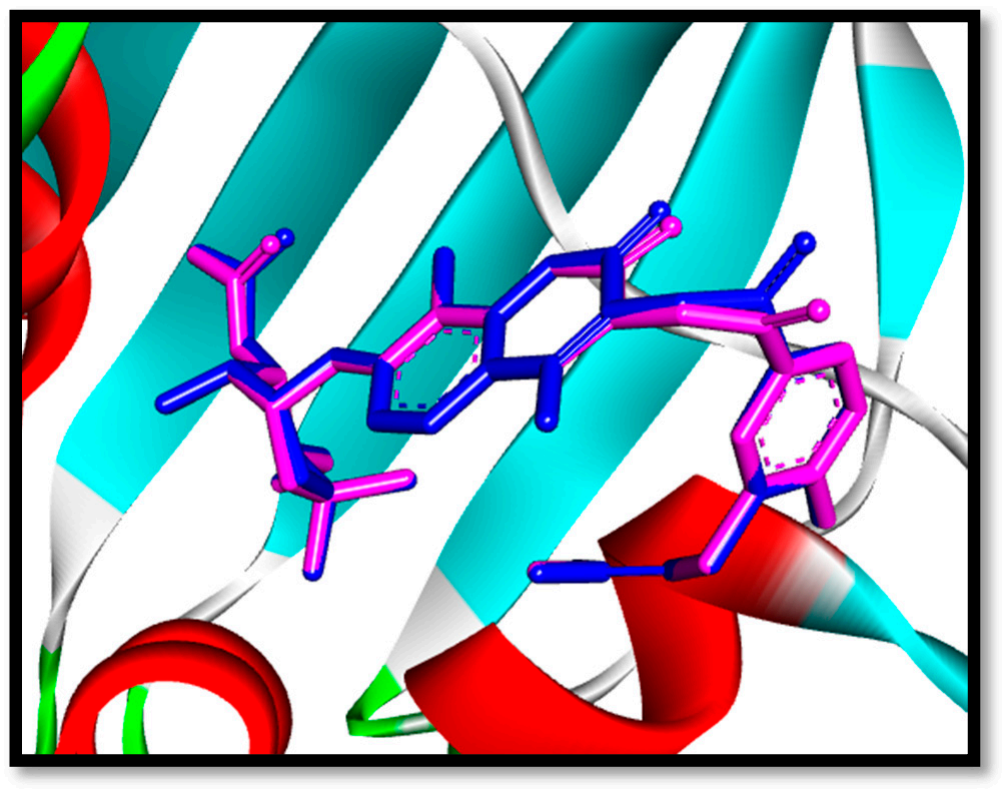
Three-dimensional representation of the superimposition of the co-crystallization (purple) and the docking pose (blue) of novobiocin in the active site of *E. coli* DNA gyrase.

Despite docking software’s capacity to identify potential binding modes between a ligand and its target, it is still an unreliable method that requires constant verification [60].^.^ As a result, the docking procedure was initially verified by re-docking the co-crystallized ligand close to the enzyme’s binding site. For the co-crystallized ligand of the DNA gyrase enzyme, the root mean square deviation (RMSD) was found to be 0.23, indicating a successful docking technique (Figure 5).

**Figure 6:**
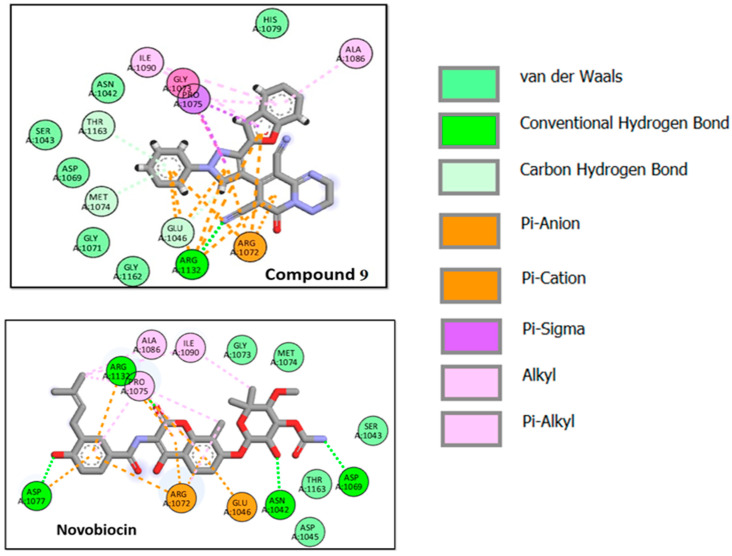
The binding interactions between the tested compound **9** and the *E. coli* DNA gyrase active site compared to novobiocin (pdb: 4URO).

Compound **9** has the ability to bind to the DNA gyrase active site in a comparable pattern to the co-crystallized ligand novobiocin. It shows a binding energy score of −8.9 kcal/mol. compared to −9.4 kcal/mol. for novobiocin. Compound **9** binds the key amino acids Arg1027, Asp81, Pro1075, and Glu1046 through pi-anion and pi-cation interactions, in addition to supportive hydrophobic interactions with different amino acid residues in the vicinity of the active site (Figure 6).

The binding energy scores of all the synthesized derivatives are summarized in Table 5.

#### 2.3.2. ADMET Studies

**Figure 7:**
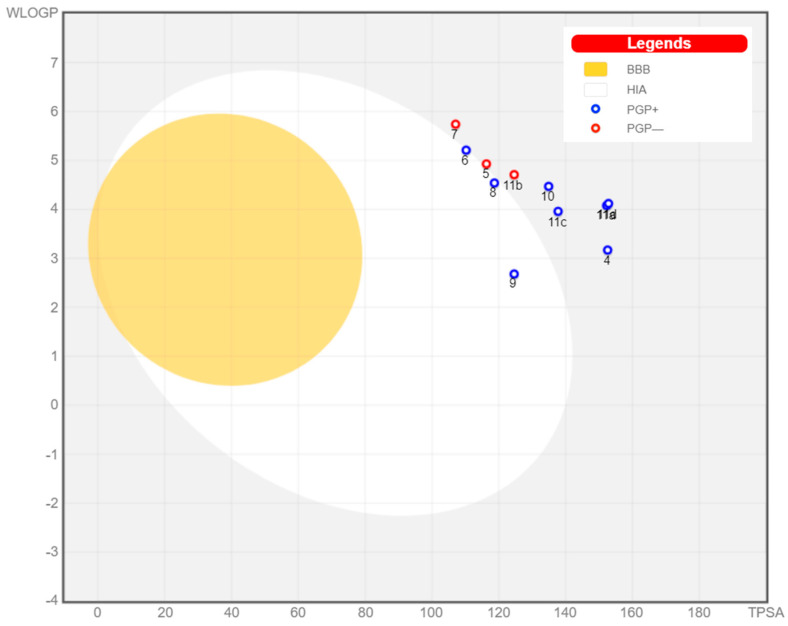
BOILED-Egg chart for compounds **4**–**11d** (the blue circle represents the tested compound).

Several measures are calculated by the SwissADME webserver [61] to predict oral bioavailability, pharmacokinetic properties, blood–brain barrier (BBB) penetration possibility, and permeability glycoprotein (PGP) binding affinity (Table 6). The bioavailability radar chart of the tested derivatives is illustrated in Figure 7. Compound **9** shows only one violation out of six measured parameters: lipophilicity (LIPO), size, polarity, insolubility (INSOL), insaturation (INSATU), and flexibility (FLEX); the violation was in the insaturation parameter (Figure 7). A BOILED-Egg chart was constructed; the white area indicates gastro-intestinal absorption, while the non-mutually exclusive yellow area indicates BBB penetration. The blue color indicates the high possibility of the tested compound being a substrate for PGP, which is an efflux protein responsible for uptake and efflux of many drugs; red color means the lower possibility of being a PGP substrate [62].

Among all the tested compounds, compound **9** shows the highest gastro-intestinal absorption with no BBB penetration, but it is a possible substrate for PGP (Figure 7). Eventually, applying Lipinski’s rule of five [63,64,65,66,67], which is a four-rule fulfilment criteria that is calculated to predict the drug-likeness of the tested derivative, **11** has a predicted logP value of 2.67 (<5), a molecular weight of 459.46 g/mol (<500), nine hydrogen bond acceptor groups (seven nitrogens + two oxygens) (<10), and only two hydrogen bond donor groups (2 NH) (<5) and so shows no violation of Lipinski’s rule, indicating its good pharmacokinetic profile.

## 3. Materials and Methods

### 3.1. Chemistry

All melting points are uncorrected and were taken in open capillary tubes using Electrothermal apparatus 9100. The instruments used to determine melting points, spectral data (IR, ^1^H NMR, ^13^C NMR, and mass), as well as chemical analyses, are included in a detailed description in the file in the Supporting Materials. 3-(Benzofuran-2-yl)-1-phenyl-1*H*-pyrazole-4-carbaldehyde (**1**) and 2-((3-(benzofuran-2-yl)-1-phenyl-1*H*-pyrazol-4-yl)methylene)malononitrile (**2**) were prepared according the reported method [17].

#### 3.1.1. N’-((3-(benzofuran-2-yl)-1-phenyl-1H-pyrazol-4-yl)methylene)-2-cyanoacetohy drazide (**3**)

A mixture of 3-(benzofuran-2-yl)-1-phenyl-1*H*-pyrazole-4-carbaldehyde (**1**) (2.88 g, 0.01 mol) and cyanoacetohydrazide (0.99 g, 0.01 mol) in absolute ethanol (30 mL) was refluxed for 3 h. Upon cooling, the formed precipitate was filtered, dried, and recrystallized from ethanol to give the title compound **3**.

Yield 61%, mp. 211–213 °C, yellow powder; IR (KBr, cm^−1^): 3261 (NH), 3059 (CH-arom.), 2959, 2917 (CH-aliph.), 2267 (CN), 1678 (C=O, amide); ^1^H NMR (500 MHz; DMSO-*d*_6_) *δ*_H_ 3.86 (s, 2H, CH_2_, Z-isomer), 4.23 (s, 2H, CH_2_, E-isomer), 7.34–7.43 (4H, m, Ar-H), 7.53–7.59 (2H, m, Ar-H), 7.64–7.74 (2H, m, Ar-H), 7.95–8.02 (2H, m, Ar-H), 8.50 (s,1H, CH=N, E-isomer), 8.58 (s,1H, CH=N, Z-isomer), 9.04 (s, 1H, pyrazole-H5, Z-isomer), 9.05 (s, 1H, pyrazole-H5, E-isomer), 11.86 (s, 1H, NH, D_2_O exchangeable); ^13^C NMR (125 MHz; DMSO-*d*_6_) E-isomer *δ*_C_ 24.10 (CH_2_), 105.20, 111.23, 116.17, 117.44, 118.90, 121.61, 123.51, 125.20, 127.44, 128.02, 128.95, 129.68, 137.40, 138.76, 140.46, 141.90, 149.05, 154.25 (aromatic-C), 164.46 (CO), ^13^C NMR (125 MHz; DMSO-*d*_6_) Z-isomer *δ*_C_ 24.86, 106.55, 111.23, 116.17, 117.44, 118.99, 121.61, 123.51, 125.20, 127.44, 128.21, 129.95, 129.68, 137.40, 138.76, 140.46, 141.99, 149.05, 154.25 (aromatic-C), 158.85 (CO); MS, *m*/*z* (%): 369 [M]^+^ (12), 285 [C_18_H_11_N_3_O]^+^ (8), 77 [C_6_H_5_]^+^ (100); Anal. For C_21_H_15_N_5_O_2_ (369.38): Calcd. C, 68.28; H, 4.09; N, 18.96; Found: C, 68.34; H, 4.24; N, 18.71.

#### 3.1.2. 1,6-Diamino-4-(3-(benzofuran-2-yl)-1-phenyl-1H-pyrazol-4-yl)-1,2-dihydro-2-oxopyridine-3,5-dicarbonitrile (**4**)

A mixture of 2-((3-(benzofuran-2-yl)-1-phenyl-1*H*-pyrazol-4-yl)methylene)malononitrile (**2**) (3.36 g, 0.01 mol) and cyanoacetohydrazide (0.99 g, 0.01 mol) in absolute ethanol (30 mL) containing 3–5 drops of piperidine was refluxed for 2 h. The white precipitate obtained during heating was filtered, dried, and recrystallized from ethanol to give the title compound **4**.

Yield 85%; mp 269–270 °C; IR (KBr, cm^−1^): 3393, 3315, 3210 (2NH_2_), 2217 (CN), 1661 (CO); ^1^H NMR (300 MHz; DMSO-*d*_6_) *δ*_H_ 5.72 (s, 2H, NH_2_, D_2_O exchangeable), 7.17 (s, 1H, Ar-H), 7.27–7.47 (m, 3H, Ar-H), 7.59–7.70 (m, 4H, Ar-H), 8.01 (d, 2H, ^3^*J* = 7.8 Hz, Ar-H), 8.58 (br, 2H, NH_2_, D_2_O exchangeable), 9.11 (s, 1H, pyrazole proton) ppm; ^13^C NMR (75 MHz; DMSO-*d*_6_) *δ*_C_ 75.52, 87.70, 104.91, 111.33, 115.32, 115.39, 116.24, 118.70, 121.58, 123.43, 125.21, 127.56, 128.15, 129.92, 138.63, 141.09, 148.04, 150.90, 154.13, 156.69, 159.26; MS, *m*/*z* (%): 433 [M]^+^ (37), 434 [M+1]^+^ (19), 77 [C_6_H_5_]^+^ (100). Anal. calcd. for C_24_H_15_N_7_O_2_ (433.43): C, 66.51; H, 3.49; N, 22.62; found: C, 66.32; H, 3.22; N, 22.43.

#### 3.1.3. Ethyl-6-amino-4-(3-(benzofuran-2-yl)-1-phenyl-1H-pyrazol-4-yl)-5-cyano-2-methyl-4H-pyran-3-carboxylate (**5**)

A mixture of compound **2** (0.68 g, 0.002 mol) and ethyl acetoacetate (0.26 mL, 0.002 mol) in absolute ethanol (20 mL) containing 2–3 drops of piperidine was stirred overnight at room temperature. The formed precipitate was filtered, dried, and recrystallized from ethanol to give the title compound **5**.

Yield 76%, mp. 196–198 °C, white powder; IR (KBr, cm^−1^): 3315, 3188, (NH_2_), 3062 (CH-arom.), 2980, 2924 (CH-aliph.), 2201 (CN), 1725 (C=O, ester); ^1^H NMR (300 MHz; CDCl_3_) *δ*_H_ 0.90 (t, 3H, ^3^*J* = 7.2 Hz, CO_2_CH_2_CH_3_), 2.28 (s, 3H, CH_3_), 3.91 (q, 2H, ^3^*J* = 6.8 Hz, CO_2_CH_2_CH_3_), 4.45 (s, 2H, NH_2_, D_2_O exchangeable), 5.04 (s,1H, pyran-H4), 7.17–7.29 (m, 4H, Ar-H), 7.39–7.50 (m, 3H, Ar-H), 7.56–7.59 (m, 1H, Ar-H), 7.67–7.70 (m, 2H, Ar-H), 7.71 (1H, s, pyrazole-H5); ^13^C NMR (75 MHz; CDCl_3_) *δ*_C_ 13.87 (CH_3_), 18.58 (CH_3_), 29.08 (pyran-C-4), 60.87 (pyran-C-5), 61.79 (CH_2_), 104.26, 107.65, 111.33, 119.02, 119.39, 121.32, 123.08, 124.41, 125.76, 127.02, 127.90, 128.76, 129.55, 139.76, 142.55, 150.36, 155.00, 156.56, 157.81 (aromatic-C), 166.02 (CO); MS, *m*/*z* (%): 466 [M]^+^ (3), 465 [M-1]^+^ (3), 77 [C_6_H_5_]^+^ (100); Anal. For C_27_H_22_N_4_O_4_ (466.49): Calcd. C, 69.52; H, 4.75; N, 12.01; Found: C, 69.81; H, 4.93; N, 12.21.

#### 3.1.4. 2-Amino-4-(3-(benzofuran-2-yl)-1-phenyl-1H-pyrazol-4-yl)-7-hydroxy-4H-chromene-3-carbonitrile (**6**)

A mixture of compound **2** (0.68 g, 0.002 mol) and resorcinol (0.22 g, 0.002 mol) in absolute ethanol (20 mL) containing 2–3 drops of piperidine was refluxed for 1 h. The formed precipitate during heating was filtered, dried, and recrystallized from ethanol to give the title compound **6**.

Yield 57%, mp. 252–254 °C, yellow powder; IR (KBr, cm^−1^): 3325, 3204, 3135 (OH, NH_2_), 3060 (CH-arom.), 2197 (CN); ^1^H NMR (300 MHz; DMSO-*d*_6_) *δ*_H_ 5.21 (s, 1H, pyran-H4), 6.41–6.46 (m, 2H, Ar-H), 6.83–6.87 (m, 3H, Ar-H), 7.19 (s, 1H, OH, D_2_O exchangeable), 7.24–7.36 (m, 4H, NH_2_, D_2_O exchangeable, Ar-H), 7.54 (t, 2H, ^3^*J* = 8.1 Hz, Ar-H), 7.63 (t, 2H, ^3^*J* = 8.7 Hz, Ar-H), 7.95 (d, 2H, ^3^*J* = 7.5 Hz, Ar-H), 8.61 (1H, s, pyrazole-H5); ^13^C NMR (75 MHz; DMSO-*d*_6_) *δ*_C_ 30.25 (Chromene-C4), 55.36 (Chromene-C3), 102.13, 103.42, 111.23, 112.19, 112.82, 118.29, 120.93, 121.24, 123.27, 124.58, 126.73, 126.91, 128.10, 128.93, 129.61, 129.68, 139.08, 141.26, 148.96, 150.02, 154.08, 157.12, 160.22 (aromatic-C); MS, *m*/*z* (%): 446 [M]^+^ (5), 429 [M-OH]^+^ (42), 77 [C_6_H_5_]^+^ (100); Anal. For C_27_H_18_N_4_O_3_ (446.46): Calcd. C, 72.64; H, 4.06; N, 12.55; Found: C, 72.92; H, 4.27; N, 12.26.

#### 3.1.5. 2-Amino-4-(3-(benzofuran-2-yl)-1-phenyl-1H-pyrazol-4-yl)-5,6,7,8-tetrahydro-7,7-dimethyl-5-oxo-4H-chromene-3-carbonitrile (**7**)

A mixture of compound **2** (0.68 g, 0.002 mol) and dimedone (0.28 g, 0.002 mol) in absolute ethanol (20 mL) containing 2–3 drops of piperidine was stirred overnight at room temperature. The formed precipitate was filtered, dried, and recrystallized from ethanol to give the target compound **7**.

Yield 71%, mp. 264–266 °C, white powder; IR (KBr, cm^−1^): 3325, 3211 (NH_2_), 3056 (CH-arom.), 2959, 2927, 2870 (CH-aliph.), 2189 (CN); ^1^H NMR (300 MHz; DMSO-*d*_6_) *δ*_H_ 0.64 (s, 3H, CH_3_), 0.93 (s, 3H, CH_3_), 1.97–2.02 (m, 1H, CH_2_), 2.14–2.26 (m, 2H, CH_2_), 2.46 (br.s, 1H, CH_2_), 4.69 (s, 1H, pyran-H4), 6.99 (s, 2H, NH_2_, D_2_O exchangeable), 7.25–7.32 (m, 4H, Ar-H), 7.49 (t, 2H, ^3^*J* = 7.8 Hz, Ar-H), 7.64 (t, 2H, ^3^*J* = 7.8 Hz, Ar-H), 7.89 (d, 2H, ^3^*J* = 7.8 Hz, Ar-H), 8.53 (s, 1H, pyrazole-H5); ^13^C NMR (75 MHz; DMSO-*d*_6_) *δ*_C_ 26.11 (chromene-C4), 26.49 (CH_3_), 28.38 (CH_3_), 31.52 (chromene-C7), 50.04 (CH_2_), 57.48 (CH_2_), 103.69, 111.21, 111.54, 118.15, 119.96, 121.22, 123.20, 124.49, 126.25, 126.60, 128.23, 128.72, 129.62, 139.07, 141.19, 150.14, 154.20, 158.63, 126.53 (aromatic-C), 195.86 (CO); MS, *m*/*z* (%): 476 [M]^+^ (3), 410 [C_24_H_18_N_4_O3]^+^ (58), 326 [C_21_H_16_N_3_O]^+^ (85), 66 [C_5_H_6_]^+^ (100); Anal. For C_29_H_24_N_4_O_3_ (476.53): Calcd. C, 73.09; H, 5.08; N, 11.76; Found: C, 73.34; H, 5.28; N, 11.54.

#### 3.1.6. 6-Amino-4-(3-(benzofuran-2-yl)-1-phenyl-1H-pyrazol-4-yl)-3-methyl-1,4-dihydropyrano[2,3-c]pyrazole-5-carbonitrile (**8**)

A mixture of compound **2** (0.68 g, 0.002 mol) and 3-methyl-1*H*-pyrazol-5(4*H*)-one (0.20 g, 0.002 mol) in absolute ethanol (20 mL) containing 2–3 drops of piperidine was stirred at room temperature overnight. The formed precipitate was filtered, dried, and recrystallized from ethanol to give the target compound **8**.

Yield 75%, mp. 226–228 °C, white powder; IR (KBr, cm^−1^): 3393, 3306, 3176 (NH, NH_2_), 3054 (CH-arom.), 2922, 2858 (CH-aliph.), 2186 (CN); ^1^H NMR (300 MHz; DMSO-*d*_6_) *δ*_H_ 1.83 (3H, s, CH_3_), 5.17 (1H, s, pyran-H4), 6.89 (2H, s, NH_2_, D_2_O exchangeable), 7.21 (s,1H, Ar-H), 7.24–7.37 (m, 3H, Ar-H), 7.53 (t, 2H, ^3^*J* = 7.95 Hz, Ar-H), 7.64 (t, 2H, ^3^*J* = 7.8 Hz, Ar-H), 7.96 (d, 2H, ^3^*J* = 7.5 Hz, ^4^*J* = 1.2 Hz, Ar-H), 8.60 (1H, s, pyrazole-H5), 12.00 (1H, s, NH, D_2_O exchangeable); ^13^C NMR (75 MHz; DMSO-*d*_6_) δ_C_ 9.77 (CH_3_), 26.58 (pyran-C4), 56.45 (pyran-C3), 96.97 (CN), 103.55, 111.31, 118.21, 121.05, 121.18, 123.24, 124.53, 125.32, 126.68, 128.07, 128.74, 128.75, 129.64, 135.54, 139.09, 141.33, 150.03, 154.05, 154.86, 161.13, 171.18 (aromatic-C); MS, *m*/*z* (%): 434 [M]^+^ (5), 368 [C_22_H_16_N_4_O_2_]^+^ (100); Anal. For C_25_H_18_N_6_O_2_ (434.45): Calcd. C, 69.11; H, 4.18; N, 19.34; Found: C, 69.32; H, 4.35; N, 19.16.

#### 3.1.7. 8-(3-(Benzofuran-2-yl)-1-phenyl-1H-pyrazol-4-yl)-6-oxo-1,3,4,6-tetrahydro-2H-pyrido[1,2-b][1,2,4]triazine-7,9-dicarbonitrile (**9**)

A mixture of compound **4** (0.87 g, 0.002 mol) and 1,2-dibromoethane (0.18 mL, 0.002 mol) in pyridine (15 mL) was refluxed for 6 h. The reaction mixture was poured into ice-cold water; the formed precipitate was filtered, dried, and recrystallized from ethanol to give the title compound **9**.

Yield 93%, mp. 238–240 °C, white powder; IR (KBr, cm^−1^): 3189, 3127 (2NH), 3049 (CH-arom.), 2922, 2854 (CH-aliph.), 2213 (2CN), 1639 (C=O); ^1^H NMR (300 MHz; DMSO-*d*_6_) *δ*_H_ 2.07 (m, 2H, CH_2_), 2.43 (m, 2H, CH_2_), 6.94 (s, 1H, Ar-H), 7.24 (d, 1H, ^3^*J* = 7.5 Hz, Ar-H), 7.32 (t, 2H, ^3^*J* = 7.5 Hz, Ar-H), 7.42 (t, 1H, ^3^*J* = 7.2 Hz, Ar-H), 7.58–7.67 (m, 3H, Ar-H), 7.98–8.06 (m, 2H, Ar-H), 9.05 (1H, s, pyrazole-H5), 9.09 (s, 1H, NH, D_2_O exchangeable); ^13^C NMR (75 MHz; DMSO-*d*_6_) δ_C_ 30.70 (CH_2_), 70.97 (CH_2_), 78.01 (CN), 84.58 (CN), 104.66, 111.22, 116.45, 116.70, 118.53, 121.58, 123.25, 125.03, 127.10, 127.26, 128.19, 129.67, 129.85, 138.80, 141.24, 142.47, 145.58, 148.30, 152.46, 153.98, 155.86 (aromatic-C), 161.16 (CO); MS, *m*/*z* (%): 458 [M-1]^+^ (16), 457 [M-2]^+^ (20), 433 [M-C_2_H_2_]^+^ (40), 51 [C_4_H_3_]^+^ (100); Anal. For C_26_H_17_N_7_O_2_ (459.46): Calcd. C, 67.97; H, 3.73; N, 21.34; Found: C, 67.64; H, 3.51; N, 21.58.

#### 3.1.8. 3-Acetyl-7-(3-(benzofuran-2-yl)-1-phenyl-1H-pyrazol-4-yl)-3,5-dihydro-2-methyl-5-oxo-[1,2,4]triazolo[1,5-a]pyridine-6,8-dicarbonitrile (**10**)

A mixture of compound **4** (0.87 g, 0.002 mol) and acetic anhydride (10 mL) was refluxed for 1 h. Upon cooling, the formed precipitate was filtered, dried, and recrystallized from acetic acid to give the title compound **10**.

Yield 74%, mp. > 300 °C, yellow powder; IR (KBr, cm^−1^): 3051 (CH-arom.), 2925, 2846 (CH-aliph.), 2223 (2CN), 1671 (2C=O); ^1^H NMR (300 MHz; DMSO-*d*_6_) *δ*_H_ 1.92 (s, 3H, CH_3_), 2.09 (s, 3H, CH_3_), 7.04 (s, 1H, Ar-H), 7.26 (t, 1H, ^3^*J* = 7.65 Hz, Ar-H), 7.35 (t, 1H, ^3^*J* = 8.1 Hz, Ar-H), 7.44 (t, 1H, ^3^*J* = 7.35 Hz, Ar-H), 7.59–7.65 (m, 4H, Ar-H), 8.02 (d, 2H, ^3^*J* = 7.8 Hz, Ar-H), 9.09 (s, 1H, pyrazole-H5); ^13^C NMR (75 MHz; DMSO-*d*_6_) δ_C_ 21.09 (CH_3_), 31.71 (CH_3_), 76.69 (CN), 86.60 (CN), 104.91, 111.30, 115.71, 116.14, 117.35, 118.64, 121.56, 123.31, 125.11, 127.39, 128.22, 129.89, 138.76, 141.25, 146.83, 148.12, 150.85, 154.04, 155.18 (aromatic-C), 158.50 (CO); MS, *m*/*z* (%): 498 [M-1]^+^ (3), 418 [C_24_H_14_N_6_O_2_]^+^ (32), 56 [C_4_H_8_]^+^ (100); Anal. For C_28_H_17_N_7_O_3_ (499.48): Calcd. C, 67.33; H, 3.43; N, 19.63; Found: C, 67.52; H, 3.14; N, 19.80.

#### 3.1.9. 7-(3-(Benzofuran-2-yl)-1-phenyl-1H-pyrazol-4-yl)-1,2,3,5-tetrahydro-2-(substituted)-5-oxo-[1,2,4]triazolo[1,5-a]pyridine-6,8-dicarbonitrile **11a**–**d**

A mixture of compound **4** (0.87 g, 0.002 mol) and the appropriate aldehyde derivatives, namely 3,4,5-trimethoxybenzaldehyde, 4-chlorobenzaldehyde, 5-methylfuran-2-carbaldehyde, and/or thiophene-2-carbaldehyde (0.002 mol), in acetic acid (20 mL) was refluxed for 6–8 h. After cooling, the formed precipitate was filtered, dried, and recrystallized from acetic acid to give the title compounds **11a**–**d**.

##### 7-(3-(Benzofuran-2-yl)-1-phenyl-1H-pyrazol-4-yl)-1,2,3,5-tetrahydro-2-(3,4,5-trimethoxyphenyl)-5-oxo-[1,2,4]triazolo[1,5-a]pyridine-6,8-dicarbonitrile (**11a**)

Yield 83%, mp. 297–299 °C, white powder; IR (KBr, cm^−1^): 3299, 3214 (2NH), 3067 (CH-arom.), 2942, 2832 (CH-aliph.), 2221 (2CN), 1665 (C=O); ^1^H NMR (300 MHz; DMSO-*d*_6_) *δ*_H_ 3.79 (s, 3H, OCH_3_), 3.89 (s, 6H, 2(OCH_3_)), 7.27–7.48 (m, 6H, Ar-H), 7.63 (t, 2H, *J* = 7.95 Hz, Ar-H), 7.71 (d, 2H, *J* = 7.80 Hz, Ar-H), 8.03 (d, 2H, *J* = 7.80 Hz, Ar-H), 8.56 (br, 2H, 2NH, D_2_O exchangeable), 8.90 (s, 1H, triazole-H3), 9.13 (s, 1H, pyrazole-H5); ^13^C NMR (75 MHz; DMSO-*d*_6_) δ_C_ 56.17, 60.34, 84.06 (CN), 88.66 (CN), 107.32, 115.27, 115.29, 118.73, 121.70, 125.15, 125.22, 125.26, 125.28, 125.31, 125.75, 125.90, 126.25, 126.62, 126.91, 127.25, 128.29, 129.95, 140.40, 145.54, 146.81, 153.05, 154.13, 156.38, 158.11, 158.58, 158.94 (aromatic-C), 173.37 (CO); MS, *m*/*z* (%): 611 [M]^+^ (74), 610 [M-1]^+^ (29), 236 [C_11_H_14_N_3_O_3_]^+^ (100); Anal. For C_34_H_25_N_7_O_5_ (611.62): Calcd. C, 66.77; H, 4.12; N, 16.03; Found: C, 66.41; H, 4.33; N, 16.35.

##### 7-(3-(Benzofuran-2-yl)-1-phenyl-1H-pyrazol-4-yl)-2-(4-chlorophenyl)-1,2,3,5-tetrahydro-5-oxo-[1,2,4]triazolo[1,5-a]pyridine-6,8-dicarbonitrile (**11b**)

Yield 61%, mp. 221–223 °C, white powder; IR (KBr, cm^−1^): 3299, 3192 (2NH), 3055 (CH-arom.), 2218 (2CN), 1676 (C=O); ^1^H NMR (300 MHz; DMSO-*d*_6_) *δ*_H_ 7.28–7.32 (m, 2H, Ar-H), 7.38 (t, 1H, ^3^*J* = 7.2 Hz, Ar-H), 7.45 (t, 1H, ^3^*J* = 7.35 Hz, Ar-H), 7.45 (t, 2H, ^3^*J* = 7.95 Hz, Ar-H), 7.68 (d, 4H, ^3^*J* = 8.4 Hz, Ar-H), 7.99 (d, 2H, ^3^*J* = 7.8 Hz, Ar-H), 8.09 (d, 2H, ^3^*J* = 8.7 Hz, Ar-H), 8.62 (br., 2H, 2NH, D_2_O exchangeable), 9.07 (s, 1H, triazole-CH), 9.12 (s, 1H, pyrazole-H5); ^13^C NMR (75 MHz; DMSO-*d*_6_) δ_C_ 76.02 (CN), 88.63 (CN), 105.28, 111.44, 111.47, 115.26, 116.17, 118.76, 121.59, 123.43, 123.46, 125.23, 127.59, 127.61, 128.28, 129.09, 129.94, 130.67, 131.57, 138.06, 138.64, 147.87, 154.15, 156.33, 161.50 (aromatic-C), 171.44 (CO); MS, *m*/*z* (%): 555 [M]^+^ (3), 556 [M+1]^+^ (2), 418 [C_24_H_14_N_6_O_2_]^+^ (58), 137 [C_7_H_4_ClN]^+^ (100); Anal. For C_31_H_18_ClN_7_O_2_ (555.97): Calcd. C, 66.97; H, 3.26; N, 17.64; Found: C, 67.19; H, 3.42; N, 17.39.

##### 7-(3-(Benzofuran-2-yl)-1-phenyl-1H-pyrazol-4-yl)-1,2,3,5-tetrahydro-2-(5-methylfuran-2-yl)-5-oxo-[1,2,4]triazolo[1,5-a]pyridine-6,8-dicarbonitrile (**11c**)

Yield 85%, mp. 217–219 °C, grey powder; IR (KBr, cm^−1^): 3196, 3125 (2NH), 3062 (CH-arom.), 2929, 2850 (CH-aliph.), 2217 (2CN), 1664 (C=O); ^1^H NMR (300 MHz; DMSO-*d*_6_) *δ*_H_ 2.46 (s, 3H, CH_3_), 6.51 (d, 1H, *J* = 6.7 Hz, furan-H), 6.95 (s, 1H, furan-H), 7.17 (s, 1H, Ar-H), 7.24–7.47 (m, 4H, Ar-H), 7.62–7.69 (m, 2H, Ar-H), 7.99–8.01 (m, 2H, Ar-H), 8.44, 8.58 (2br., 2H, 2NH, D_2_O exchangeable), 8.68 (s, 1H, pyrazole-H5), 9.11 (s, 1H, triazole-CH); ^13^C NMR (75 MHz; DMSO-*d*_6_) δ_C_ 14.28 (CH_3_), 69.02 (CN), 76.39 (CN), 105.01, 111.90, 115.79, 115.86, 119.20, 122.05, 122.10, 123.74, 123.90, 125.52, 125.69, 126.31, 127.63, 128.71, 130.40, 139.10, 141.60, 146.32, 154.61, 154.73, 157.17, 159.20, 162.93 (aromatic-C), 165.01 (CO); MS, *m*/*z* (%): 525 [M]^+^ (21), 526 [M+1]^+^ (10), 82 [C_5_H_6_O]^+^ (100); Anal. For C_30_H_19_N_7_O_3_ (525.52): Calcd. C, 68.57; H, 3.64; N, 18.66; Found: C, 68.73; H, 3.49; N, 18.40.

##### 7-(3-(Benzofuran-2-yl)-1-phenyl-1H-pyrazol-4-yl)-1,2,3,5-tetrahydro-5-oxo-2-(thiophen-2-yl)-[1,2,4]triazolo[1,5-a]pyridine-6,8-dicarbonitrile (**11d**)

Yield 92%, mp. 194–196 °C, grey powder; IR (KBr, cm^−1^): 3214, 3136 (2NH), 3051 (CH-arom.), 2927, 2845 (CH-aliph.), 2219 (2CN), 1668 (C=O); ^1^H NMR (300 MHz; DMSO-d_6_) δ_H_ 7.17 (s, 1H, thiophene-H) 7.29–7.34 (4H, m, Ar-H), 7.59–7.68 (5H, m, Ar-H), 7.98–8.02 (3H, m, Ar-H, triazole-H3), 8.58 (2H, 2s, 2NH, D_2_O exchangeable), 9.10 (1H, s, pyrazole-H5), 9.14 (s, 1H, triazole-CH); ^13^C NMR (75 MHz; DMSO-d_6_) δ_C_ 75.50 (CN), 88.70 (CN), 104.91, 115.41, 118.74, 120.20, 121.58, 123.43, 125.22, 127.56, 128.15, 129.93, 138.64, 141.08, 142.03, 148.03, 150.91, 151.97, 156.41, 161.21 (aromatic-C), 168.64 (CO); MS, *m*/*z* (%): 527 [M]^+^ (42), 528 [M+1]^+^ (41), 443 [M-C_4_H_4_S]^+^ (45), 77 [C_6_H_5_]^+^ (100); Anal. For C_29_H_17_N_7_O_2_S (527.56): Calcd. C, 66.02; H, 3.25; N, 18.59; S, 6.08; Found: C, 66.28; H, 3.41; N, 18.72; S, 6.22.

### 3.2. Biological Evaluation

#### 3.2.1. In Vitro Antimicrobial Activity

The antibacterial screening bioassay was made by the agar well-diffusion method using Mueller–Hinton agar (Lab M Limited, Bury, Lancashire, UK), and then the plates were transferred to refrigerator for 1 h at 4 °C [47,48,49]. More detailed descriptions are available in the file in the Supporting Materials.

#### 3.2.2. DPPH Radical Scavenging Assay

The DPPH (1–diphenyl–2–picrylhydrazyl) scavenging activity of the sample was determined quantitatively according to the reported method [51,52,53]. More detailed descriptions are available in the file in the Supporting Materials.

#### 3.2.3. Human Red Blood Cell Stabilization Method

The human red blood cell (HRBC) membrane stabilization method was used to study the in vitro anti-inflammatory activity of the new samples [57]. More detailed descriptions are available in the file in the Supporting Materials.

#### 3.2.4. *E. coli* DNA Gyrase B Suppression Effect

The in vitro enzyme inhibition assessment was performed against *E. coli* DNA gyrase according to the optimized protocol by the manufacturer [58]. More detailed descriptions are available in the file in the Supporting Materials.

#### 3.2.5. In Vitro Cytotoxicity Assay

The cytotoxic effect of test samples using WI38 cells was evaluated by MTT assay [59]. Commercially available kit for in vitro toxicology MTT-based assay from Sigma was used. More detailed descriptions are available in the file in the Supporting Materials.

### 3.3. Molecular Docking

Molecular Operating Environment (MOE, 2019.0102) was used for the docking study. By using the steepest descent technique with the MMFF94x force field until the RMSD gradient of 0.1 kcal.mol^−1^Å^−1^ was reached, energy-minimized structures were created. The crystal structure of the DNA gyrase enzyme complexed with novobiocin was obtained from the protein data bank with PDB ID: 4URO. In the same way, the X-ray crystallographic structure of topoisomerase IV with the co-crystallized ligand novobiocin was downloaded (PDB ID: >1S14). Despite docking software’s capacity to identify potential binding modes between a ligand and its target, it is still an unreliable method that requires constant verification [61,62,63,64,65].

## 4. Conclusions

Based on the molecular hybridization process, new hybrid molecules composed of benzofuran–pyrazole scaffolds are conjugated with different *N*/*O* heterocyclic rings, such as pyridine, pyran, chromene, pyrano-pyrazole, pyrido-triazine, and triazolo-pyridine cores. The newly synthesized target compounds were characterized using microanalysis and spectroscopic approaches. In addition, the new analogues were evaluated as antimicrobial candidates against various strains of both Gram-positive and Gram-negative bacteria, as well as fungi in comparison with novobiocin and clotrimazole as antibacterial and antifungal standard drugs. Promising broad-spectrum antimicrobial potency against the examined bacterial and fungal species was observed with the pyrido-triazine and/or pyrido-triazole derivatives **9**, **10**, and **11b**–**d**, with MIC values ranging from 2.50–20 µg/mL. All the new hybrids were also examined as in vitro antioxidants and anti-inflammatory agents using DPPH-free radical scavenging assays and HRBC membrane stabilization processes, respectively. Compounds **4**, **6**, **9**, **11b**, and **11d** exhibited the most potent scavenging activity (%DPPH scavenging activity = 84.16–90.52%), while all of the examined compounds (except **11c** and **10**) showed significant HRBC membrane stabilization and protection percentages ranging from 86.70 ± 0.259 to 99.25 ± 0.108%. Moreover, the most promising antimicrobial, antioxidant, and anti-inflammatory activities, from compounds **9** and **10**, were evaluated for in vitro suppression effects against *E. coli* DNA gyrase B to find out their expected modes of action as antimicrobial candidates. Compound **9** was a more potent inhibitor against DNA gyrase B than compound **10**, with IC_50_s of 10.71 and 19.58, respectively. Both **9** and **10** revealed promising safety profiles against the normal human diploid cell line WI-38 and were significantly superior to that obtained by the reference drug novobiocin (IC_50_s = 140 ± 0.36, 165 ± 0.40, 163.3 ± 0.17, and 86.2 ± 0.09 µM, respectively).

The hero candidate in this study was the pyrido-triazine derivative **9**, which demonstrated significant broad-spectrum antimicrobial activity with a radical scavenging capacity of 88.56 ± 0.43%, a hemolytic protection rate of 86.70 ± 0.259%, and an *E. coli* DNA gyrase B suppression impact with a promising safety profile. As a result, it could be considered a basic scaffold for the further development of new antimicrobial agents that help overcome the drug resistance phenomenon.

## Figures and Tables

**Table 1:** The antimicrobial potency of the new target benzofuran–pyrazole-based derivatives, expressed as inhibition zone (mm).

Mean Diameter of Zones of Inhibition (Mean ± SEM) (mm)						
Compd. No.	Gram +ve Bacteria		Gram −ve Bacteria		Fungi	
	*S. aureus* *ATCC 6538*	*B. cereus* *ATCC- 11778*	*E. coli* *ATCC-25922*	*P. aeruginosa ATCC-27853*	*F. solani*	*C. albicans* *ATCC-10231*
**2**	15	13	18	20	12	12
**3**	15	14	15	18	12	13
**4**	15	15	15	17	17	15
**5**	15	12	14	20	12	11
**6**	14	12	15	17	0	0
**7**	15	14	15	15	17	16
**8**	20	16	15	17	16	15
**9**	20	18	15	20	16	20
**10**	22	19	15	20	20	20
**11a**	15	12	15	18	15	0
**11b**	20	15	15	20	15	16
**11c**	20	15	17	20	12	15
**11d**	20	15	16	17	15	19
**Novobiocin**	20	20	25	24	-	-
**Clotrimzole**	-	-	-		20	14

Antibacterial standard, Novobiocin (30 µg); antifungal standard, Clotrimazole (50 µg).

**Table 2:** MIC values of the most active analogues against various microbial species (μM).

Compd. No.	Gram +ve Bacteria		Gram −ve bacteria		Fungi	
	*S. aureus* *ATCC 6538*	*B. cereus* *ATCC-11778*	*E. coli* *ATCC-25922*	*P. aeruginosa ATCC-27853*	*F. solani*	*C. albicans* *ATCC-10231*
**4**	15	15	15	17	27	25
**7**	15	14	15	15	27	30
**8**	20	16	15	17	35	25
**9**	2.50	4.65	5.79	17.60	20	16
**10**	3.49	8.80	4.65	20	16	14
**11b**	5.11	8.60	8.69	17.0	15	16
**11c**	5.90	7.79	16.0	16.90	15	19
**11d**	5.11	15	16.0	20	15	19
**Novobiocin**	3.49	6.98	4.65	18.6	-	-
**Clotrimzole**	-	-	-	-	20	14

Standards: novobiocin (30 µg) (antibacterial) and clotrimazole (50 µg) (antifungal).

**Table 3:** In vitro anti-inflammatory and antioxidant activities of the new target benzofuran–pyrazole-based derivatives.

Compound No.	Anti-Inflammatory		Antioxidant Activity
	% Hemolysis	% Protection	% DPPH Radical Scavenging Activity
**2**	0.82 ± 0.108	99.25 ± 0.108	71.19 ± 0.43
**4**	0.67 ± 0.194	99.19 ± 0.194	84.16 ± 4.41
**5**	0.76 ± 0.151	99.13 ± 0.151	58.10 ± 0.52
**6**	0.76 ± 0.086	99.18 ± 0.086	86.42 ± 2.85
**7**	2.38 ± 0.173	97.50 ± 0.173	37.06 ± 1.38
**8**	1.43 ± 0.173	98.44 ± 0.173	7.89 ± 1.04
**9**	13.12 ± 0.259	86.70 ± 0.259	88.56 ± 0.43
**10**	70.68 ± 0.496	29.67 ± 0.496	48.93 ± 0.09
**11a**	1.37 ± 0.086	98.57 ± 0.086	47.16 ± 3.03
**11b**	6.16 ± 0.755	93.30 ± 0.755	89.42 ± 1.56
**11c**	26.60 ± 0.388	73.67 ± 0.388	60.61 ± 2.34
**11d**	2.84 ± 0.259	97.35 ± 0.259	77.00 ± 0.26
**Aspirin**	1.98 ± 0.173	97.90 ± 0.173	-----
**Diclofenac**	0.46 ± 0.043	99.51 ± 0.043	-----
**Ascorbic acid**	----	----	100 ± 0.00

**Table 4:** Assessment of inhibitory potential of compounds 9 and 10 against E. coli DNA gyrase B in relation to novobiocin.

Compound No.	IC_50_ (Mean ± SEM) (µM)	
	*E. coli* DNA Gyrase B	Cytotoxicity WI-38
**9**	9.80 ± 0.21	163.3 ± 0.17
**10**	32.20 ± 0.10	170 ± 0.40
**Ciprofloxacin**	8.03 ± 0.03	86.2 ± 0.03

**Table 5:** Binding energy scores of the synthesized compounds (kcal./mol).

Compound No.	Energy Score (kcal/mol.)	Compound No.	Energy Score (kcal/mol.)
**4**	−8.2	**10**	−8.7
**5**	−8.4	**11a**	−8.4
**6**	−8.6	**11b**	−8.1
**7**	−8.1	**11c**	−7.9
**8**	−8.3	**11d**	−8.2
**9**	−8.9	**Novobiocin**	−9.4

**Table 6:** Prediction of physicochemical and AMDE properties of 4–11 employing SwissADME.

Compound No.	Molecular Weight	Rotatable Bonds	H-bond Acceptors	H-bond Donors	Molar Refractivity	Topological Polar Surface Area (TPSA) TPSA	Log *P*	GI Absorption	BBB Permeant	PGP Substrate	Lipinski # Violations	Bioavailability Score	Synthetic Accessibility
**4**	433.42	3	5	2	122.44	152.58 Å^2^	2.33	Low	No	Yes	0	0.55	3.7
**5**	466.49	6	6	1	128.77	116.30 Å^2^	3.78	Low	No	No	0	0.56	4.61
**6**	446.46	3	5	2	126.22	110.23 Å^2^	3.92	High	No	Yes	0	0.55	4.44
**7**	476.53	3	5	1	134.92	107.07 Å^2^	4.24	Low	No	No	0	0.56	4.78
**8**	434.45	3	5	2	121.31	118.68 Å^2^	3.44	High	No	Yes	0	0.55	4.44
**9**	459.46	3	5	2	136	124.60 Å^2^	2.67	High	No	Yes	0	0.55	3.96
**10**	499.48	4	7	0	138.1	134.91 Å^2^	3.14	Low	No	Yes	0	0.55	3.92
**11a**	611.61	7	8	2	175.16	152.29 Å^2^	3.69	Low	No	Yes	2	0.17	5.12
**11b**	555.97	4	5	2	160.69	124.60 Å^2^	4.27	Low	No	No	1	0.55	4.66
**11c**	525.52	4	6	2	152.92	137.74 Å^2^	3.51	Low	No	Yes	1	0.55	4.72
**11d**	527.56	4	5	2	153.56	152.84 Å^2^	3.81	Low	No	Yes	1	0.55	4.60

## Data Availability

The original contributions presented in the study are included in the article/Supplementary Materials, further inquiries can be directed to the corresponding author.

## Supplementary Materials

The following supporting information can be downloaded at: https://www.mdpi.com/article/10.3390/ph17121664/s1, NMR Spectra of benzofuran-pyrazole based compounds (Figures S1–S24) and biological experimental part.
