# Supplementary material for: New Benzofuran–Pyrazole-Based Compounds as Promising Antimicrobial Agents: Design, Synthesis, DNA Gyrase B Inhibition, and In Silico Studies
Source: Pharmaceuticals (Basel). 2024 Dec 10;17(12):1664. doi: 10.3390/ph17121664 (PMC11676098; doi:10.3390/ph17121664)
Supplement: Supplementary file 1 [file pharmaceuticals-17-01664-s001.zip › pharmaceuticals-3295578-supplementary.pdf]

Supporting Information

New Benzofuran-Pyrazole Based Compounds as Promising Antimicrobial Agents: Design, Synthesis, DNA Gyrase B Inhibition and *In Silico* Studies

Somaia S. Abd El-Karim[1], Manal M. Anwar[1], Yasmin M. Syam[1], Hassan M. Awad[2], Asmaa Negm El-Dein[2], Mohamed K. El-Ashrey[3,4], Hamad M. Alkahtani[5] and Sameh H.Abdelwahed[6,7]*

[1]Department of Therapeutic Chemistry, Pharmaceutical and Drug Industries Research Institute, National Research Centre, Dokki, Cairo, P.O. 12622, Egypt
[2] Chemistry of Natural and Microbial Products Department, Pharmaceutical and Drug Industries Research Institute, National Research Centre, Dokki, Cairo, P.O. 12622, Egypt
[3] Pharmaceutical Chemistry Department, Faculty of Pharmacy, Cairo University, Kasr Elini St., Cairo 11562, Egypt
[4] Medicinal Chemistry Department, Faculty of Pharmacy, King Salman International University (KSIU), South Sinai 46612, Egypt
5Department of Pharmaceutical Chemistry, College of Pharmacy, King Saud University, P.O. Box 2457, Riyadh 11451, Saudi Arabia
[6]Department of Chemistry, Prairie View A&M University, Prairie View, TX 77446, USA
[7]Department of Chemistry, Texas A&M University, College Station, TX 77843, USA
*Correspondence: shabdelwahed@pvamu.edu

	Contents	Page
1	**5. Experimental protocols**<br>*5.1. Chemistry*	2
2	*NMR Spectra of benzofuran-pyrazole based compounds (Figures S1-S24)*	3
3	*5.2.1. In vitro antimicrobial activity*	27
4	*5.2.1.1 Microorganisms*	27
5	*5.2.1.2. Inoculum Preparation*	27
6	*5.2.1.3. Bioassay method*	27
7	*5.2.2. DPPH radical scavenging assay*	28
8	*5.2.3. Human red blood cell stabilization method*	28

9	***5.2.4. Enzyme assessment of E.coli DNA Gyrase***	**29**
10	***5.2.5. In vitro*** **cytotoxicity assay**	**29**
11	***5.2.6. E. coli DNA gyrase assay (Figures S25-S27)***	**30**
12	***5.3. ADMET studies (Figure S28)***	**34**

5. Experimental protocols

5.1. Chemistry

All melting points are uncorrected and were taken in open capillary tubes using Electrothermal apparatus 9100. Elemental microanalyses were carried out at Microanalytical Unit, Central Services Laboratory, National Research Centre, Dokki, Cairo, Egypt, using Vario Elementar and were found within ±0.4% of the theoretical values. Infrared spectra were recorded on a FT/IR-4100 Jasco-Japan, Fourier transform, Infrared spectrometer at cm^{-1} scale using KBr disc technique at Central Services Laboratory, National Research Centre, Dokki, Cairo, Egypt. ^{1}H NMR and ^{13}C NMR spectra were determined by using a Varian Mercury Plus 300 MHz Spectrometer at Ministry of defense, Chemical Warfare Department, Cairo, Egypt and Bruker High Performance Digital FT-NMR Spectrometer Avance III 500MHz, National Research Center, Cairo, Egypt. Chemical shifts are expressed in δ (ppm) downfield from TMS as an internal standard. The mass spectra were measured with a GC MS-Qp1000EX Shimadzu, Cairo University, Cairo, Egypt. Follow up of the reactions and checking the purity of the compounds were made by TLC on silica gel-precoated aluminium sheets (Type 60, F 254, Merck, Darmstadt, Germany) using chloroform/methanol (20:1, v/v) and the spots were detected by exposure to UV lamp at λ_{254} nanometer for few seconds and

by iodine vapor. The chemical names given for the prepared compounds are according to the IUPAC system.

N′-((3-(benzofuran-2-yl)-1-phenyl-1H-pyrazol-4-yl)methylene)-2-cyanoacetohydrazide (3)

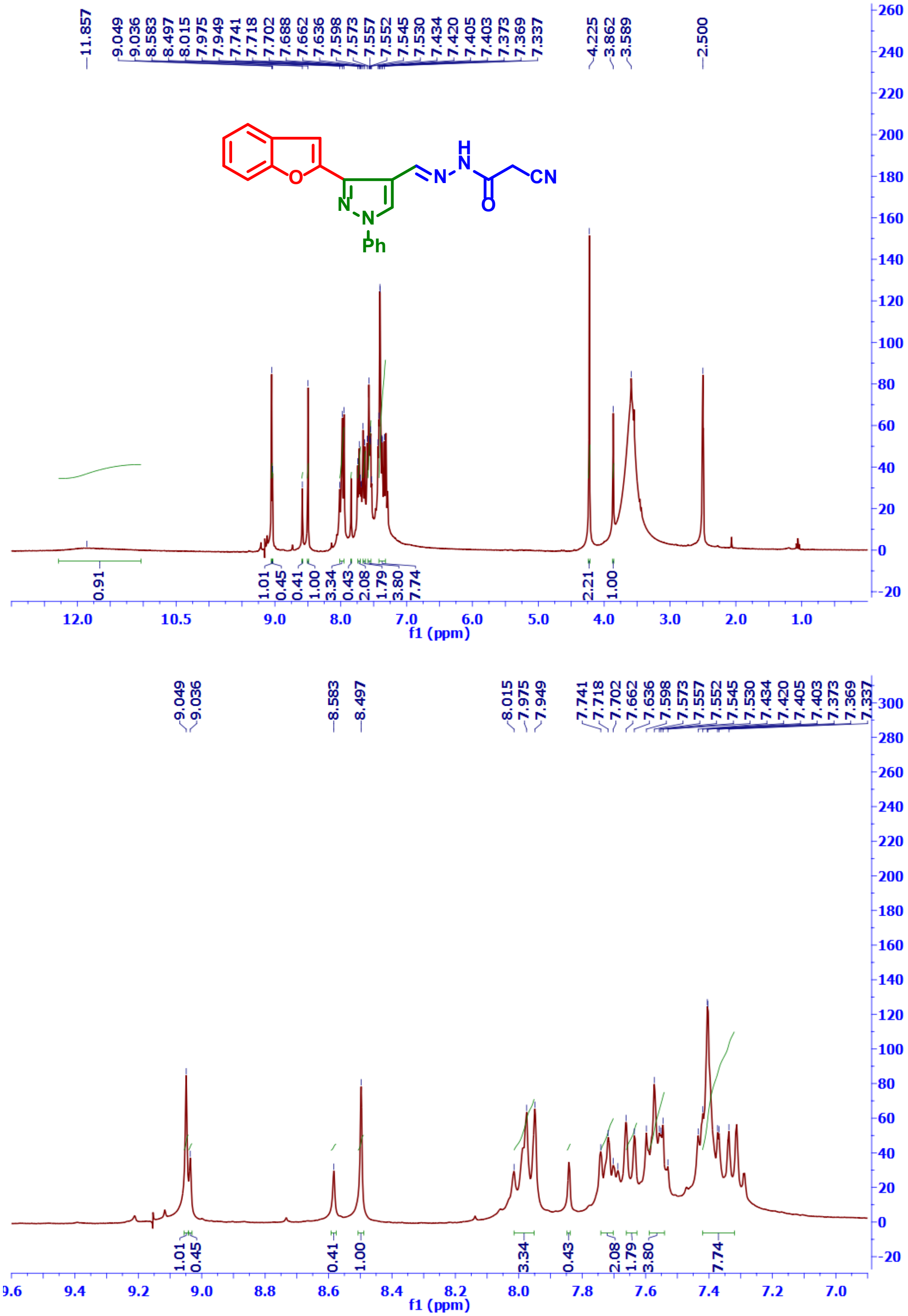

Figure S1: ^{1}H (500 MHz) NMR spectra of **3** in DMSO-d_6

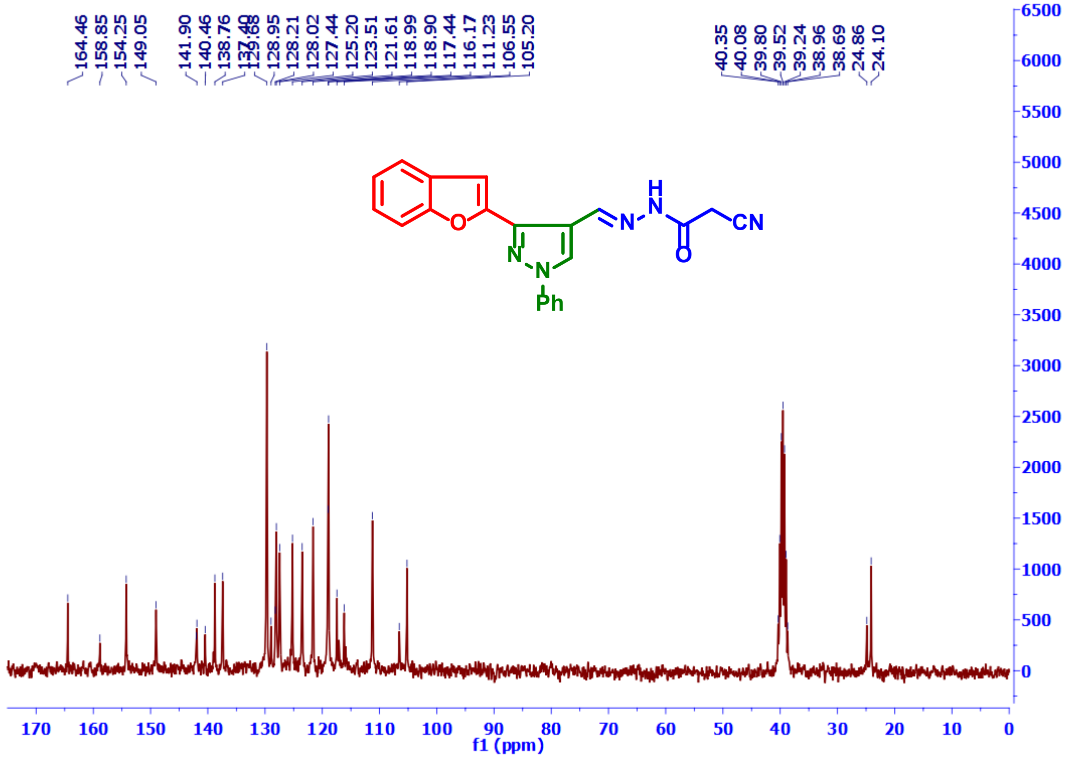

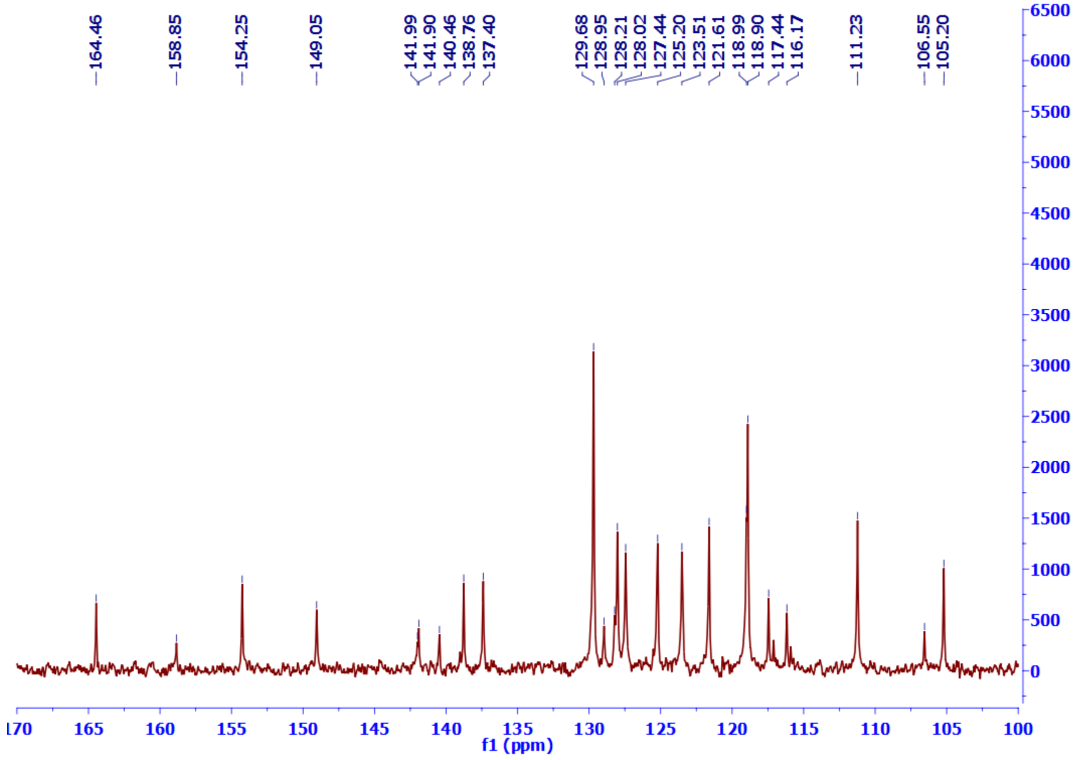

Figure S2: ^{13}C (125 MHz) NMR spectra of **3** in DMSO-d_6

1,6-Diamino-4-(3-(benzofuran-2-yl)-1-phenyl-1H-pyrazol-4-yl)-1,2-dihydro-2-oxopyridine-3,5-dicarbonitrile (4)

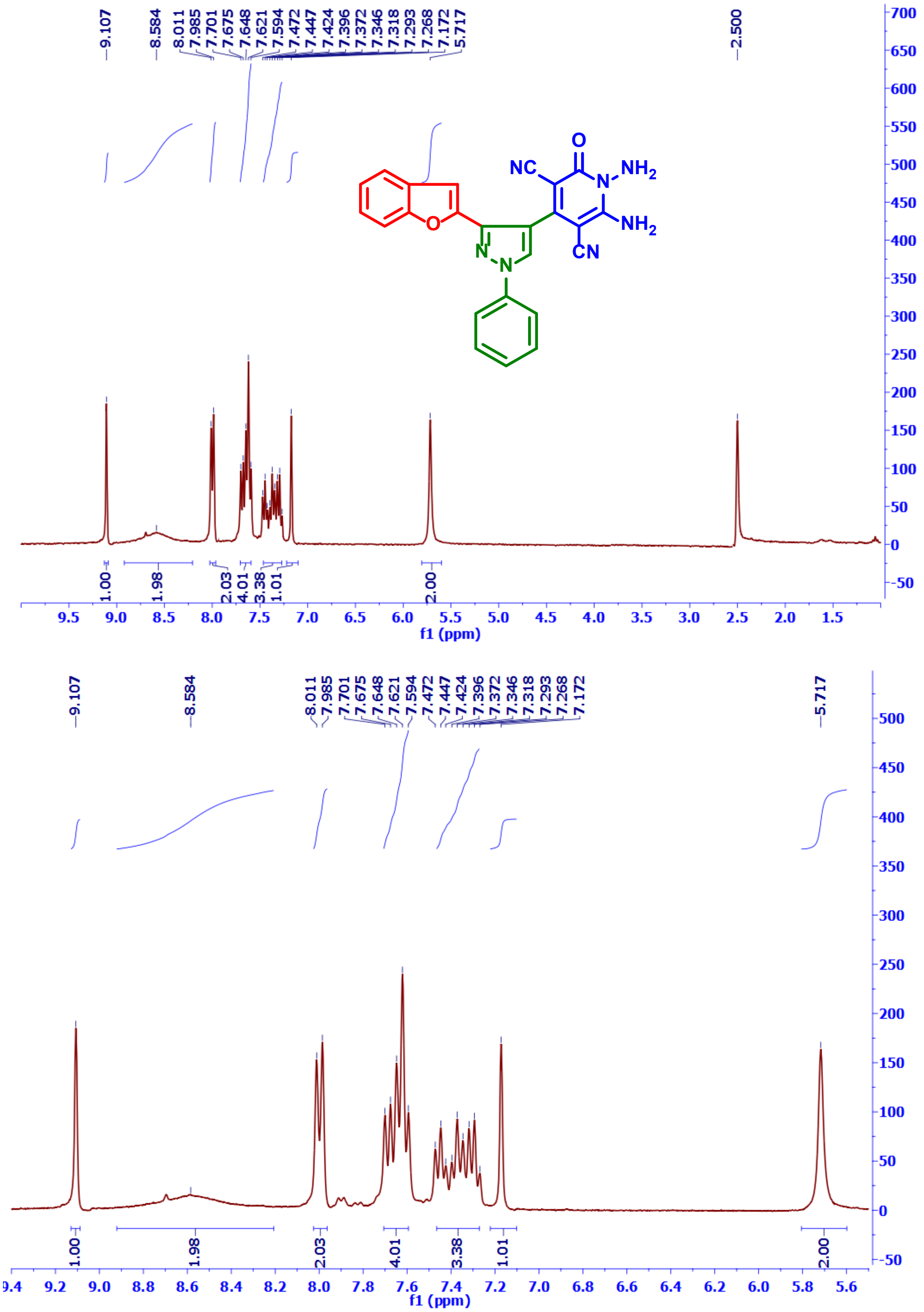

Figure S3: ^{1}H (300 MHz) NMR spectra of **4** in DMSO-d_6

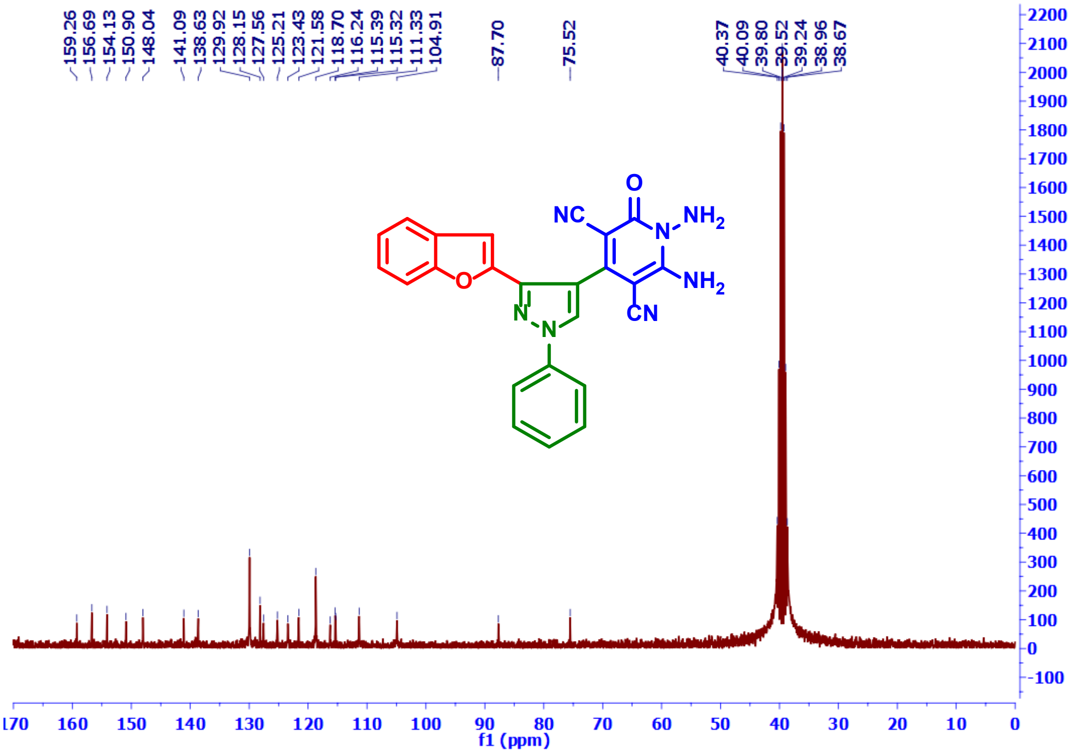

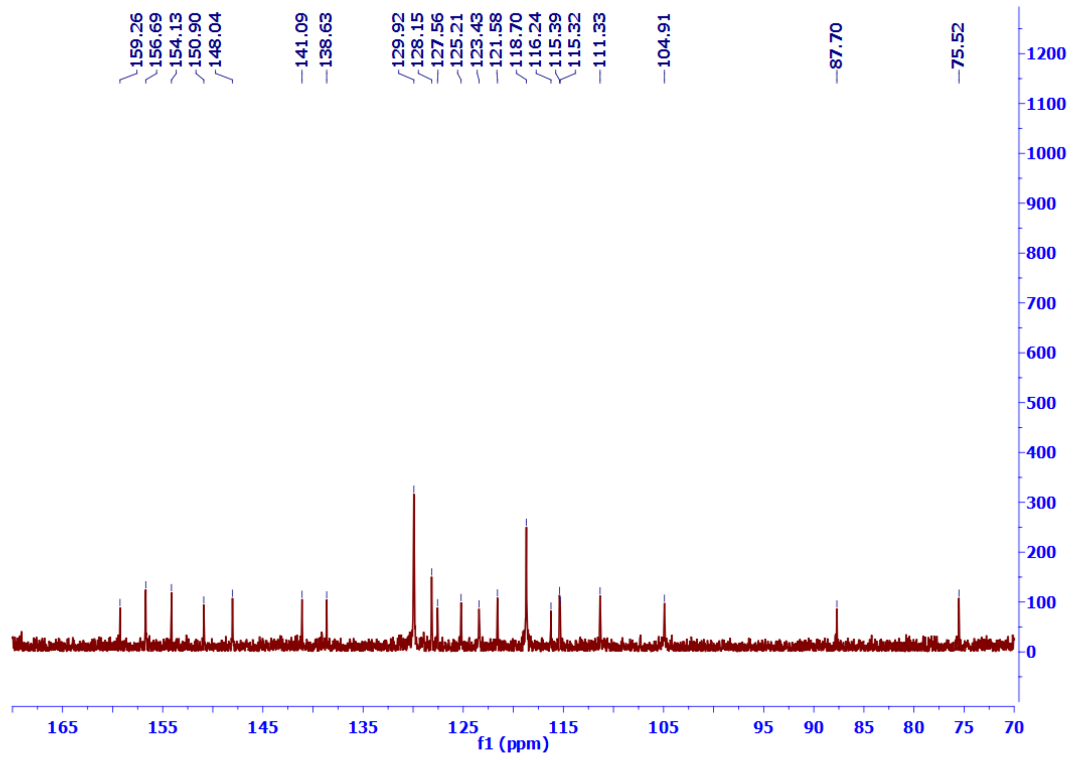

Figure S4: ^{13}C (75 MHz) NMR spectra of **4** in DMSO-d_6

Ethyl 6-amino-4-(3-(benzofuran-2-yl)-1-phenyl-1H-pyrazol-4-yl)-5-cyano-2-methyl-4H-pyran-3-carboxylate (*5*)

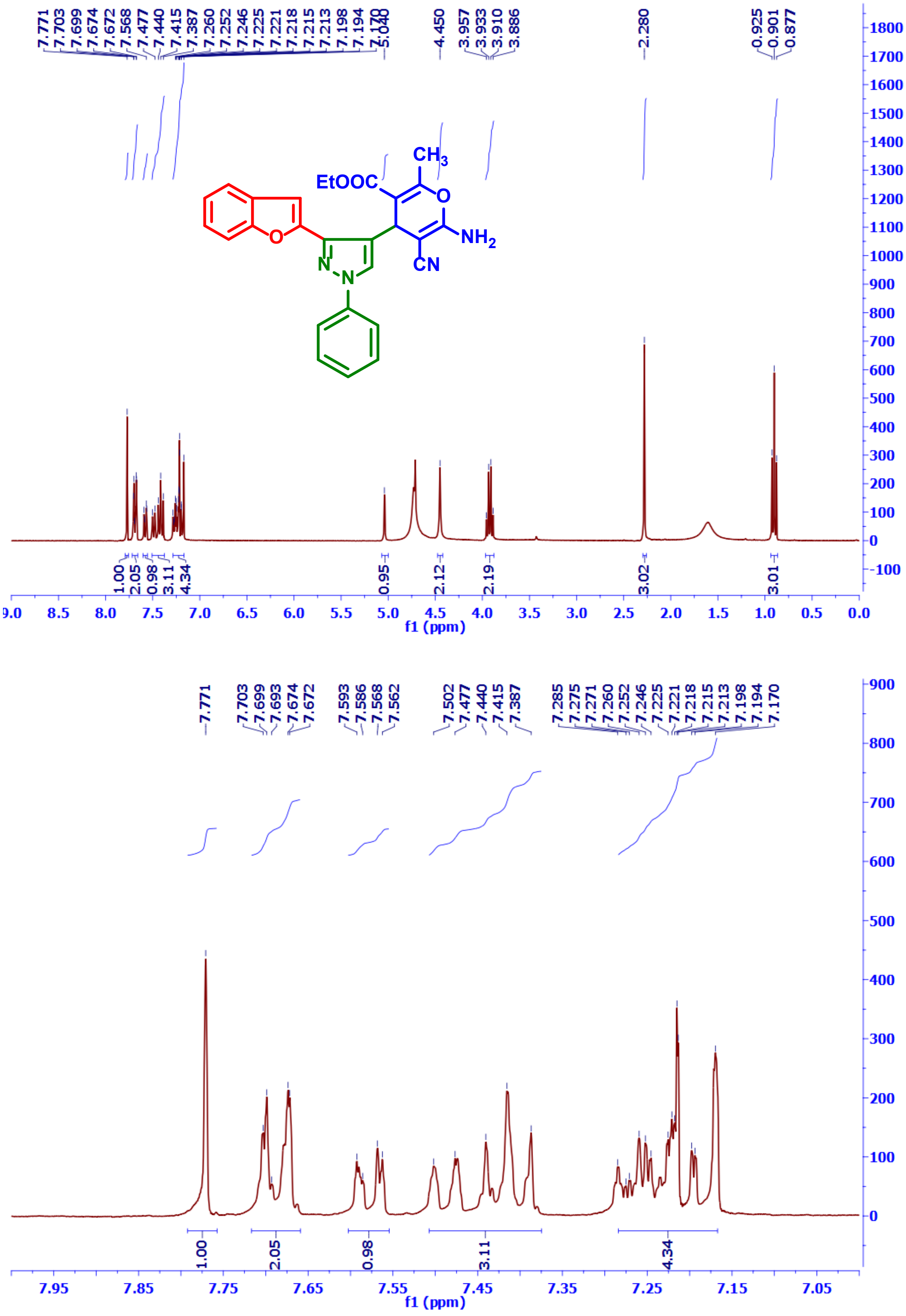

Figure S5: ^{1}H (300 MHz) NMR spectra of **5** in $CDCl_3$

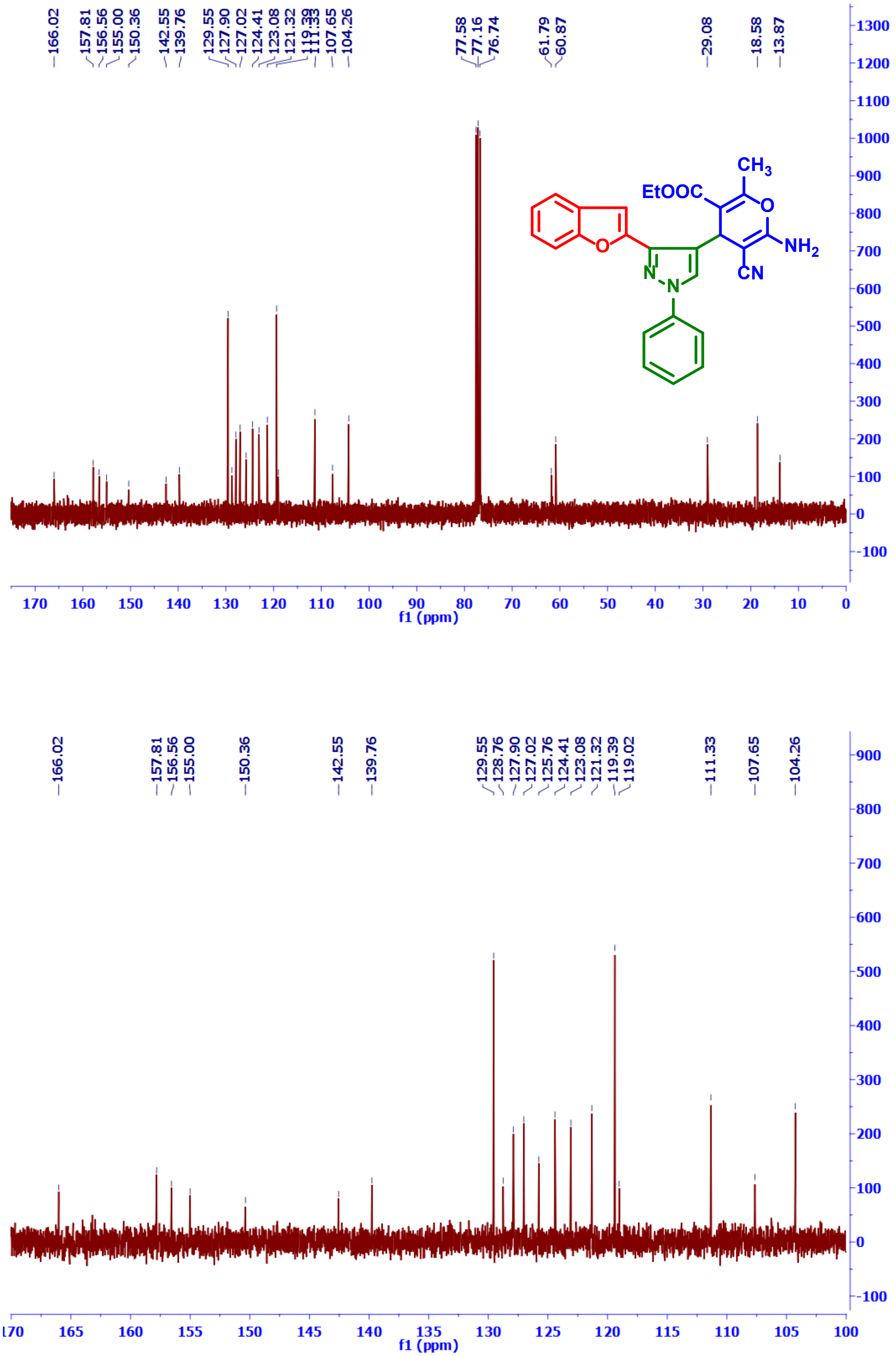

Figure S6: ^{13}C (75 MHz) NMR spectra of **5** in $CDCl_3$

2-Amino-4-(3-(benzofuran-2-yl)-1-phenyl-1H-pyrazol-4-yl)-7-hydroxy-4H-chromene-3-carbonitrile (6)

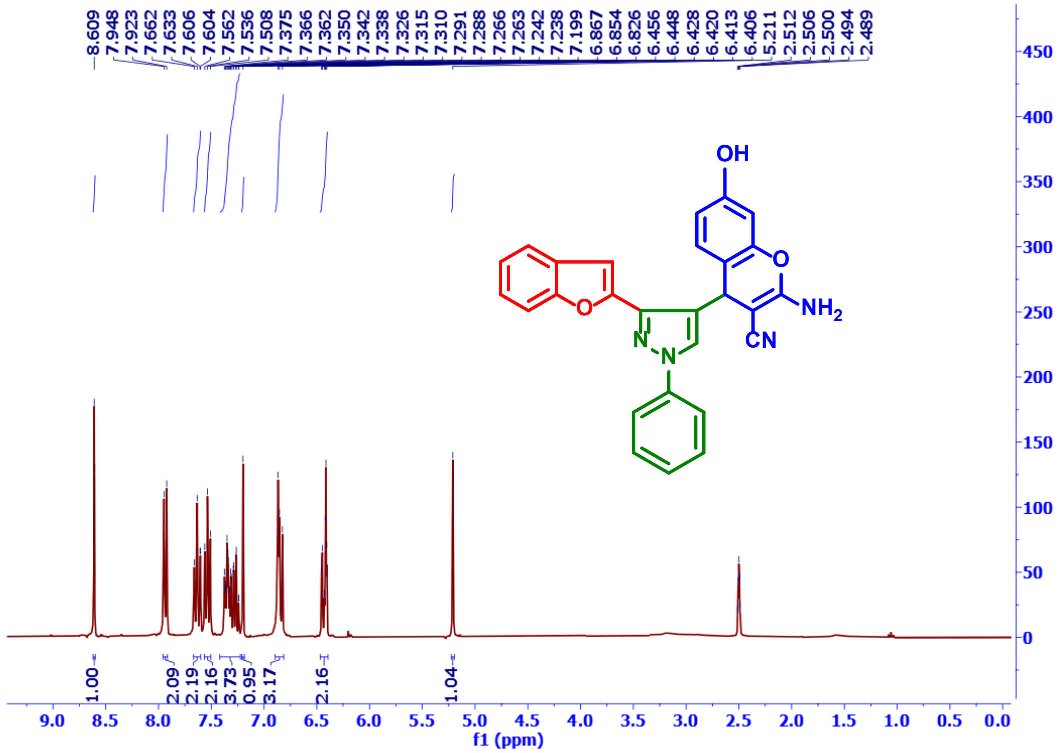

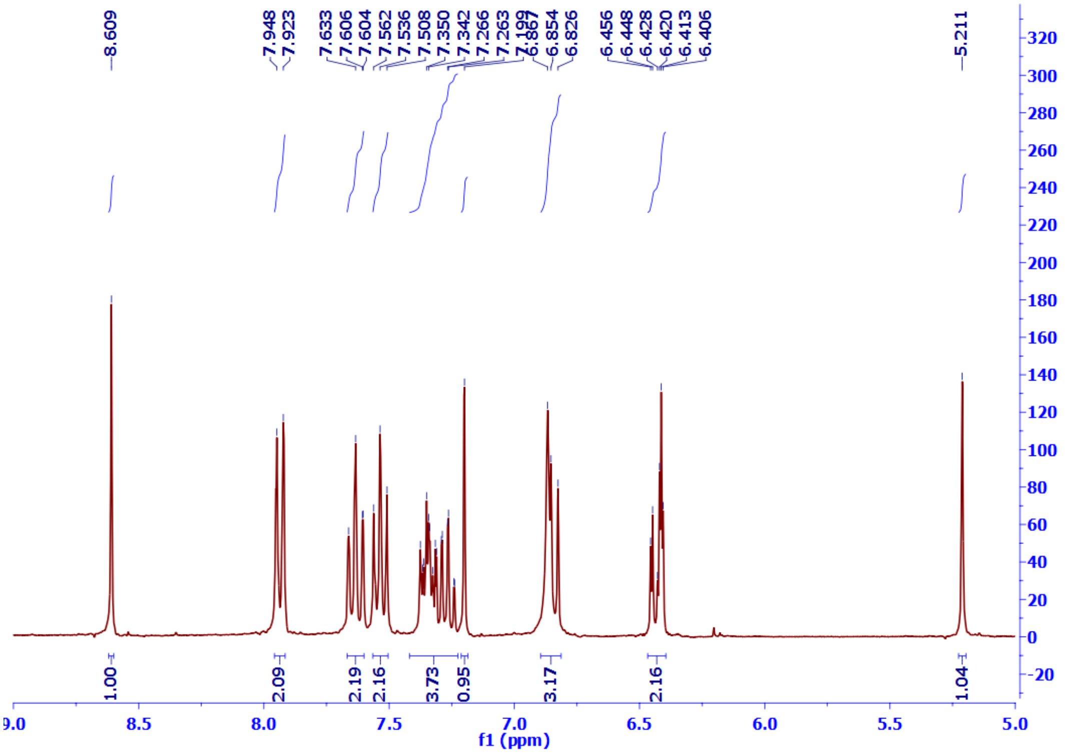

Figure S7: ^{1}H (300 MHz) NMR spectra of **6** in DMSO-d_6

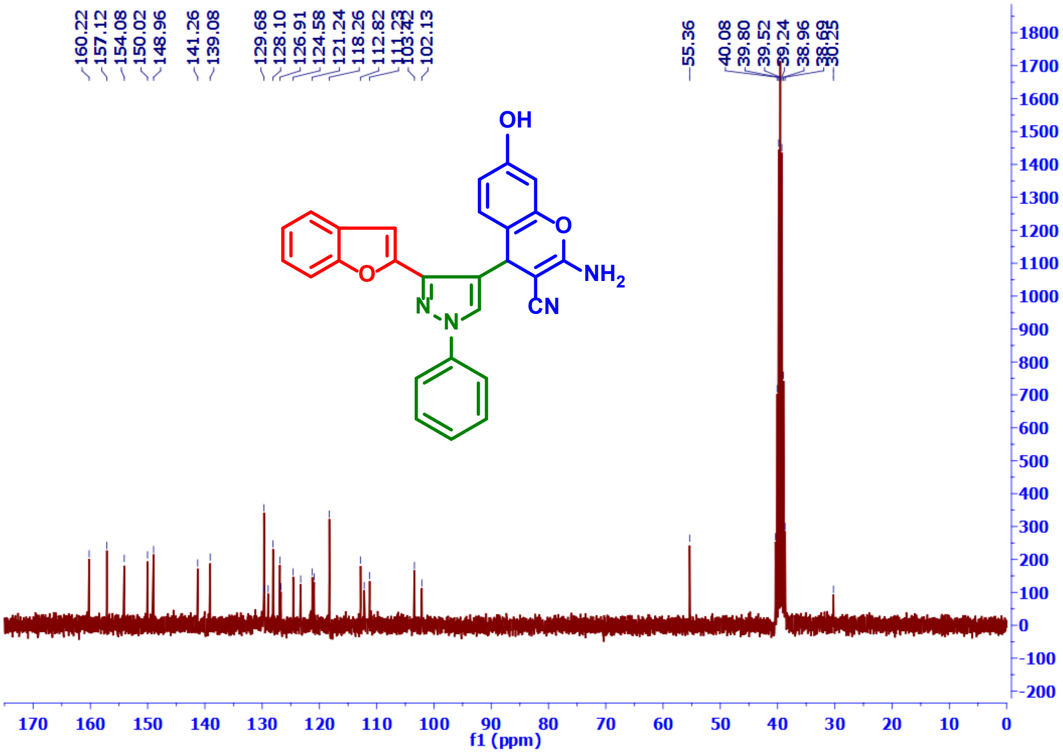

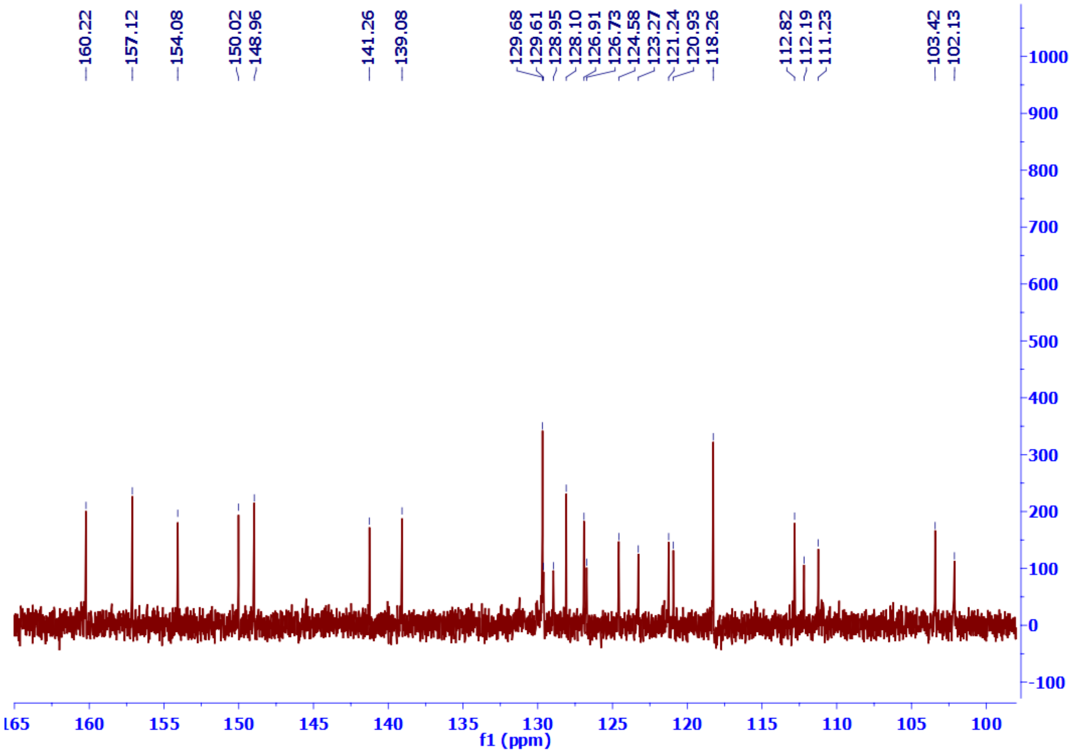

Figure S8: ^{13}C (75 MHz) NMR spectra of **6** in DMSO-*d*$_6$

2-Amino-4-(3-(benzofuran-2-yl)-1-phenyl-1H-pyrazol-4-yl)-5,6,7,8-tetrahydro-7,7-dimethyl-5-oxo-4H-chromene-3-carbonitrile (7)

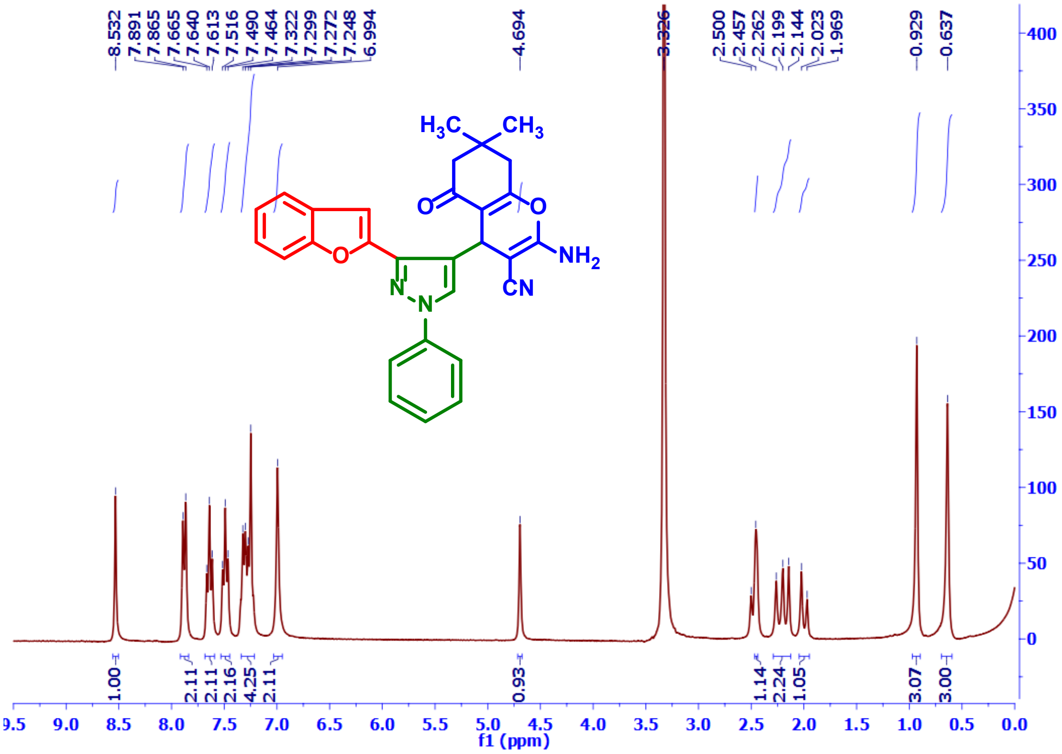

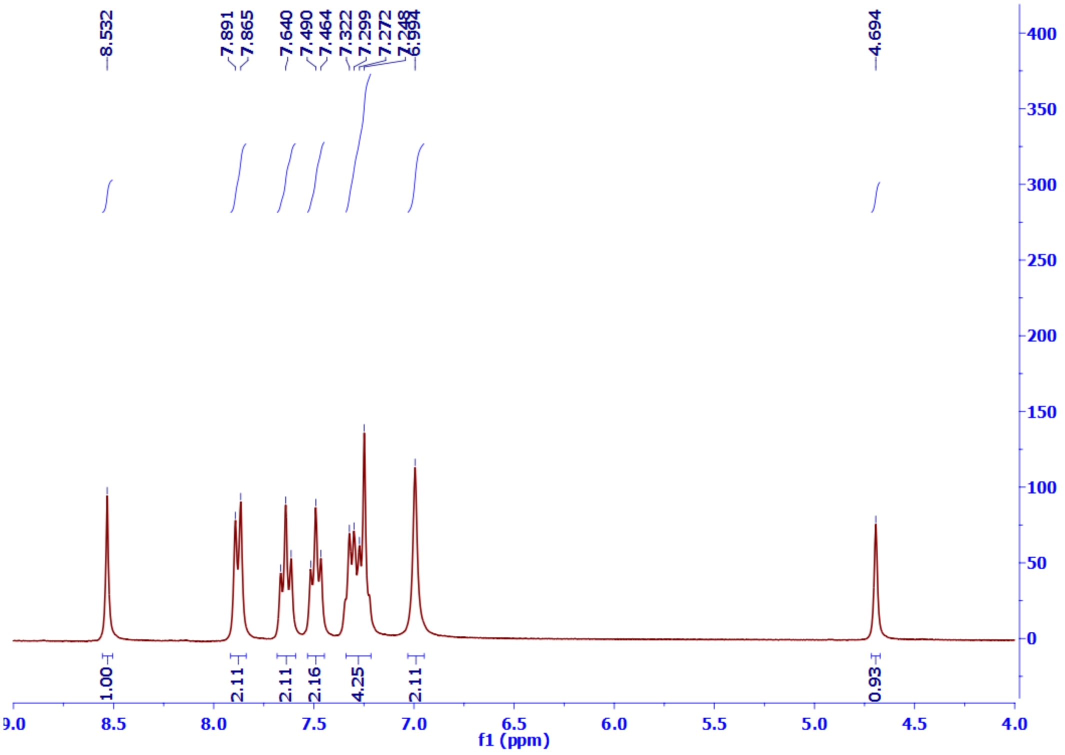

Figure S9: ^{1}H (300 MHz) NMR spectra of **7** in DMSO-d_6

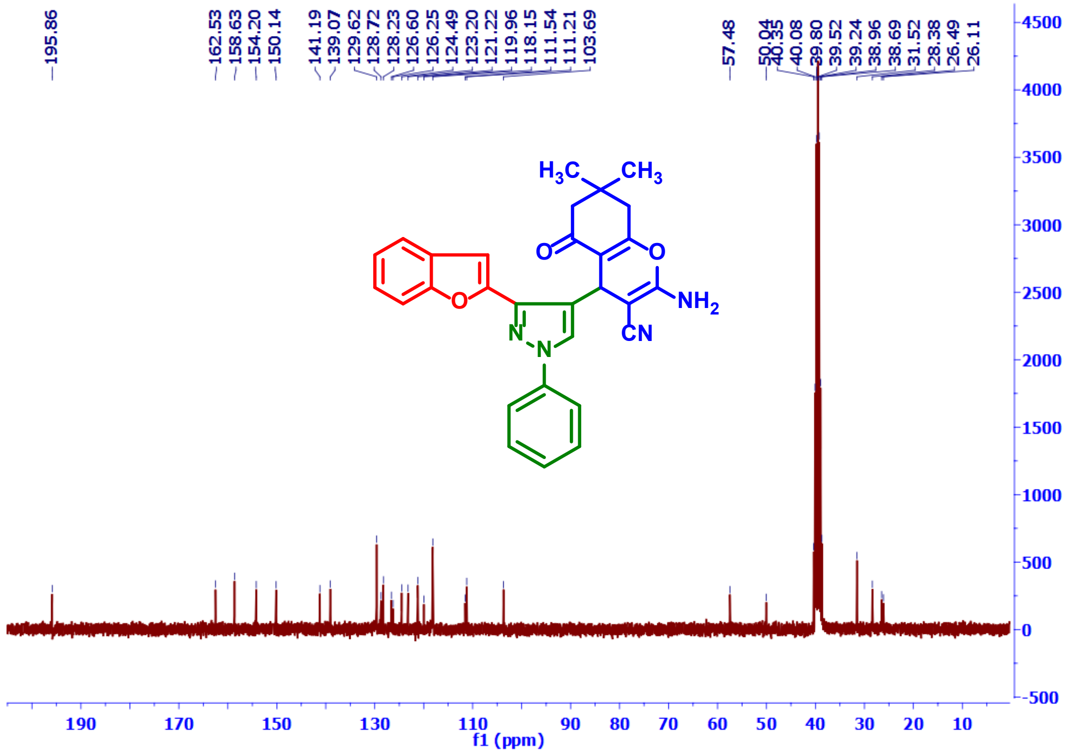

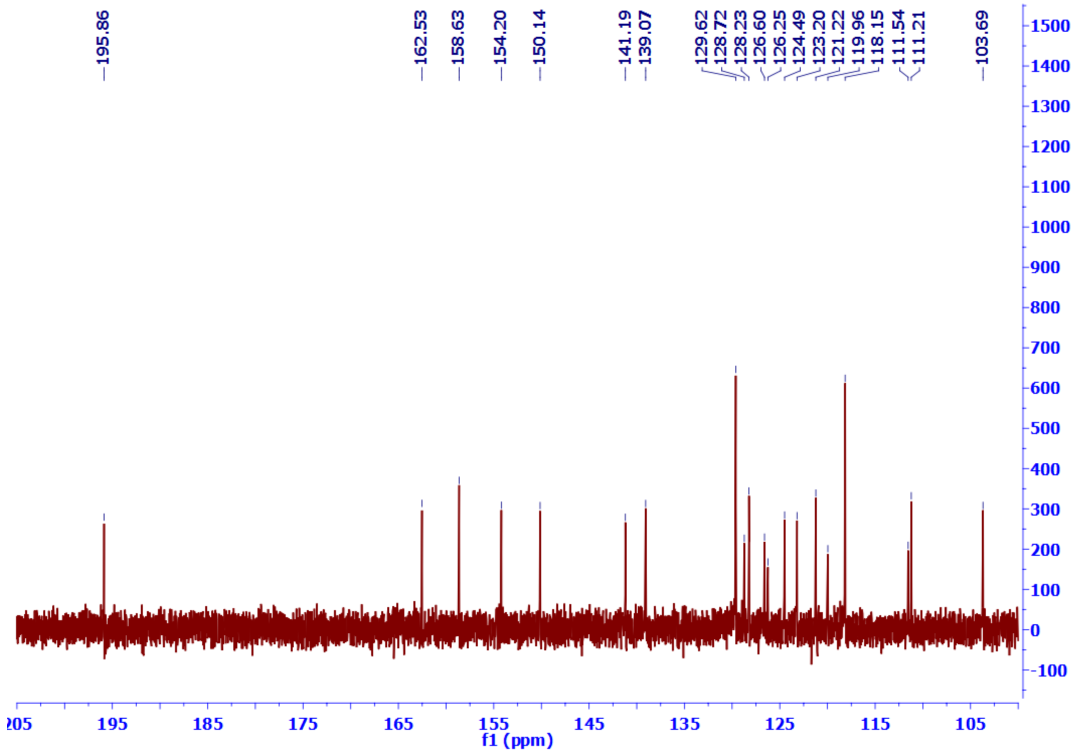

Figure S10: ^{13}C (75 MHz) NMR spectra of **7** in DMSO-d_6

6-Amino-4-(3-(benzofuran-2-yl)-1-phenyl-1H-pyrazol-4-yl)-3-methyl-1,4-dihydropyrano[2,3-c]pyrazole-5-carbonitrile (8)

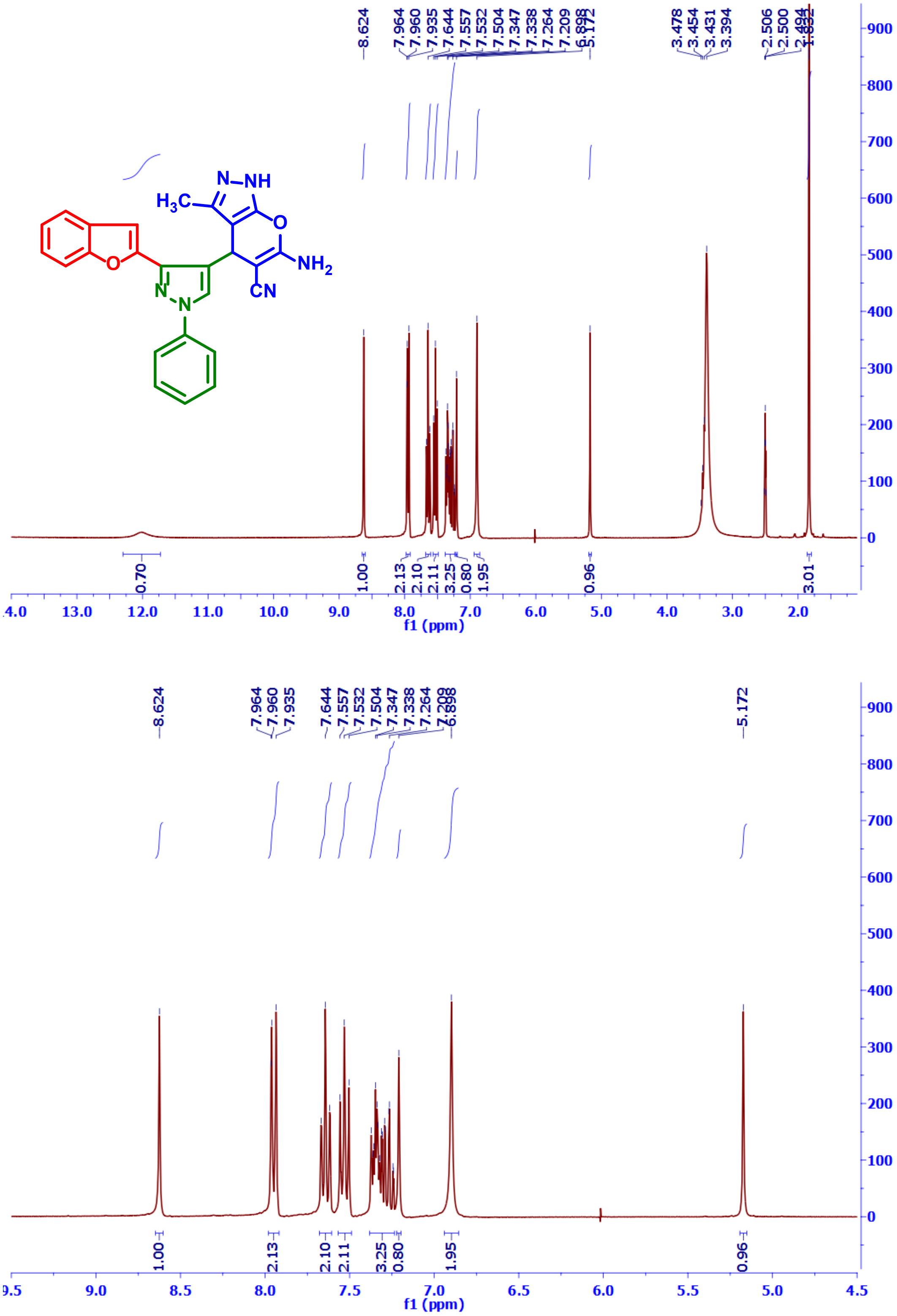

Figure S11: ^{1}H (300 MHz) NMR spectra of **8** in DMSO-*d*$_6$

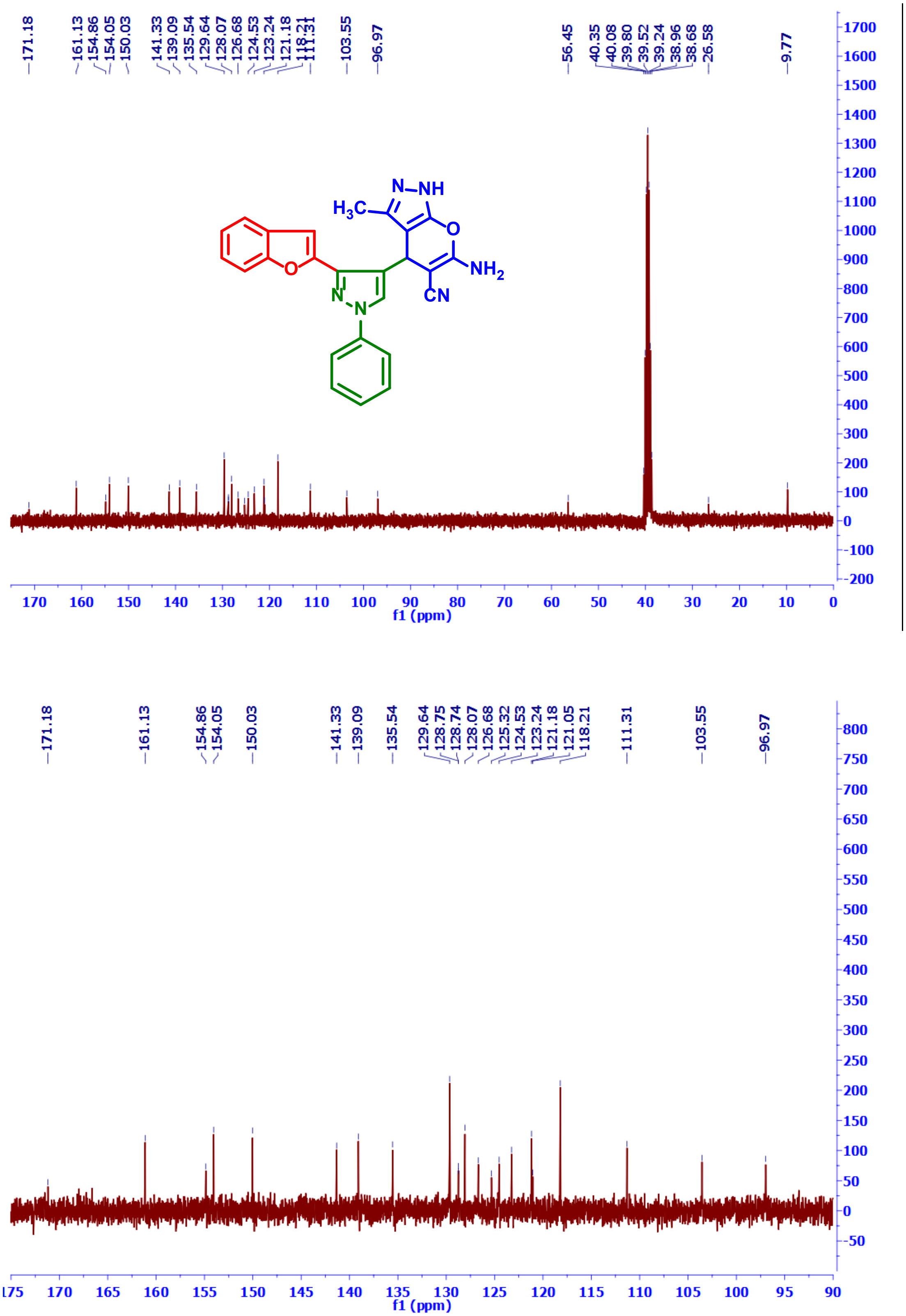

Figure S12: ^{13}C (75 MHz) NMR spectra of **8** in DMSO-d_6

***8-(3-(Benzofuran-2-yl)-1-phenyl-1H-pyrazol-4-yl)-6-oxo-1,3,4,6-tetrahydro-2H-pyrido[1,2-b][1,2,4]triazine-7,9-dicarbonitrile* (9)**

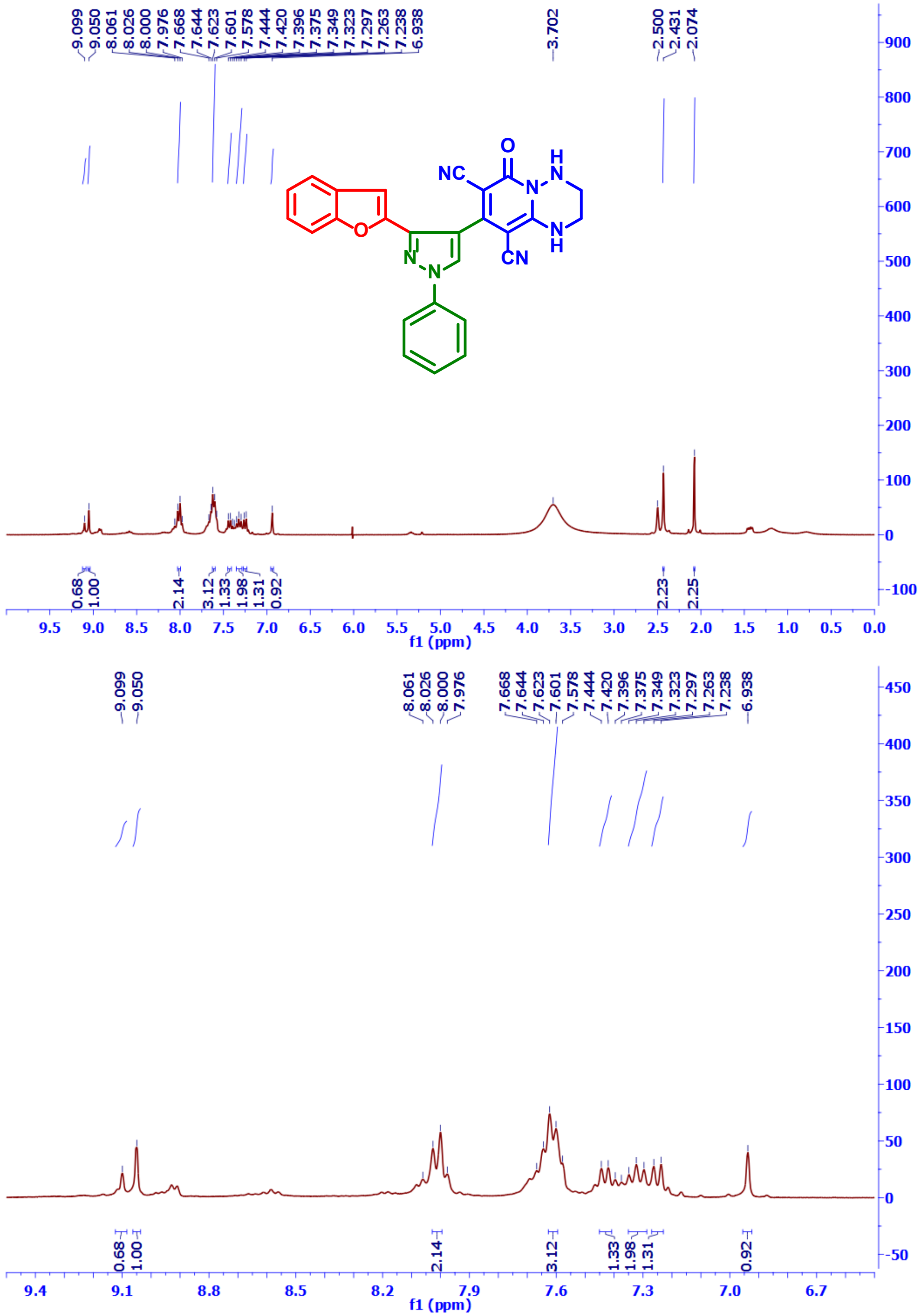

Figure S13: ^{1}H (300 MHz) NMR spectra of **8** in DMSO-d_6

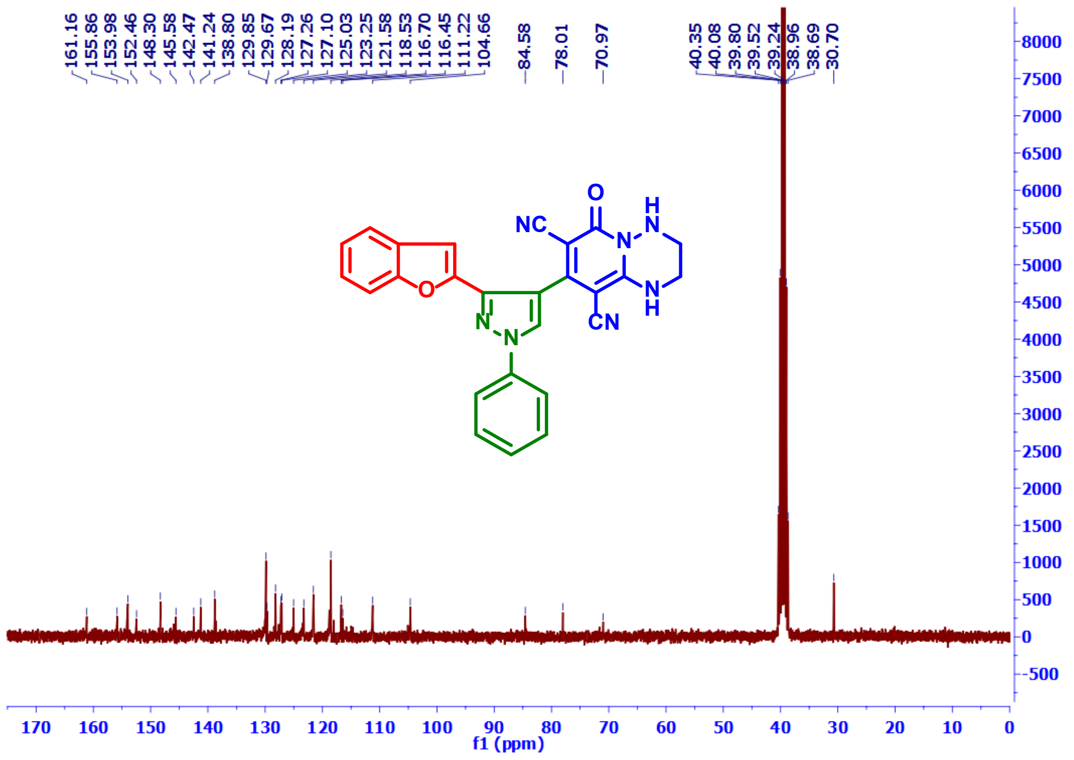
161.16
155.86
153.98
152.46
148.30
145.58
142.47
141.24
138.80
129.85
129.67
128.19
127.26
127.10
125.03
123.25
121.58
118.53
116.70
116.45
111.22
104.66
84.58
78.01
70.97
40.35
40.08
39.80
39.52
39.24
38.96
38.69
30.70
NC
O
H
N
N
N
H
CN
O
N
N
f1 (ppm)

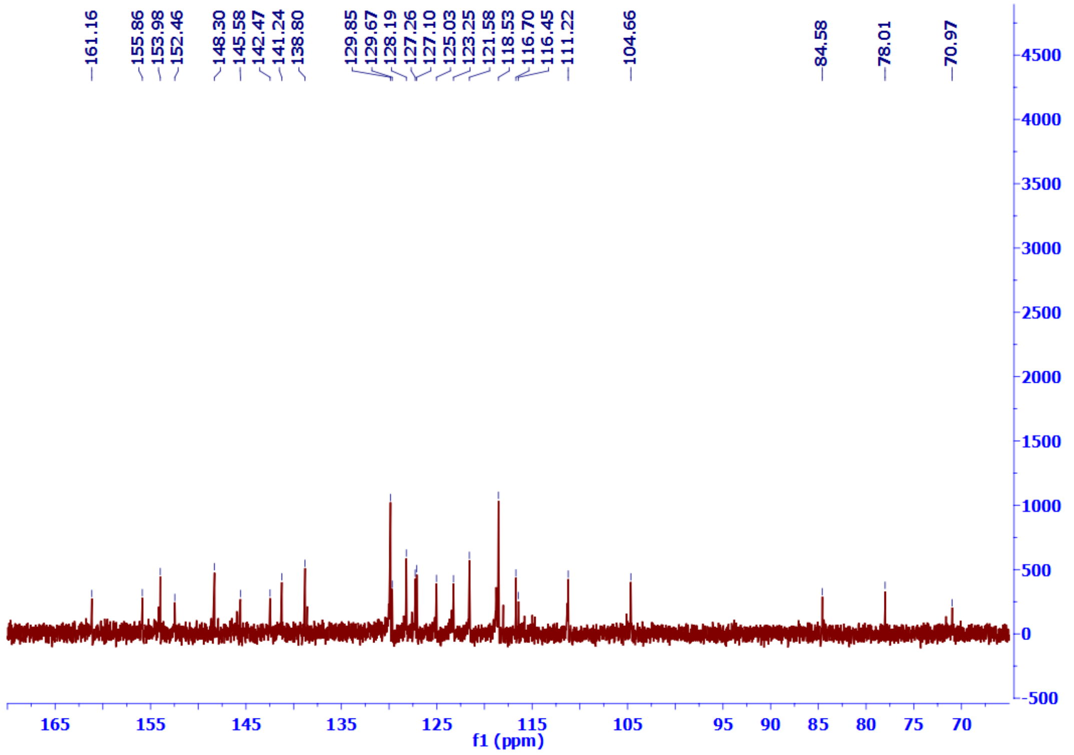
161.16
155.86
153.98
152.46
148.30
145.58
142.47
141.24
138.80
129.85
129.67
128.19
127.26
127.10
125.03
123.25
121.58
118.53
116.70
116.45
111.22
104.66
84.58
78.01
70.97
f1 (ppm)

Figure S14: ^{13}C (75 MHz) NMR spectra of **9** in DMSO-d_6

3-Acetyl-7-(3-(benzofuran-2-yl)-1-phenyl-1H-pyrazol-4-yl)-3,5-dihydro-2-methyl-5-oxo-[1,2,4]triazolo[1,5-a]pyridine-6,8-dicarbonitrile (10)

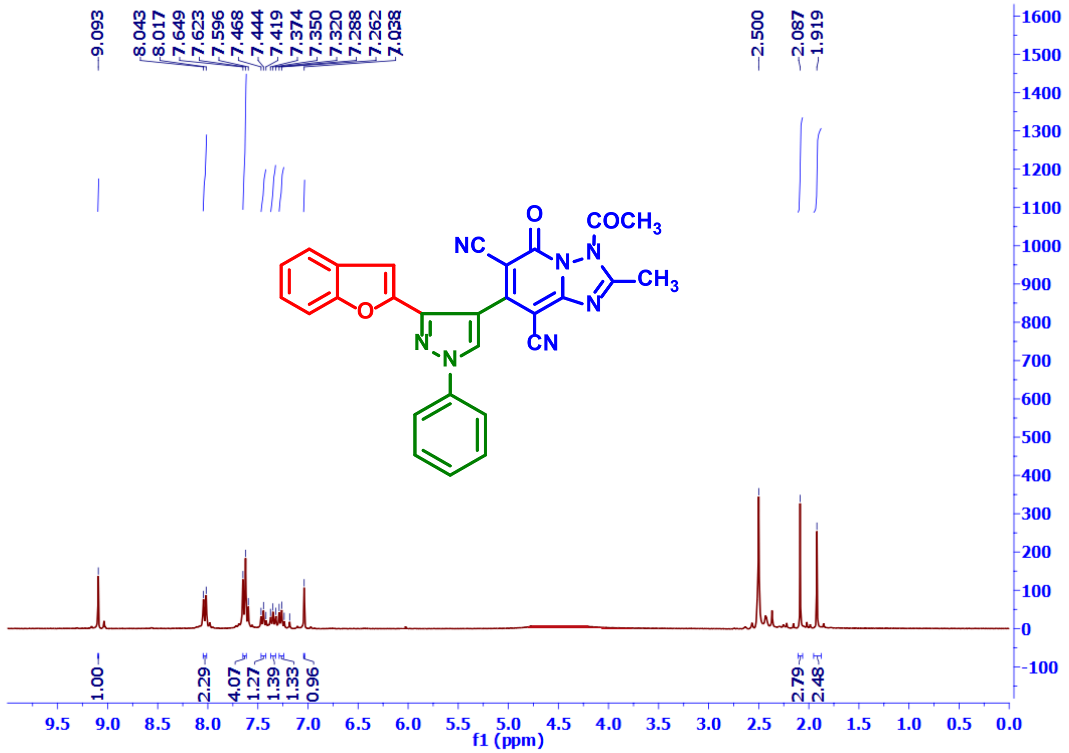

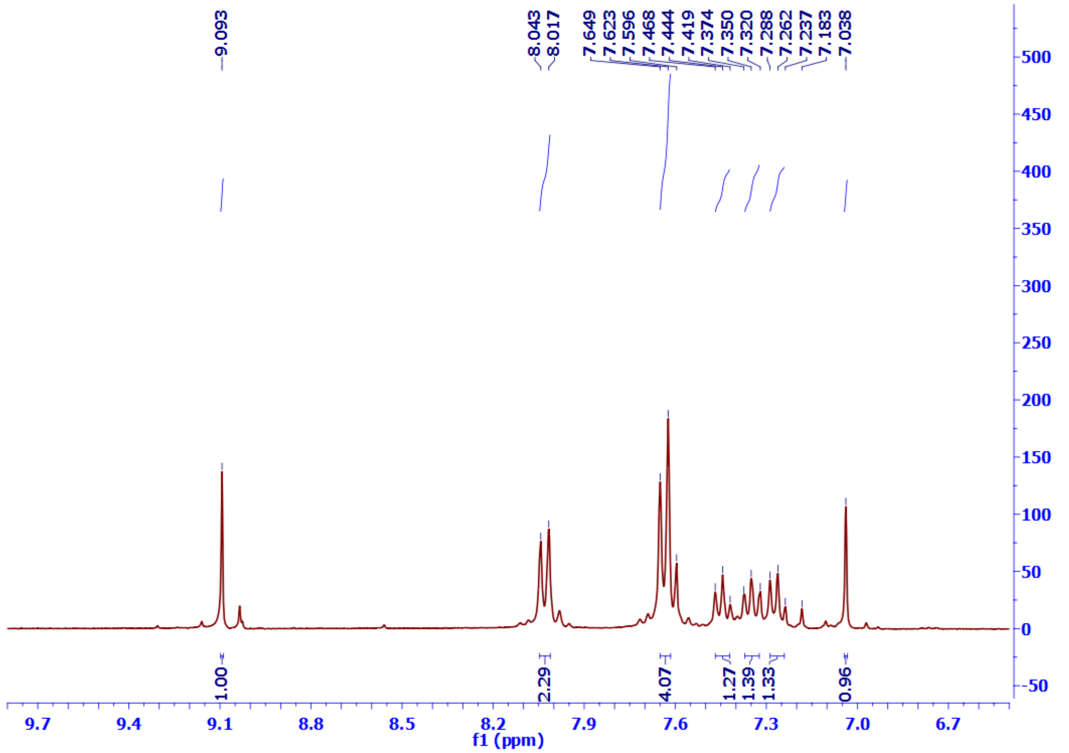

Figure S15: ^{1}H (300 MHz) NMR spectra of **10** in DMSO-d_6

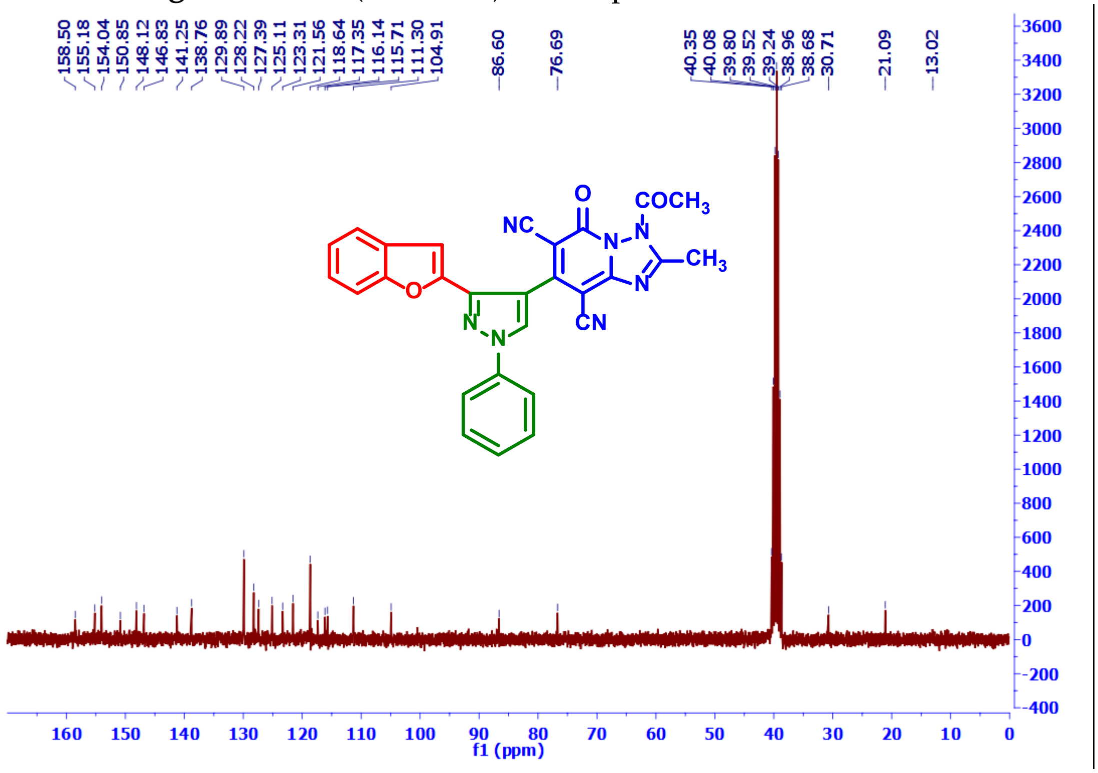

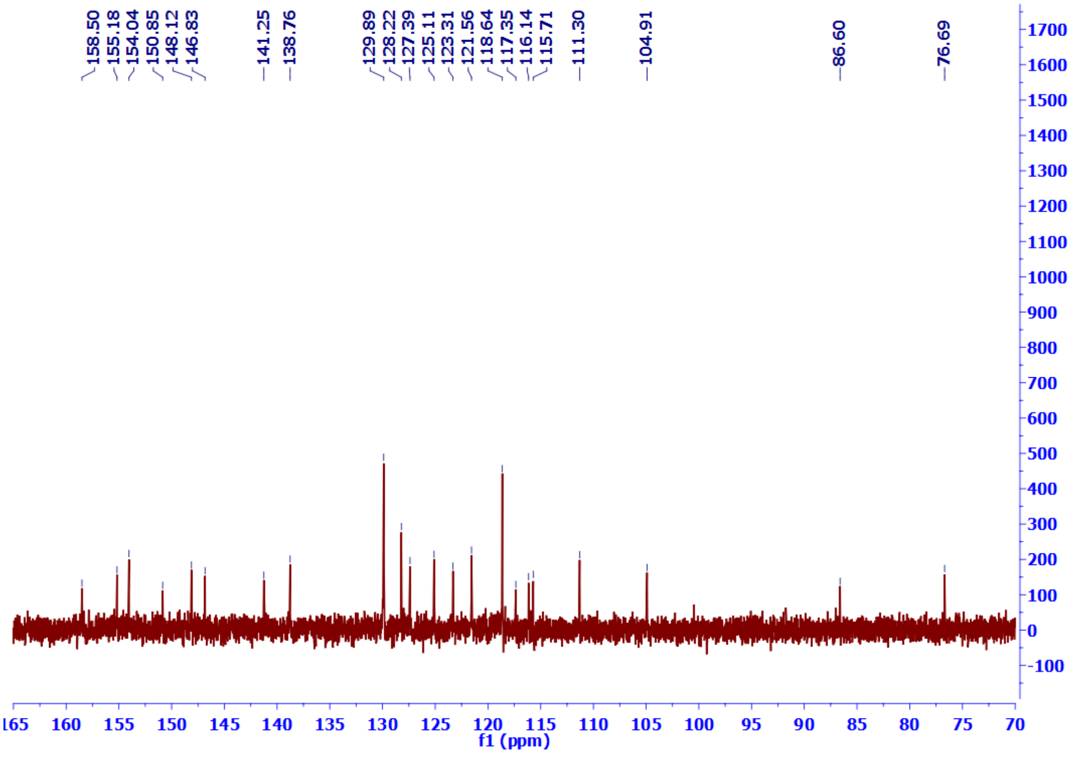

Figure S16: ^{13}C (75 MHz) NMR spectra of **10** in DMSO-d_6

7-(3-(Benzofuran-2-yl)-1-phenyl-1H-pyrazol-4-yl)-1,2,3,5-tetrahydro-2- (3,4,5-trimethoxyphenyl)-5-oxo-[1,2,4]triazolo[1,5-a]pyridine-6,8-dicarbonitrile (11a)

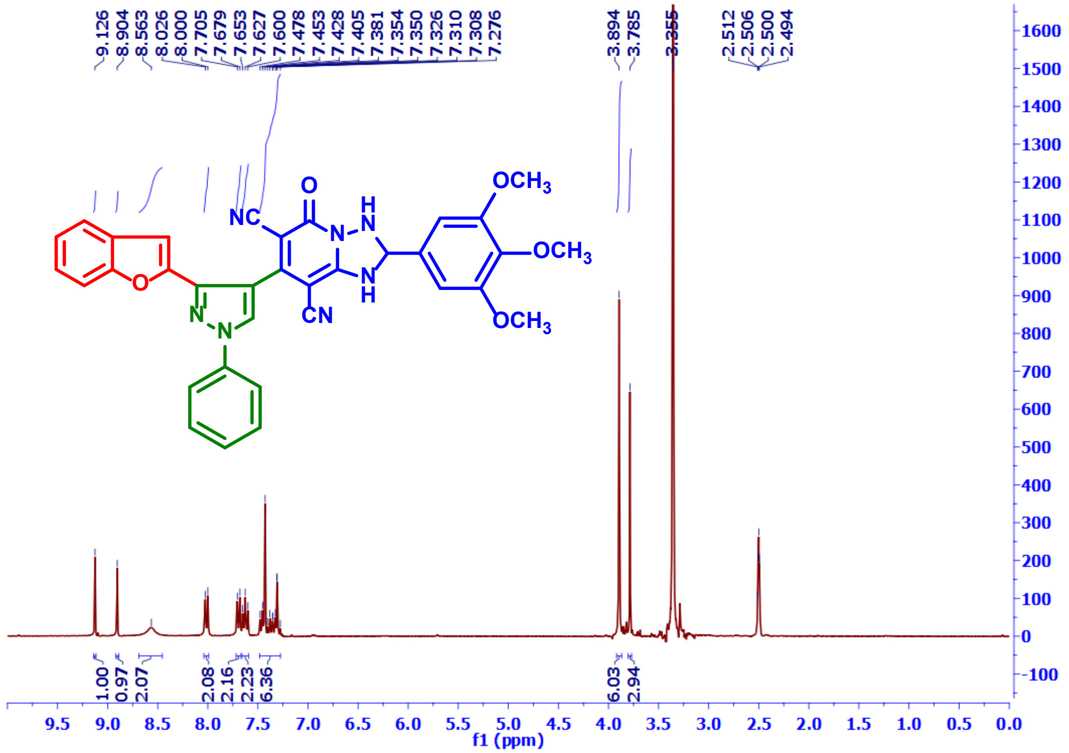

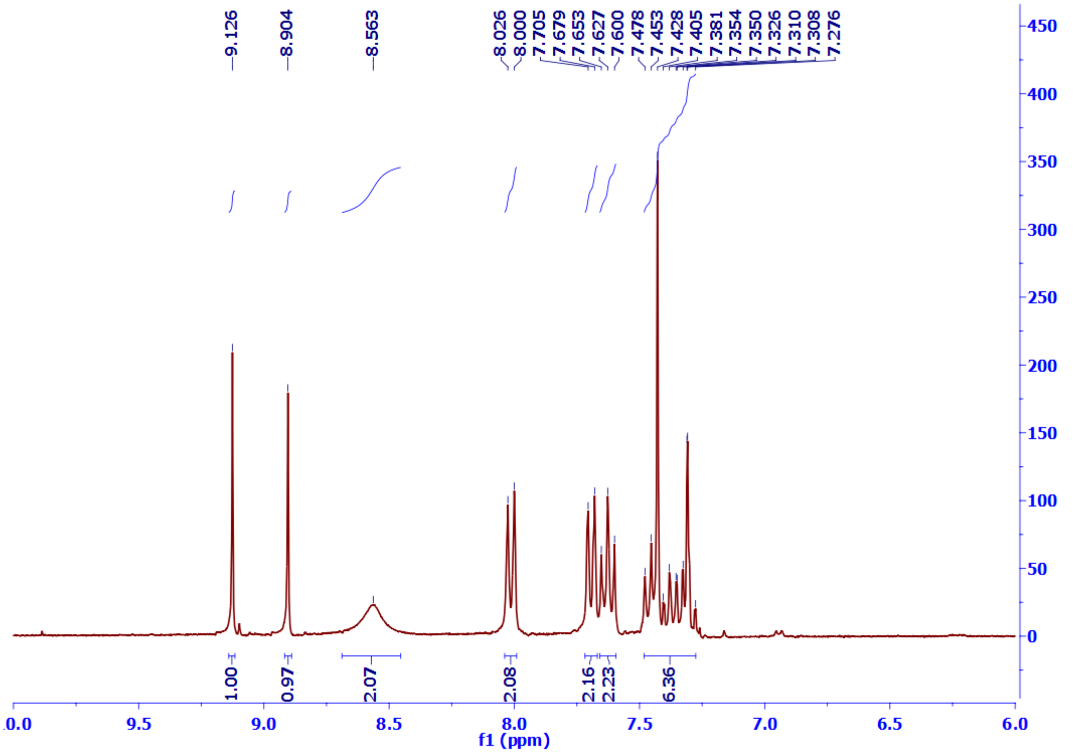

Figure S17: ^{1}H (300 MHz) NMR spectra of **11a** in DMSO-d_6

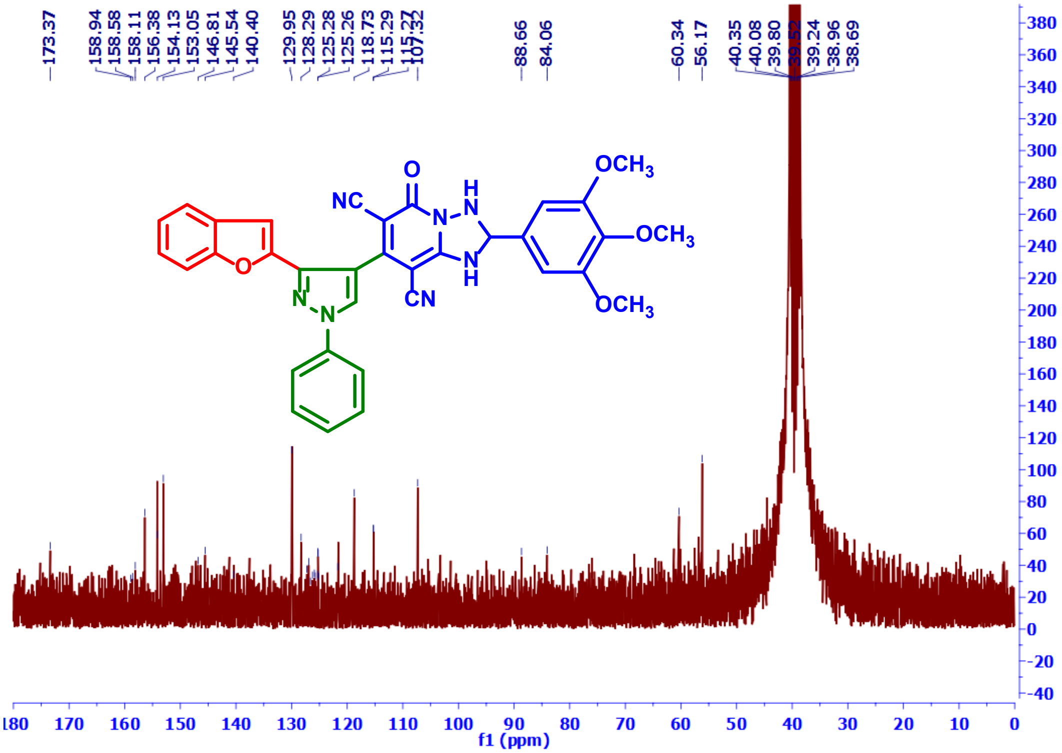

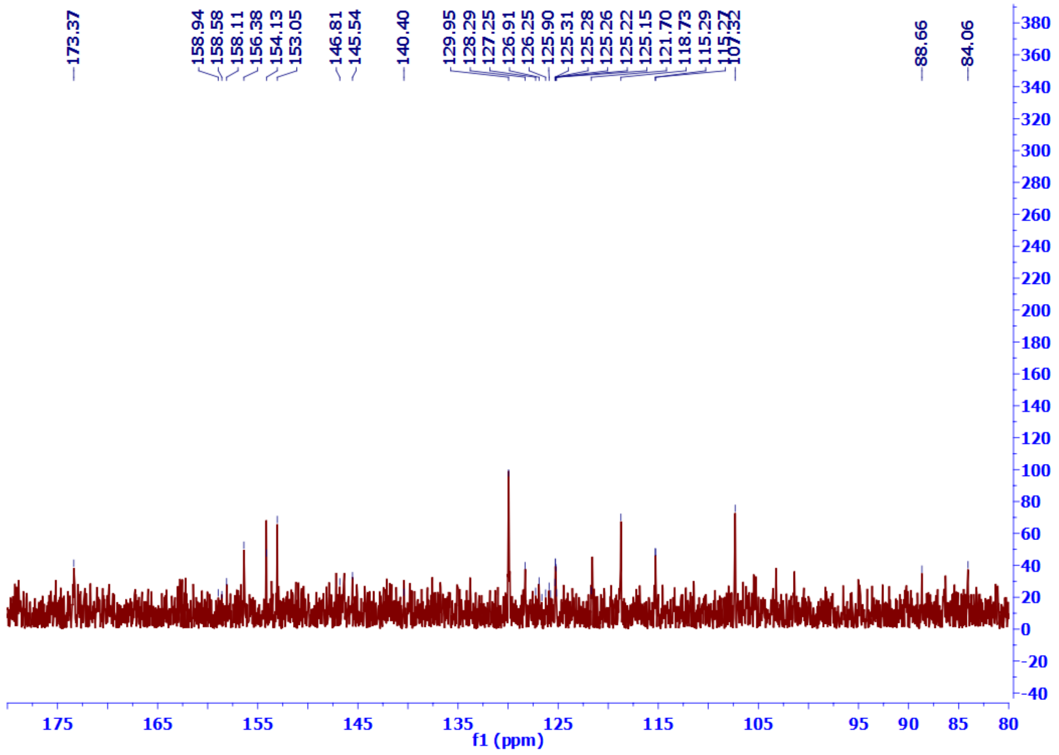

Figure S18: ^{13}C (75 MHz) NMR spectra of **11a** in DMSO-d_6

7-(3-(Benzofuran-2-yl)-1-phenyl-1H-pyrazol-4-yl)-2-(4-chlorophenyl)-1,2,3,5-tetrahydro-5-oxo-[1,2,4]triazolo[1,5-a]pyridine-6,8-dicarbonitrile (11b)

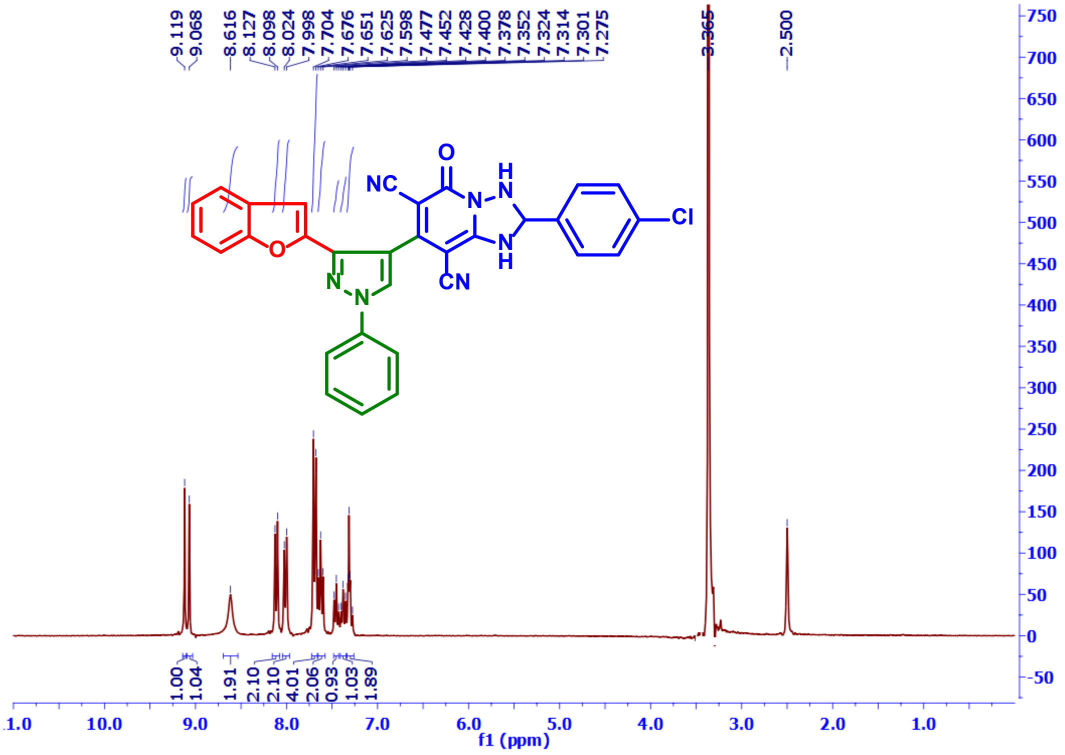

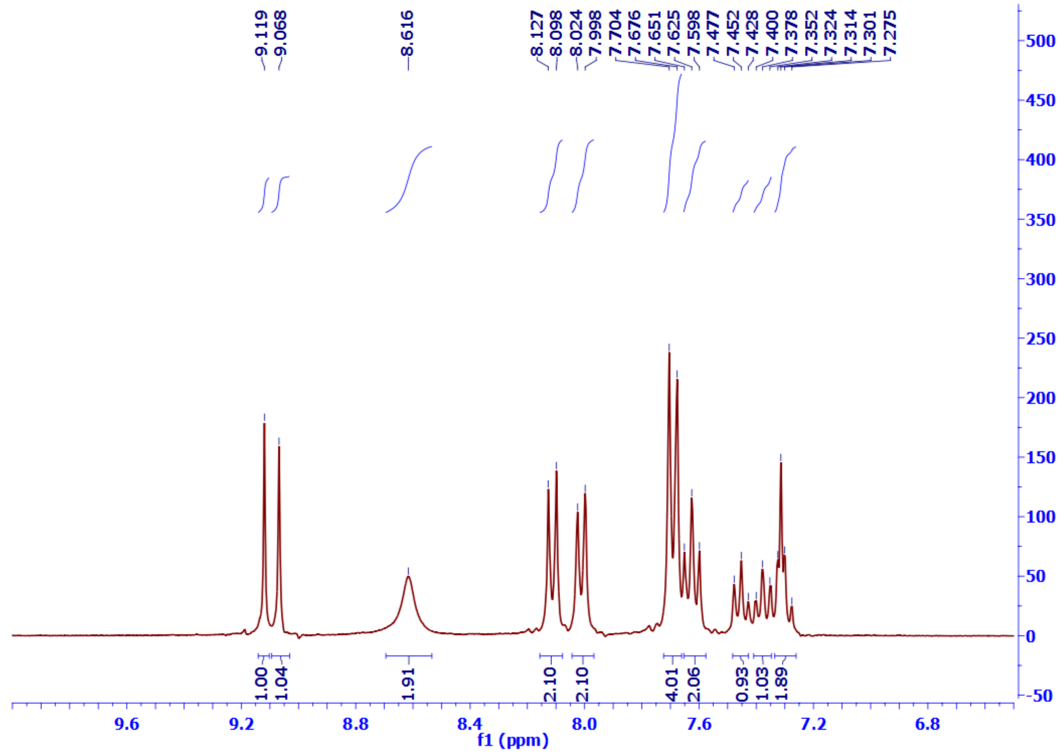

Figure S19: ^{1}H (300 MHz) NMR spectra of **11b** in DMSO-d_6

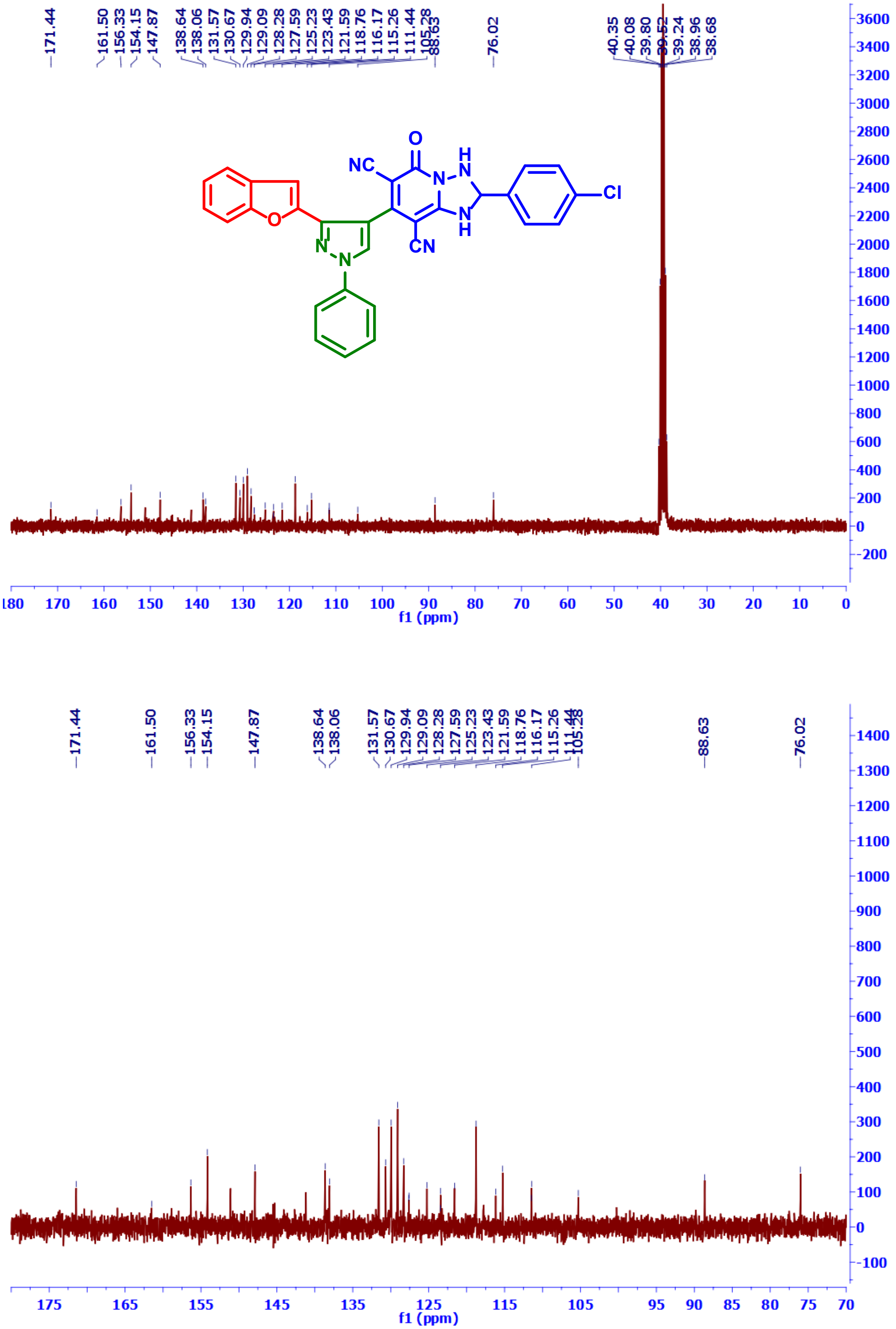

Figure S20: ^{13}C (75 MHz) NMR spectra of **11b** in DMSO-d_6

7-(3-(Benzofuran-2-yl)-1-phenyl-1H-pyrazol-4-yl)-1,2,3,5-tetrahydro-2-(5-methylfuran-2-yl)-5-oxo-[1,2,4]triazolo[1,5-a]pyridine-6,8-dicarbonitrile (11c)

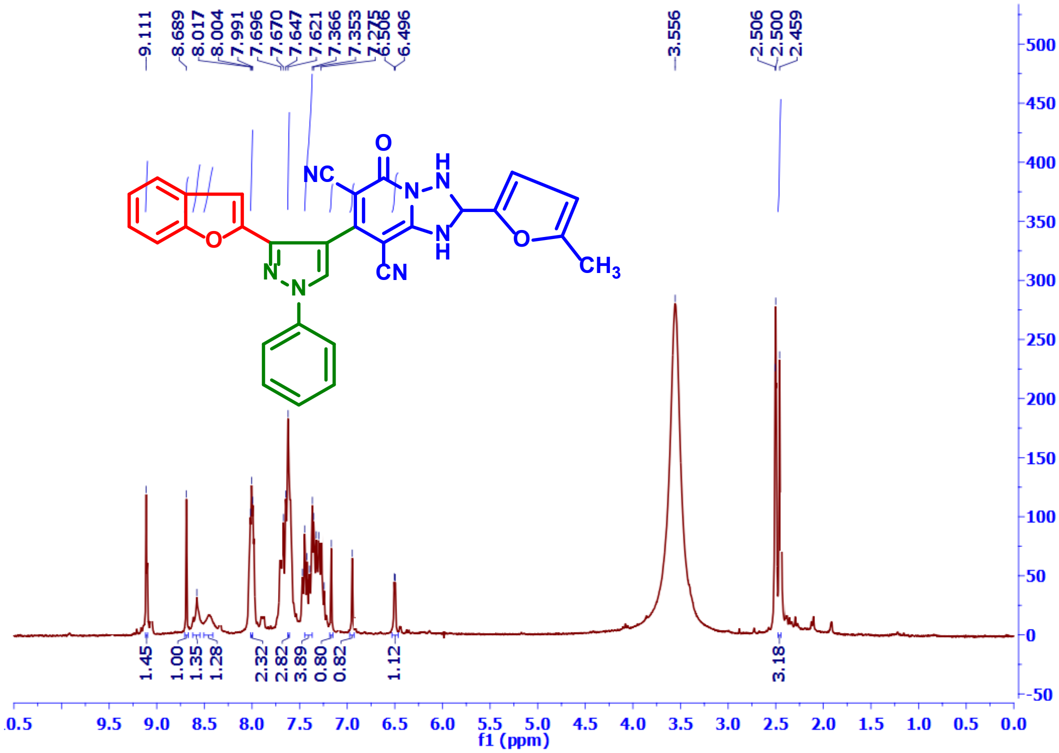

Figure S21: ^{1}H (300 MHz) NMR spectra of **11c** in DMSO-d_6

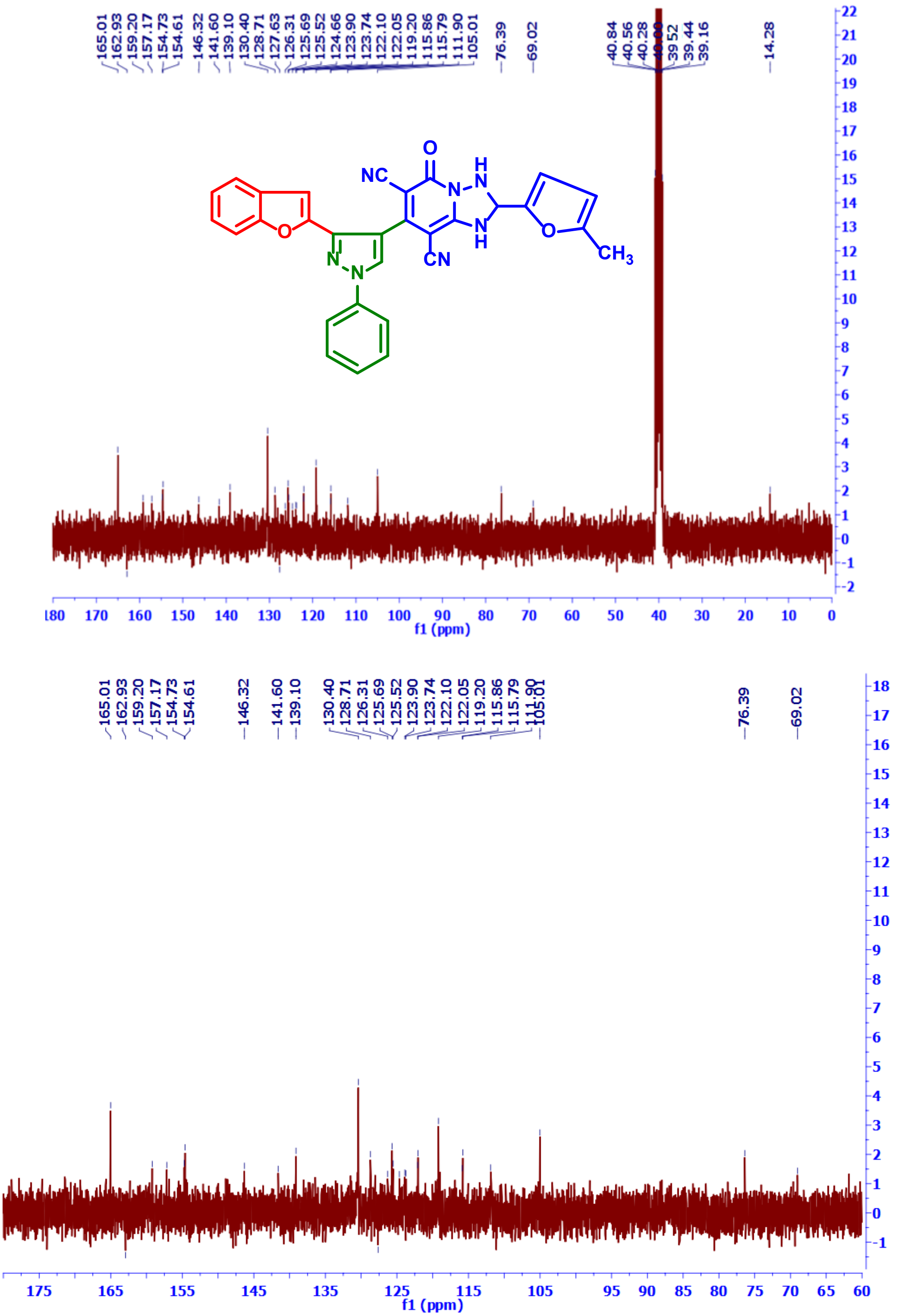

Figure S22: ^{13}C (75 MHz) NMR spectra of **11c** in DMSO-d_6

7-(3-(Benzofuran-2-yl)-1-phenyl-1H-pyrazol-4-yl)-1,2,3,5-tetrahydro-5-oxo-2-(thiophen-2-yl)-[1,2,4]triazolo[1,5-a]pyridine-6,8-dicarbonitrile (11d)

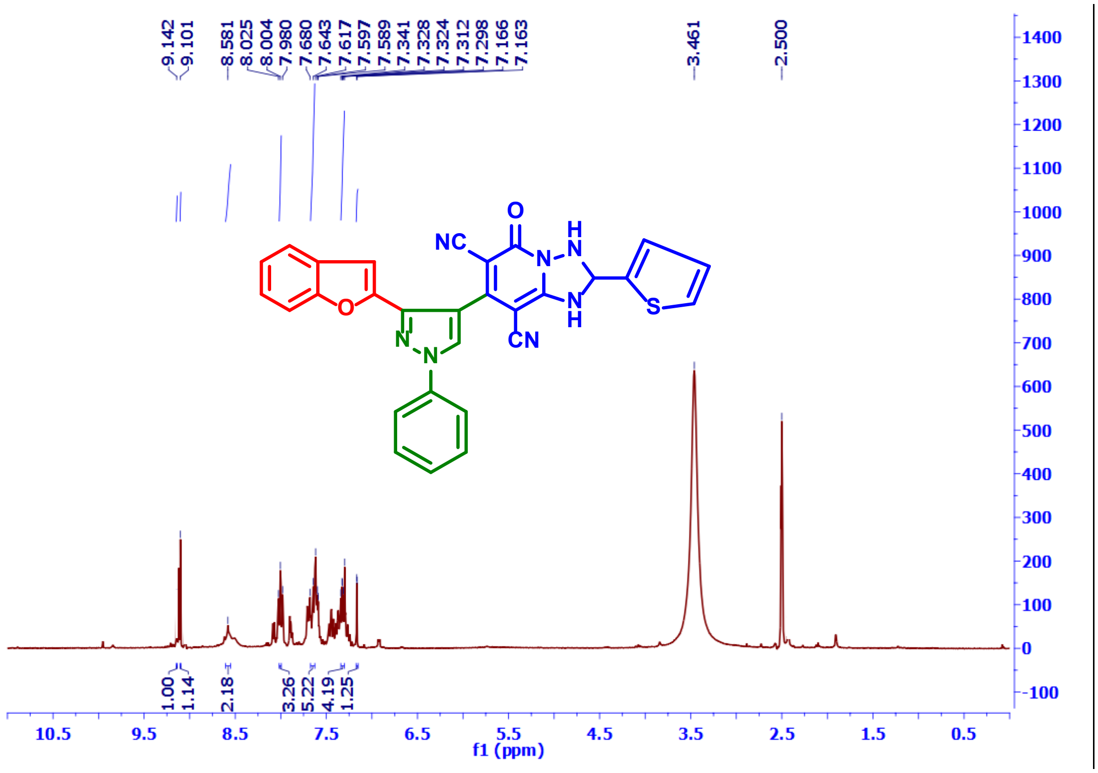

Figure S23: ^{1}H (300 MHz) NMR spectra of **11d** in DMSO-d_6

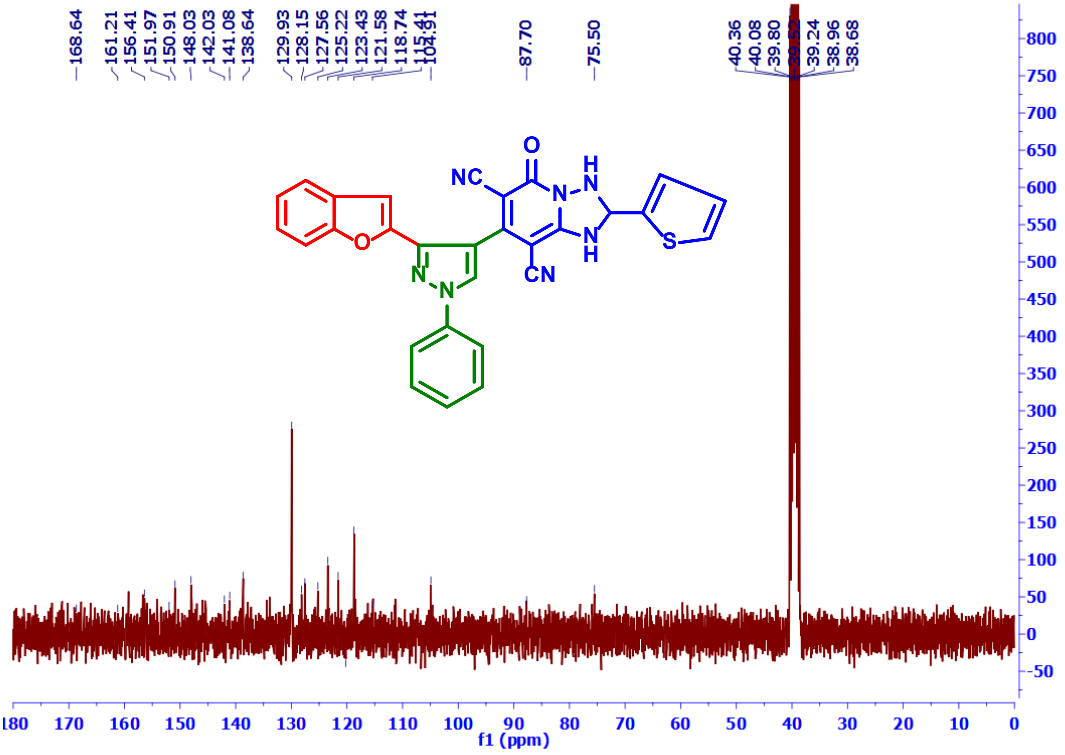

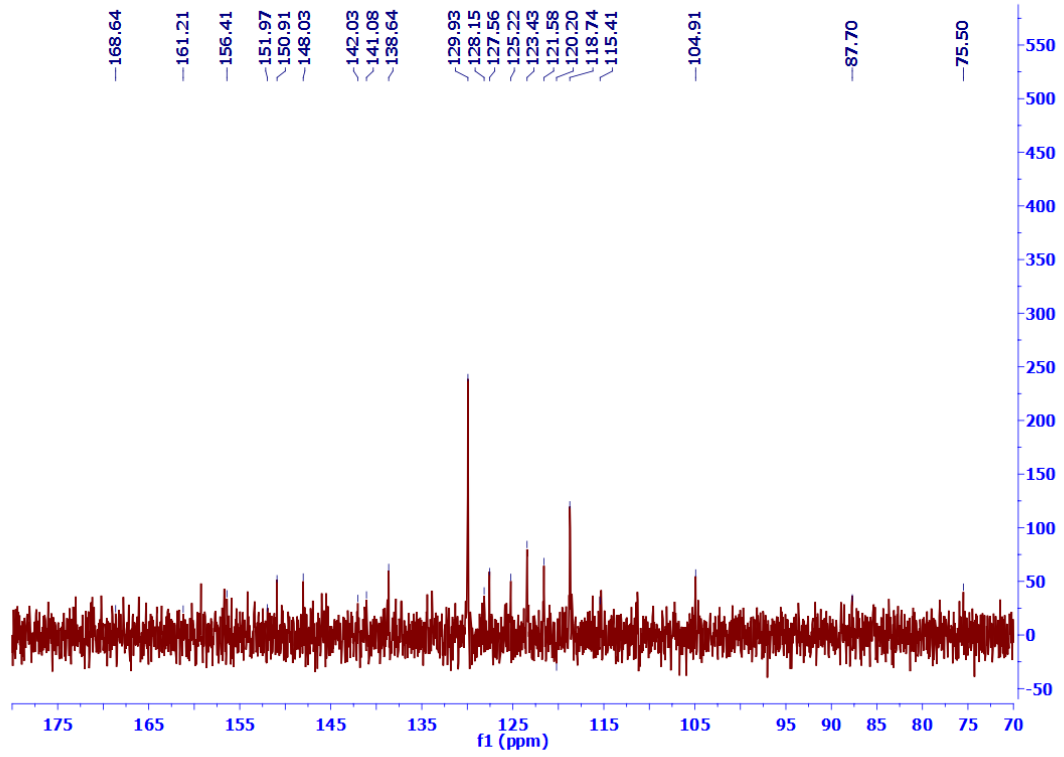

Figure S24: ^{13}C (75 MHz) NMR spectra of **11d** in DMSO-d_6

5.2.1. In vitro antimicrobial activity

5.2.1.1 Microorganisms

In vitro antimicrobial activity was examined for tested samples. The bacteria used in this study were Gram-positive bacterial strains (*Staphylococcus aureus* ATCC-6538, *Bacillus cereus* ATCC-11778, , Gram-negative bacterial strains (*Escherichia coli* ATCC-25922, *Pseudomonas aeruginosa* ATCC-27853 and fungi (*Fusarium solani*, and *Candida albicans* ATCC-10231 These micro-organisms were obtained from the American Type Culture Collection (ATCC, Rockville MD, USA), and Northern Utilization Research and Development Division, United State Department of Agriculture, Peoria, Illinois, USA (NRRL).The bacterial strains were revived for bioassay by sub-culturing in fresh nutrient broth medium for 24 hours before the test. While fungi were cultured on potato dextrose agar (PDA) for 7 days at 28°C before the experiment was carried out.

5.2.1.2. Inoculum Preparation

Stock cultures were maintained at 4°C on slopes of nutrient agar and potato dextrose agar. Active cultures for experiments were prepared by transferring a lapful of cells from the stock cultures to test tubes of Mueller-Hinton broth (MHB) (Lab M Limited, Bury, Lancashire, UK) for bacteria and Sabouraud dextrose broth (SDB) (Lab M., Bury, Lancashire, UK) for fungi that were incubated without agitation for 24 hours at 37 °C and 25 °C, respectively. To 5 ml of MHB and SDB, 0.2 ml of culture was inoculated and incubated (or diluted) till it reached the turbidity equal to that of the standard 0.5 McFarland solution at 625 nm (A = 0.08 to 0.1) which is equivalent to 1.5 x 10^8 cfu/ml.

5.2.1.3. Bioassay method

The antibacterial screening bioassay was made by the agar well diffusion method using Mueller-Hinton agar (Lab M Limited, Bury, Lancashire, UK), then the plates were transferred to refrigerator for 1 h at 4 °C. The experiment was conducted in duplicate. All

plates were incubated at 37°C for 24 hours for bacterial strains and 28-30 °C for 48 hours for fungal strain. Clearance zones around the wells were noted and measured in millimetres. Standard bacterial antibiotics as novobiocin (30 μg) and fungal drug as Clotrimazole (50 μg) were used as positive control for bacteria and fungi, respectively.

5.2.2. DPPH radical scavenging assay

The DPPH (1–diphenyl–2–picrylhydrazyl) scavenging activity of the sample was determined quantitatively according tp the reported method. A 500 μl of ethanolic DPPH solution (0.4 mmol) was mixed vigorously with 500 μl of sample (standardized to obtain a final OD600 of 1), or water (as a control) and incubated at 37°C in the dark for 1h. The absorbance of the mixture was measured spectrophotometrically at 517 nm. The scavenging activity was calculated according to the following equation; Scavenging activity (%) = [1–(As– Ab)/Ac] x100, whereas Ab, Ac and As are the absorbance of the blank (ethanol and sample), the control (DPPH and deionised water) and the sample (DPPH and sample), respectively. This experiment was conducted in duplicate; all values are expressed as means ± standard deviation and compared to the control. Statistical analysis is done as previously mentioned.

5.2.3. Human red blood cell stabilization method

The human red blood cell (HRBC) membrane stabilization method was used to study the *in vitro* anti-inflammatory activity of the new samples. Blood was collected from one healthy volunteer. The collected blood was mixed with equal volume of sterilized AL sever solution (2% dextrose, 0.8% sodium citrate, 0.5% citric acid and 0.42% sodium chloride in water). The blood was then centrifuged at 3000 rpm for 20 min and packed cells were separated. The packed cells were washed with isosaline (0.85%, pH 7.2) and a 10% v/v suspension was made with isosaline. This HRBC suspension was used for the estimation of anti-inflammatory property. One millilitre of sample and Diclofenac

sodium and aspirin were separately mixed with 1 ml of phosphate buffer (0.15 M, pH 7.4), 2 ml of hypo saline (0.36%) and 0.5 ml of HRBC suspension. Instead of sample, 2 ml distilled water was used as the control. All the assay mixture was incubated at 37^{0}C for 30 min and centrifuged at 3000 rpm for 20 min. The supernatant liquid was decanted and the haemoglobin content in the supernatant solution was estimated using spectrophotometer at 560 nm. Percentage hemolysis was estimated by assuming the hemolysis produced in the control as 100%.

The percentage hemolysis was calculated by using the following formula:

$$\% \text{ Hemolysis} = \left(\frac{OD\ sample}{OD\ control}\right) * 100$$

The percentage of HRBC membrane stabilization or protection was calculated by using the following formula:

$$\% \text{ Protection} = 100 - \left(\frac{OD\ sample}{OD\ control}\right) * 100$$

***5.2.4.* Enzyme assessment of *E.coli* DNA Gyrase**

The *in vitro* enzyme inhibition assessment for the most active derivatives **9**, **10** was carried out in the confirmatory diagnostic unit, Vacsera, Egypt. The screening performed against *E. coli* DNA gyrase was carried out using *E. coli* DNA gyrase microplate assay kit (Inspiralis) according to the optimized protocol by the manufacturer. The used reference drug was novobiocin according to the reported methods.

***5.2.5. In vitro* cytotoxicity assay**

The cytotoxic effect of test samples using WI38 cells was evaluated by MTT assay. Commercially available kit for in vitro toxicology MTT based assay, Sigma was used. Briefly, WI38 cells were grown as monolayer culture in DMEM (Invitrogen/Life Technologies) supplemented with 10% FBS (Hyclone), penicillin (100 μg/mL) and streptomycin (100 μg/mL) and maintained under an atmosphere of 5% CO_2 at 37 ^oC. Control cells were incubated for 48 h at 37 ^oC in culture medium. Cells were rinsed with

PBS and harvested by trypsinization and were plated in 96 well plates and incubated under 5% CO2 at 37 ^{o}C overnight. Different concentrations of test samples were used for the treatment of cells. All the test samples were removed after incubation for 48 h at 37 oC and 100 μL of MTT (5 mg/mL) was added and again incubated for 4 h at 37 ^{o}C and kept under dark condition. Then, 100 μL of MTT solubilizing solution was added 23 and incubated for 1 h at 37 ^{o}C. The absorbance was read at 590 nm using microtitre plate reader and cell viability was calculated. Chemicals and reagents were from Sigma, or Invitrogen.

E. coli **DNA gyrase assay:**

Detailed results

E.Coli	WL<br>=				
DNA Gyrase				conc	
	[redacted]				
code	IC50	ug		log	%inh
10			100	2	83.4

		50	1.7	55.3
		10	1	30.2
		1	0	8.15
		0.1	-1	4.42
EC				0
	[redacted]		[redacted]	

code	IC50	conc.uM	log conc	%inh
9		100	2	87.6
		50	1.7	63.3
		10	1	39
		1	0	15.8
		0.1	-1	7.28
EC				0
	[redacted]		[redacted]	

Code				
	[redacted]		[redacted]	
CIP	IC50	conc.uM	log conc	%inh
		100	2	94.2
		50	1.7	77.7
		10	1	53.7
		1	0	36.9
		0.1	-1	14.7
EC				0

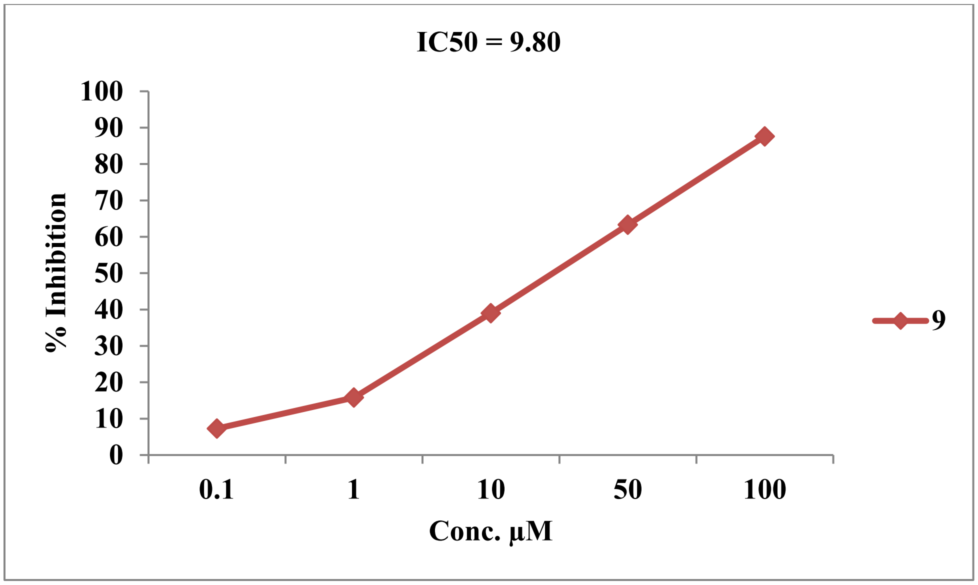

Figure S25: Determination of IC_{50} concentration of compound **9**

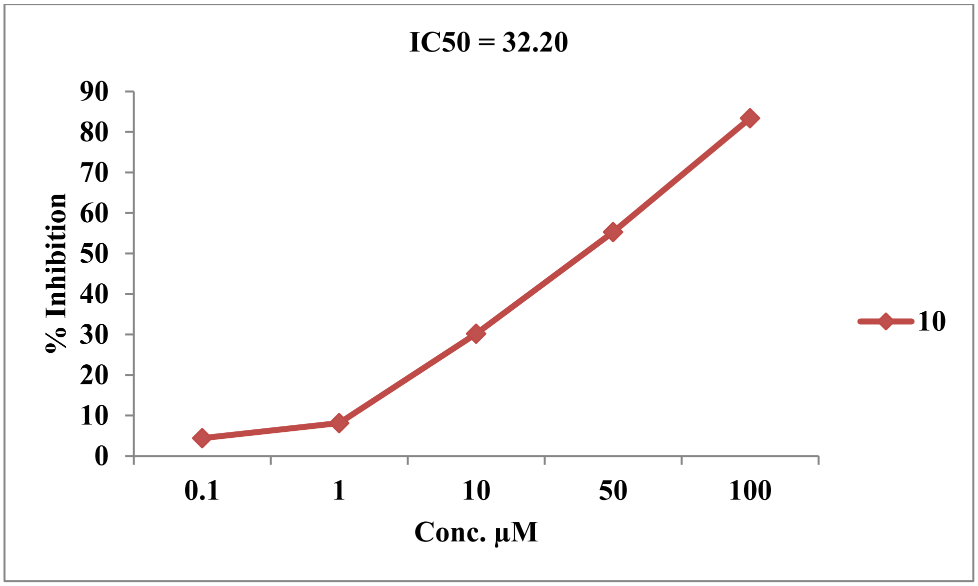

Figure S26: Determination of IC_{50} concentration of compound **10**

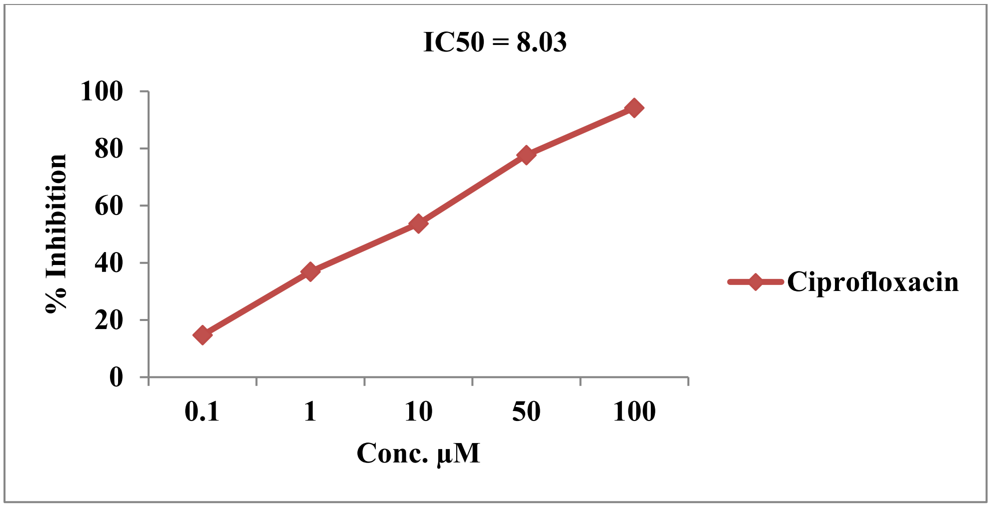

Figure S27: Determination of IC_{50} concentration of ciprofloxacin

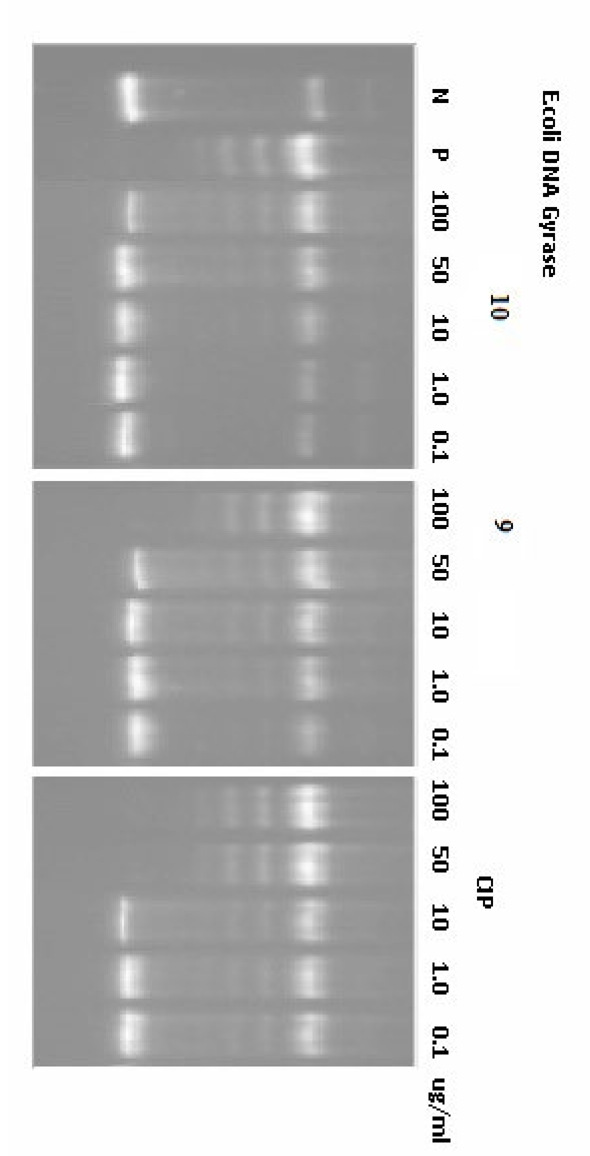
Ecoli DNA Gyrase
10
9
CIP
N P 100 50 10 1.0 0.1 100 50 10 1.0 0.1 100 50 10 1.0 0.1 ug/ml

5.3. ADMET studies

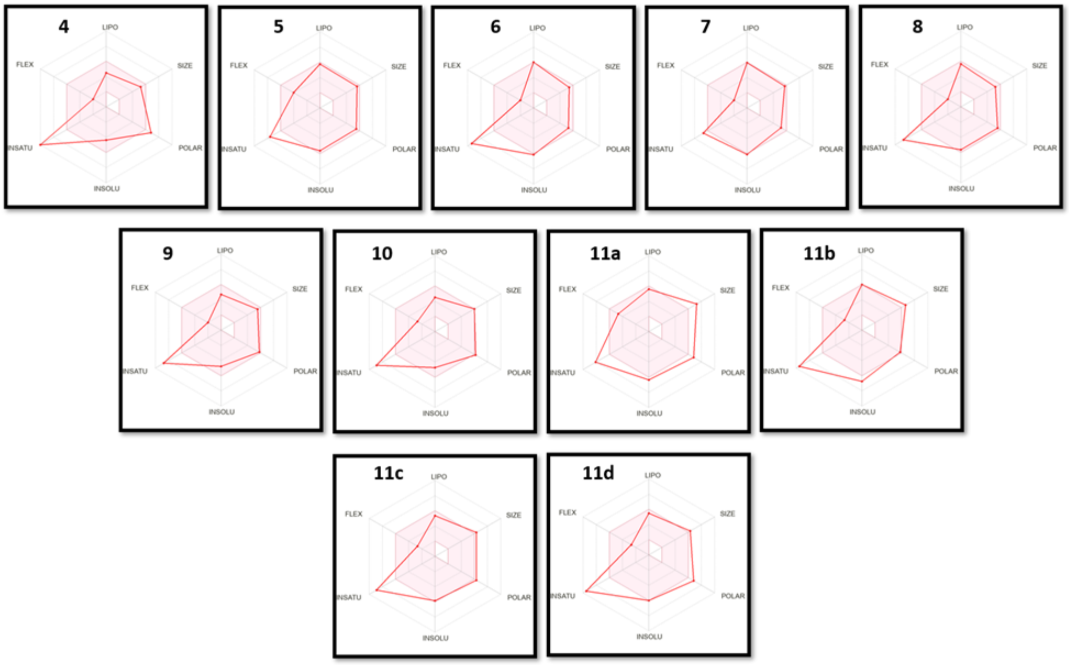

Figure S28: Bioavailability radar chart for the synthesized compounds

(The pink area indicates the accepted range for each of the measured parameter)
